# An Overview of Sucrose Synthases in Plants

**DOI:** 10.3389/fpls.2019.00095

**Published:** 2019-02-08

**Authors:** Ofer Stein, David Granot

**Affiliations:** Institute of Plant Sciences, Agricultural Research Organization, The Volcani Center, Rishon LeZion, Israel

**Keywords:** sucrose metabolism, sugar signaling, plant development, cellulose synthesis, callose synthesis, starch synthesis, meristem

## Abstract

Sucrose is the end product of photosynthesis and the primary sugar transported in the phloem of most plants. Sucrose synthase (SuSy) is a glycosyl transferase enzyme that plays a key role in sugar metabolism, primarily in sink tissues. SuSy catalyzes the reversible cleavage of sucrose into fructose and either uridine diphosphate glucose (UDP-G) or adenosine diphosphate glucose (ADP-G). The products of sucrose cleavage by SuSy are available for many metabolic pathways, such as energy production, primary-metabolite production, and the synthesis of complex carbohydrates. SuSy proteins are usually homotetramers with an average monomeric molecular weight of about 90 kD (about 800 amino acids long). Plant SuSy isozymes are mainly located in the cytosol or adjacent to plasma membrane, but some SuSy proteins are found in the cell wall, vacuoles, and mitochondria. Plant *SUS* gene families are usually small, containing between four to seven genes, with distinct exon-intron structures. Plant *SUS* genes are divided into three separate clades, which are present in both monocots and dicots. A comprehensive phylogenetic analysis indicates that a first *SUS* duplication event may have occurred before the divergence of the gymnosperms and angiosperms and a second duplication event probably occurred in a common angiosperm ancestor, leading to the existence of all three clades in both monocots and dicots. Plants with reduced SuSy activity have been shown to have reduced growth, reduced starch, cellulose or callose synthesis, reduced tolerance to anaerobic-stress conditions and altered shoot apical meristem function and leaf morphology. Plants overexpressing *SUS* have shown increased growth, increased xylem area and xylem cell-wall width, and increased cellulose and starch contents, making *SUS* high-potential candidate genes for the improvement of agricultural traits in crop plants. This review summarizes the current knowledge regarding plant SuSy, including newly discovered possible developmental roles for SuSy in meristem functioning that involve sugar and hormonal signaling.

## Introduction

Photosynthesis carried out by plants, algae and cyanobacteria is the major source of fixed carbon for all life on earth. In plant photosynthesis, carbon dioxide is fixed in the chloroplasts via the Calvin cycle to yield triose phosphates (triose-P). Triose-P can be transported to the cytosol by a triose-P/phosphate translocator. In the cytosol, two triose-P molecules produce one fructose 1,6-bisphosphate (F1,6BP) molecule in a reaction catalyzed by aldolase. F1,6BP is then further metabolized to yield other hexose phosphates, such as fructose 6-phophate (F6P) and glucose 6-phosphate (G6P). G6P can be used to form nucleotide sugars such as UDP-glucose (UDP-G), and UDP-G is combined with F6P to form sucrose 6-phosphate (sucrose-P) in a reaction catalyzed by sucrose phosphate synthase (SPS). Sucrose-P is dephosphorylated by sucrose phosphate phosphatase (SPP) to form sucrose (Suc), the primary product of photosynthetic tissues and the main sugar transported from the source tissues through the phloem to non-photosynthetic tissues (sink tissues) in most plants (Ruan, 2014). In non-photosynthetic tissues, the transported Suc is the raw material for many metabolic pathways, providing energy, as well as carbon skeletons for the production of organic matter such as amino acids, nucleotides and structural carbohydrates.

**FIGURE 1:**
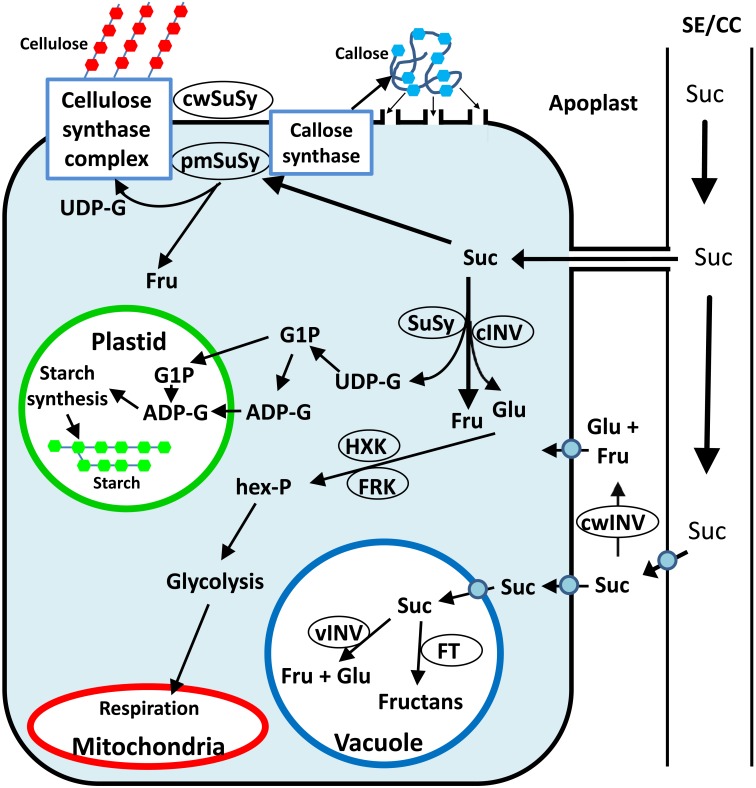
Simplified schematic presentation of sugar metabolism in sink tissue cells toward cellulose, callose and starch synthesis. Suc can be hydrolyzed in the apoplast by cwINV to yield Glc and Fru, which can be brought into the cell by a monosaccharide transporter. Alternatively, Suc can be brought into the sink cell by a Suc transporter or enter through plasmodesmata. Inside the cell, Suc can be stored in the vacuole or hydrolyzed by vINV. In the cytosol, Suc can be hydrolyzed by cytosolic INV to yield Glc and Fru, or cleaved by cytosolic SuSy to yield Fru and UDP-G. The hexoses can be phosphorylated to hexose phosphates (hex-P), directed to starch synthesis in the plastid or to glycolysis and then respiration in the mitochondria or directed to other metabolic pathways. Plasma membrane associated SuSy (pmSuSy) and cwSUS can generate UDP-G that is used in the synthesis of cellulose for cell walls and callose for plugging plasmodesmata.

Upon arriving in sink tissues, Suc can enter the sink cells via several different pathways (Ma et al., 2018). Suc can be unloaded from the phloem to the apoplast by Suc transporters. It can then enter the sink cells via Suc transporters or hydrolyzed by cell-wall invertase (cwINV) to yield glucose (Glc) and fructose (Fru), which can enter the sink cells via hexose transporters (Ruan, 2014). Suc can also pass directly from the phloem to sink cells through plasmodesmata (Figure 1). Inside sink cells, Suc can be metabolized or transported to the vacuole, where it can be stored as Suc, transformed into fructans by fructosyltransferases (FTs), or hydrolyzed by vacuolar invertase (vINV) and stored as hexoses (Figure 1). To be metabolized, Suc must be cleaved by either cytosolic invertase or SuSy (EC 2.4.1.13). While invertase (INV) catalyzes the irreversible hydrolyzation of Suc into its hexose monomers, Glc and Fru, SuSy catalyzes the reversible cleavage of Suc using UDP to yield fructose and UDP-G. This review focuses on all aspects of the SuSy found in plants, including their evolution, gene families, protein structure and tissue and subcellular localization, as well as their roles in plant development and sugar signaling.

## Structure and Enzymatic Activity of SuSy

Sucrose synthase belongs to the glycosyltransferase-4 subfamily of glycosyltransferases, a large family that includes a wide variety of glycosyltransferases, including SPS, trehalose synthase, and trehalose phosphorylase. [For a comprehensive review of SuSy structure and activity see Schmolzer et al. (2016)]. SuSy proteins are typically considered homotetramers (Schmolzer et al., 2016), although some SuSy isoforms have been reported to act as heterotetramers in barley (*Hordeum vulgare*) (Guerin and Carbonero, 1997), maize (*Zea mays*) (Duncan et al., 2006), rice (*Oryza sativa*) (Huang and Wang, 1998) and bird cherry (*Prunus padus*) (Sytykiewicz et al., 2008). Interestingly, the two SuSy enzymes from bird cherry are reported to be homo- and heterotetramers (Sytykiewicz et al., 2008). SuSy monomers average about 90 kDa in weight and 800 amino acids in length, but some SuSy isoforms are considerably different. For example, a banana (*Musa acuminata*) SuSy monomer was found to be 110 kDa (Yang and Su, 1980) and Arabidopsis (*Arabidopsis thaliana*) AtSUS6 was calculated to weigh 107 kDa (Baud et al., 2004). Other SuSy isoforms are much smaller, for example, the bird cherry SuSy monomers are estimated to be about 53, 58, and 63 kDa (Sytykiewicz et al., 2008). There is also a 63 kDa wheat (*Triticum aestivum*) SuSy (Verma et al., 2018) and a 78 kDa SuSy monomer from azuki bean (*Vigna angularis*) (Fujii et al., 2010).

A SuSy monomer is typically composed of two domains, an N-terminal domain of about 250 amino acids thought to be involved in cellular targeting and a C-terminal GT-B domain, usually made up of about 500 amino acids, which is responsible for the enzyme’s glycosyltransferase activity (Zheng et al., 2011). SuSy proteins contain two phosphorylation sites. The first site is a serine phosphorylation site at position 11 to 15, which is thought to play a role in membrane association (see section “ Subcellular Localization of SuSy”). The second site is also a serine, at around position 170, and is thought to regulate protein degradation (Hardin et al., 2003). *In vitro* phosphorylation of rice SuSy proteins, Rsus1-3 may promote SuSy activity (Takeda et al., 2017).

The tetrameric structure of plant SuSy was confirmed by the determination of the structure of Arabidopsis AtSUS1 by X-ray crystallography (Zheng et al., 2011). Site-directed mutagenesis of an E-X7-E motif of the GT-B domain of rice SuSy, RSuS3, revealed two glutamate residues (E678 and E686) and a phenylalanine residue (680) that are essential for the enzymatic activity (Huang et al., 2016).

Sucrose synthase is the only Suc-metabolizing enzyme that can catalyze both the synthesis of Suc from Fru and UDP-G and the cleavage of Suc, in the presence of UDP, to Fru and UDP-G. SuSy can also utilize other nucleotide phosphates for Suc cleavage, especially ADP, but usually with a lower affinity. The direction of SuSy activity may also be regulated by pH; its optimal Suc-synthesis activity is observed between pH 7.5 and 9.5 and optimal Suc degradation occurs at pH values between 5.5 and 7.5 (Schmolzer et al., 2016).

## Subcellular Localization of SuSy

Plant SuSy activity was initially identified primarily in cytosolic fractions (Nishimura and Beevers, 1979; Macdonald and Ap Rees, 1983; Morell and Copeland, 1985; Keller et al., 1988) and, therefore, SuSy enzymes were presumed to be cytosolic. The first evidence of non-cytosolic SuSy was found in cotton (*Gossypium hirsutum*) fibers, in which 50% or more of the SuSy protein was found to be tightly associated with the plasma membrane (Amor et al., 1995). Two plasma-membrane SuSy proteins were also detected in maize endosperm by activity assays and monoclonal antibodies; those proteins were also found in the cytosol (Carlson and Chourey, 1996). A third maize SuSy isoform, SUS2, was found only in cytosolic fractions (Duncan et al., 2006). Localization of SuSy from tobacco (*Nicotiana tabacum*) pollen tubes, revealed two isoforms, one of which is associated with the plasma membrane and the other one is associated with the plasma membrane and is also found in the cytosol (Persia et al., 2008). The co-localization of SuSy, callose synthase and cellulose synthase in tobacco pollen tubes further supports the enzyme’s dual role in callose and cellulose synthesis (Cai et al., 2011).

It has been suggested that the N-terminal SuSy phosphorylation site is a key factor in the mechanism of SuSy membrane association. In maize, phosphorylation of SuSy was found to result in decreased membrane association; whereas dephosphorylation was found to cause SuSy to be less soluble (Winter et al., 1997). However, other studies have shown that the phosphorylation state of SuSy in soybean (*Glycine max*) nodules has no marked effect on membrane association (Komina et al., 2002) and that overexpression of a mutant SuSy from mung bean (*Vigna radiata*) that lacks the phosphorylation site (S11E) does not affect the ratio of soluble to membrane-bound SuSy (Konishi et al., 2004), indicating that the N-terminal serine residue might not be critical for the subcellular localization. Similarly, in a study on a castor bean (*Ricinus communis*) SuSy, RcSUS1, phosphorylation of S11 did not affect the partitioning between soluble and membrane-bound SuSy, but did increase the amount of phosphorylated RcSUS1 under conditions that led to reduced SuSy protein levels, suggesting that S11 phosphorylation may protect RcSUS1 from proteolysis (Fedosejevs et al., 2014).

Sucrose synthase proteins association to the plasma membrane is considered to be strong as the use of 0.5M NaCl, 0.1–1% triton X-100, 25 nm EDTA and other detergents barely solubilized SuSy from the plasma membranes (Amor et al., 1995; Carlson and Chourey, 1996). Only the use of strong detergents such as digitonin, CHAPS or SDS solubilized SuSy completely (Amor et al., 1995).

The strong association of SuSy to the plasma membrane and a transmembrane prediction analysis led the researchers to hypothesize that part of the SuSy may be transmembrane (Carlson and Chourey, 1996). On the other hand, Amor et al. (1995) concluded that SuSy is not a transmembrane protein because it was not partitioned into triton X-114. It is more likely that SuSy are interacting with other proteins and form a complex that is also associated with the cellulose synthases or callose synthases located to membranes (Persia et al., 2008; Fujii et al., 2010).

A few SuSy isoforms have also been detected in cell walls. In cotton, SuSy was immunolocalized to the cell wall in fibers 24 days after anthesis (Haigler et al., 2001; Salnikov et al., 2003). The cell-wall fiber SuSy isoform, SusC, was found in cell walls during secondary cell wall synthesis and may play a role in the synthesis of cellulose (Brill et al., 2011). A cell wall-associated SuSy was also observed in tobacco pollen tubes using immunolocalization (Persia et al., 2008). The SusC sequence has a truncated N-terminus and a C-terminus that is different from that of the other cotton SuSy isoforms, and the mechanism by which it is localized to the cell wall remains unclear (Brill et al., 2011).

Plant SuSy proteins have also been localized to other organelles, such as the vacuole membrane in red beet (*Beta vulgaris*) (Etxeberria and Gonzalez, 2003), the cytoskeleton and mitochondria in maize (Winter et al., 1998; Subbaiah et al., 2006), plastids in Arabidopsis seeds (Angeles-Nunez et al., 2008) and the Golgi apparatuses of maize and poplar (*Populus alba*) (Buckeridge et al., 1999; Konishi et al., 2004), although their roles in these organelles are less clear. In red beet, it was estimated that 7% of SuSy protein may be tonoplast-bound and it was suggested that SuSy may play a role in the mobilization of Suc from the vacuole (Etxeberria and Gonzalez, 2003). Arabidopsis SuSy isoform, SUS2, was immunolocalized to plastid membranes of maturing seeds and it was suggested that this enzyme may play a role in directing carbon to plastid starch or lipid synthesis (Angeles-Nunez et al., 2008). Two maize SuSy isoforms, SUS1 and SH1, were detected in mitochondria using fractionation and immunolabeling and it was suggested that they may play a role in regulating solute fluxes between the mitochondria and cytosol (Subbaiah et al., 2006). Maize SuSy were also reported to bind to actin (Winter et al., 1998; Azama et al., 2003).

## *Sus* Gene Families

The first *SUS* gene to be cloned and sequenced was the *Shrunken* (*Sh*) gene from maize (Werr et al., 1985). Since then, many other *SUS* genes have been cloned from different plants, including another maize *SUS* (McCarty et al., 1986; Shaw et al., 1994) and genes from Arabidopsis (Chopra et al., 1992; Martin et al., 1993), rice (Wang et al., 1992; Yu et al., 1992), potato (*Solanum tuberosum*) (Fu et al., 1991; Fu and Park, 1995) and tomato (*Solanum lycopersicum*) (Goren et al., 2011). Recent advances in plant genome sequencing and assembly and the publication of new draft genomes have allowed the *SUS* gene family to be characterized in many plant species and in a more comprehensive manner.

The number of *SUS* genes varies considerably between plant species. In Arabidopsis, six *SUS* genes have been characterized (Baud et al., 2004), similarly, six *SUS* genes have been identified in each of the following species: rice (Hirose et al., 2008), tomato (Goren et al., 2017), rubber tree (*Hevea brasiliensis*) (Xiao et al., 2014), cacao (*Theobroma cacao* L.) (Li et al., 2015), peach (*Prunus persica*) (Zhang et al., 2015) and *Nicotiana sylvestris* (Wang et al., 2015). Seven SUS genes have been identified in cotton (*Gossypium arboreum*) (Chen et al., 2012), bamboo (*Bambusa emeiensis*) (Huang et al., 2018) and *Nicotiana tomentosiformis* (Chen et al., 2012; Wang et al., 2015; Huang et al., 2018). Only five genes have been characterized in grape (*Vitis vinifera*) and sugarcane (*Saccharum* spp.; Zhang et al., 2013; Zhu et al., 2017). In apple (*Malus domestica*), 11 SUS genes have been found (Tong et al., 2018). Fourteen *SUS* genes have been discovered in tobacco (*Nicotiana tabaccum*) (Wang et al., 2015) and 15 *SUS* genes have been identified in poplar (*Populus trichocarpa*) (An et al., 2014). In Chinese pear (*Pyrus bretschneideri* Rehd.), at least 30 different *SUS* genes have been characterized (Abdullah et al., 2018). However, at least five of the Chinese pear *SUS* genes cannot be functional, as the predicted proteins are too short to contain both the SuSy domain and the glycosyl-transferase domain.

Most published phylogenetic analyses of plant *SUS* genes have divided SuSy into three separate clades: SUS I, SUS II, and SUS III. Oddly, in many of these papers, only the SUS I clade included a clear separation between eudicot and monocot species; whereas in the other clades, and the SUS II clade in particular, there was no clear separation between monocots and eudicots (Chen et al., 2012; Xiao et al., 2014; Li et al., 2015; Wang et al., 2015; Zhang et al., 2015; Zhu et al., 2017). These unique phylogenetic trees raise fundamental questions about the evolution of SuSy in plants. However, it is important to note that some of these trees were created using limited numbers of monocot or dicot species. This situation encouraged us to create a more comprehensive phylogenetic tree.

**FIGURE 2:**
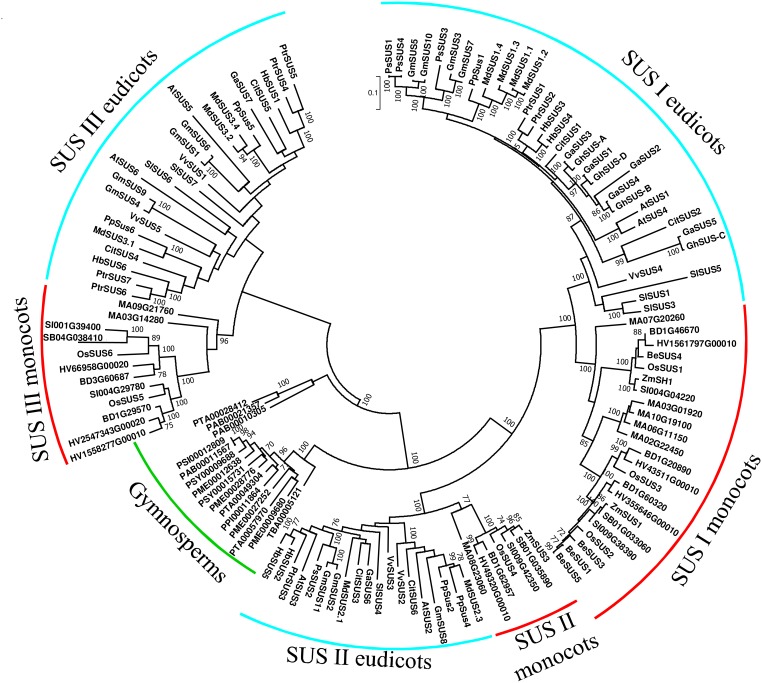
Phylogenetic tree of SUS genes from land plants. SuSy amino acid sequences were obtained from PUBMED using gene or protein IDs from previous studies (Baud et al., 2004; Hirose et al., 2008; Chen et al., 2012; Xiao et al., 2014; Zhang et al., 2015; Zhu et al., 2017; Abdullah et al., 2018; Huang et al., 2018; Tong et al., 2018). Additional amino acid sequences were retrieved using the Plaza 3.0 tool for gene-family analysis (Van Bel et al., 2017) using “sucrose synthase” in a keyword search. Partial sequences and sequences with substantial deletions were excluded, leaving a total of 133 sequences (Supplementary File 1). Sequences were aligned using MUSCLE with default options and analyzed in MEGA 7.0 (Kumar et al., 2016). The tree was created using the maximum-likelihood method based on the JTT matrix-based model (Jones et al., 1992). Bootstrap values >70% are denoted at the nodes. Gymnosperm species are labeled with a green arc. Turquoise arcs indicate eudicot species and red arcs indicate monocot species.

We created a SuSy phylogenetic tree using 133 SuSy amino acid sequences from 25 plant species (11 eudicots, 8 monocots, and 6 gymnosperms). Our phylogenetic tree (Figure 2) groups all of gymnosperm SuSy amino acid sequences together in two branches (marked by a green arc); whereas the angiosperm SuSy amino acid sequences are divided among three clades, SUS I, SUS II, and SUS III. Each clade is divided into two sub-clades: monocots (marked with red arcs) and eudicots (marked with turquoise arcs). The SUS I clade is the largest clade, which suggests that it might be more functionally important than the other clades, leading to greater conservation of *SUS* genes from that clade. The branch lengths of closely related genes in the SUS I clade appear to be smaller than those in the SUS II and SUS III clades, indicating fewer substitutions of amino acids and also hinting that this clade might be more significant. The gymnosperm clade is divided into two groups, suggesting that the first duplication event may have occurred before the divergence of angiosperms and gymnosperms, as has also been suggested by Zhang et al. (2011). A second duplication and speciation event probably resulted in the separation of the SUS I and SUS II clades. The separation of monocots and eudicots in these clades suggests that the duplication occurred in a common angiosperm ancestor, also reported by Zhang et al. (2011).

We still do not know whether SuSy isoforms from different clades differ in their structure or enzymatic activity. Arabidopsis knock-out mutants are available for all *SUS* genes and double mutants for clade-specific SuSy isoforms have been created. None of those mutants exhibit any significant phenotype that suggesting redundancy between the different clades (Bieniawska et al., 2007). Researchers have speculated that the main differences between the clades may be their expression patterns in different tissues and cells (Bieniawska et al., 2007).

## Role of SuSy in Sink Strength

Sink strength can be defined as the ability of an organ to import photoassimilates. Sink strength mainly relies on two parameters, sink organ size as a physical constraint and activity as a physiological constraint (Ho, 1988). Although imported photoassimilate can be used for respiration, sink-strength estimations are mainly based on net weight gain (Ho, 1988). Phloem loading is thought to be highly important for defining sink strength and the breakdown of Suc in sink organs may also contribute to sink strength. Work with promoter-GUS fusions has revealed *SUS* promoter activity in the phloem of many plant species, including potato (Fu and Park, 1995), Arabidopsis (Martin et al., 1993; Bieniawska et al., 2007), maize (Yang and Russell, 1990), rice (Shi et al., 1994), tomato (Goren et al., 2017) and *Craterostigma plantagineum* (Kleines et al., 1999). Work with isoform-specific antibodies has revealed that specific SuSy isoforms are more abundant in the phloem. In Arabidopsis, *AtSUS5* and *AtSUS6* are reported to be phloem specific (Barratt et al., 2009). SuSy proteins have also been detected in citrus (*Citrus paradisi*) and maize phloem companion cells using immunohistological analysis (Nolte and Koch, 1993). However, SuSy appear to localize to the phloem not only in the Suc unloading zones, but also in loading zones in mature leaves of citrus, maize, rice, Arabidopsis, and poplar (Nolte and Koch, 1993; Regmi et al., 2016), which suggests that SuSy probably play a general role in the phloem that is not limited to phloem unloading. In the phloem, SuSy may play a role in the maintenance of equilibrium between Suc and its breakdown products, supplying hexoses for energy production in companion cells and substrates for complex carbohydrates, like callose (Nolte and Koch, 1993). There is plenty of evidence that SuSy, and not INV, is the primary active enzyme in actively growing sink organs of different species, such as potato tubers, cassava (*Manihot esculenta*) roots, lima bean (*Phaseolus lunatus*) seeds, and tomato fruits (Morrell and Rees, 1986; Sung et al., 1989; Sun et al., 1992; Wang et al., 1994). Numerous studies involving plants that have altered SuSy activity and exhibit altered growth rates and weight gain in their sink organs support the putative role of SuSy in sink strength. In maize, the *shrunken* (*sh*) mutant is characterized by a 90% reduction in endosperm SuSy activity, low seed weight and a shrunken-seed phenotype (Chourey and Nelson, 1976). Pea (*Pisum sativum*) SuSy mutants (*rug4*) also exhibited significantly reduced SuSy activity in their embryos, reduced seed weight and a wrinkled-seed phenotype (Craig et al., 1999). Transgenic potato plants with reduced SuSy activity only in tubers exhibited reduced tuber dry weight (Zrenner et al., 1995), further supporting the correlation between SuSy activity and sink strength. SuSy activity was also suggested as an indicator for high rice grain yield in rice breeding programs (Counce and Gravois, 2006). A recent study found that the Suc-cleavage activity of a castor bean SuSy, RcSUS1, is inhibited by trehalose 6-phosphate, suggesting another mechanism by which Suc flux can be controlled in heterotrophic tissues (Fedosejevs et al., 2018).

Other studies have produced somewhat contradictory evidence for the role of SuSy in sink strength. In transgenic tomato plant with antisense suppression of *SUS*, reduced Suc import was observed only in very young fruits (7 days after anthesis) and not during the later starch-accumulation phase (23 DAA; D’Aoust et al., 1999). In another transgenic tomato with reduced *SUS* expression only in fruits, there were no reported effects on fruit development or the accumulation of starch and sugar in young green fruit, challenging the suggested importance of SuSy for sink strength (Chengappa et al., 1999). In Arabidopsis, *AtSUS2* and *AtSUS3* mutants had altered metabolism and accumulated less transient starch during seed development, with no effect on agronomic traits like seed weight and oil content (Angeles-Nunez and Tiessen, 2010). Interestingly, even a double mutant (*sus2* and *sus3*) and a quadruple mutant (*sus1*, *sus2*, *sus3*, and *sus4*) did not show any seed-related phenotypes (Bieniawska et al., 2007; Barratt et al., 2009), suggesting that Arabidopsis SuSy are not important for seed sink strength. Overall, the data suggest that SuSy might be important for sink strength, especially in starch-accumulating organs, although that role is probably not conserved in all plants.

## Role of SuSy in Starch Synthesis

For many decades, researchers have thought that SuSy may play important roles in the conversion of Suc into starch. The first genetic evidence for this came from the characterization of the maize *sh* mutant. The *sh* mutant exhibits a 90% reduction in SuSy activity in the endosperm, with no effect on SuSy activity in the embryo, and is also characterized by a 40% reduction in starch accumulation in the endosperm during development and at maturity (Chourey and Nelson, 1976). Similarly, a pea SuSy mutant (*rug4*) also showed reduced seed starch content (Craig et al., 1999).

Other studies involving transgenic plants with suppressed *SUS* expression have demonstrated reduced starch accumulation in potato tubers (Zrenner et al., 1995) and carrot (*Daucus carota*) taproots (Tang and Sturm, 1999), further supporting the hypothesized connection between SuSy and starch synthesis. Suc can be converted into starch via different pathways, which also differ between chloroplasts and heterotrophic tissues (For a comprehensive review of starch synthesis, see Bahaji et al., 2014b).

Adenosine diphosphate glucose is the main molecule converted into starch by the starch synthases in plastids. In chloroplasts, the main starch-synthesis pathway starts with two molecules of triose-P produced by photosynthesis, which yield F1,6BP. F1,6BP is transformed to F6P by fructose bisphosphate phosphatase to F6P, which is then converted into G6P by phosphoglucoisomerase (PGI). G6P is converted into glucose 1-phosphate (G1P) by the plastidic phosphoglucomutase (PGM). G1P is then converted into ADP-G, in a reaction catalyzed by ADP-G pyrophosphorylase (AGPase). This chloroplast starch synthesis pathway does not require Suc cleavage and, therefore, cytosolic SuSy is not expected to play an important role in leaf starch synthesis. The main genetic evidence supporting this claim is that the plastidic PGI, PGM and AGPase mutants, and plants with reduced expression of these genes are either starchless or contain very low levels of starch in their leaves (Bahaji et al., 2014b). However, there is also growing line of evidence suggesting that SuSy might play some role in leaf starch synthesis. Although UDP is considered to be the preferred nucleotide phosphate used by SuSy for Suc cleavage, ADP can also be effectively used by SuSy to form Fru and ADP-G (Baroja-Fernandez et al., 2003, 2012). Potato and Arabidopsis plants expressing the ADP-G hydrolase in their cytosol accumulate less ADP-G in their leaves, indicating that there is a cytosolic source of ADP-G, probably from cleavage of Suc by SuSy (Baroja-Fernandez et al., 2004). ADP-G levels in leaves of AGPase- and pPGM-mutant plants are comparable with those seen in the WT, indicating that AGPase is not the only source of ADP-G (Munoz et al., 2005; Bahaji et al., 2011, 2014a). Indeed, overexpression of Arabidopsis *SUS* in tobacco results in increased leaf starch (Bahaji et al., 2011; Nguyen et al., 2016). Based on this and many other studies, Bahaji et al. (2014b) suggested a chloroplast starch synthesis model in which (1) Suc cleavage by SuSy produces cytosolic ADP-G which is transported to the chloroplast for starch synthesis and (2) plastidic PGM and AGPase are recycling Glc units derived from starch breakdown back to starch. According to this model, the rate of starch accumulation is determined by the rate of cytosolic SuSy activity that yields ADP-G, cytosolic ADP-G transport to the chloroplast, starch synthesis, starch breakdown and the efficiency of the recycling of the products of the breakdown of starch.

There is much more evidence linking SuSy to starch synthesis in non-photosynthetic tissues or sink tissues. For example, a reduction in SuSy activity reduced starch content in potato tubers, carrot taproots and maize endosperm (Chourey and Nelson, 1976; Zrenner et al., 1995; Tang and Sturm, 1999). In addition, five maize starch-deficient endosperm mutants were screened for metabolic enzyme activity and all showed reduced SuSy activity (Doehlert and Kuo, 1990). However, because 90% suppression of SuSy activity in maize endosperm resulted in only a 40% reduction in starch, doubts were raised as to whether or not SuSy is directly involved in starch synthesis in sink tissues. SuSy from potato tubers and barley endosperm were shown to have similar Km values for the nucleotides UDP and ADP at saturated Suc levels. This fact, the greater activity of SuSy, as compared with AGPase, in barley seeds, and the reduced levels of ADP-G observed in potato tubers with repressed *SUS* together suggest that Suc cleavage by SuSy directly supplies ADP-G for starch synthesis (Baroja-Fernandez et al., 2003). Overexpression of *SUS* in potato tubers increased UDP-G and ADP-G levels and increased starch accumulation and yield (Baroja-Fernandez et al., 2009). Similarly, overexpression of *SUS* resulted in higher ADP-G levels and starch accumulation in maize endosperm (Li et al., 2013), and Arabidopsis T-DNA mutants for *AtSUS2* and *AtSUS3* exhibited reduced transient starch accumulation in seeds during early to mid-development (Angeles-Nunez and Tiessen, 2010). All these observations support the role of SuSy in starch accumulation.

## Role of SuSy in Vascular Tissues

There is a lot of evidence that *SUS* are highly expressed in vascular tissues. *SUS* promoters are expressed in the vasculature of many plant species, mostly in the phloem (Yang and Russell, 1990; Martin et al., 1993; Shi et al., 1994; Fu and Park, 1995; Kleines et al., 1999; Bieniawska et al., 2007; Goren et al., 2017). *In situ* hybridization has revealed the accumulation of *AtSUS5* and *AtSUS6* specifically in the phloem of Arabidopsis roots and hypocotyls (Barratt et al., 2009). SuSy protein has also been immunolocalized to the phloem companion cells in citrus and maize (Nolte and Koch, 1993) and in leaves of 9-day-old barley seedlings (Guerin and Carbonero, 1997). SuSy is also the main enzyme metabolizing Suc in the phloem of *Ricinus communis* (Geigenberger et al., 1993).

Sucrose synthase may play a number of different roles in the phloem involving the regulation of sink strength and phloem unloading (see section “ Role of SuSy in Sink Strength”), supplying hexoses for companion cell respiration and supplying precursors for complex carbohydrates, such as callose and cellulose (see section “Roles of SuSy in Cellulose and Callose Metabolism”). Only a few studies have used mutant and transgenic plants to elucidate the roles of SuSy in phloem. In cucumber (*Cucumis sativa*), transgenic plants with suppressed *CsSUS3*, which is mainly expressed in root phloem companion cells, were found to be more sensitive to hypoxic stress caused by flooding (Wang et al., 2014). In contrast, the Arabidopsis double mutant of the two phloem-specific *SUS* (*sus5 sus6*) exhibited no specific phenotype, even under hypoxic stress (Bieniawska et al., 2007; Barratt et al., 2009). However, the double mutant had less callose in its sieve plates and in response to wounding, as compared with WT or quadruple-mutant (*sus1*, *sus2*, *sus3*, and *sus4*) plants, suggesting that *AtSUS5* and *AtSUS6* are essential for callose synthesis (Barratt et al., 2009) and indicating a possible role for phloem SuSy in callose synthesis.

There is also sufficient evidence for the localization of SuSy to xylem tissues. Quantitative PCR analysis found the highest expression levels of *SUS2* in a poplar hybrid (*Populus simonii* × *Populus nigra*) in the xylem and phloem; those levels were 4-fold and 3-fold higher than the expression levels in the cambium, respectively (Wei et al., 2015). In transgenic tomato plants expressing GUS under the *SlSUS1* promoter, expression in the stem was observed mainly in the xylem (Goren et al., 2017). In the parasitic plant *Phelipanche ramosa*, *PrSUS1* transcript was found in the xylem of developing roots (Peron et al., 2012).

Sucrose synthase activity was found in immature metaxylem and the central vessel in the elongation zone of wheat seedlings following hypoxia (Albrecht and Mustroph, 2003). Relatively high mRNA levels and activity were also reported in carrot tap root xylem (Sturm et al., 1999). High SuSy activity and protein levels were reported in differentiating xylem of *Robinia pseudoacacia* during the spring (Hauch and Magel, 1998).

Sucrose synthase activity in developing xylem vessels or fibers may be particularly important for the cellulose synthesis needed for the construction of thick secondary cell walls. Work with a cell culture of *Zinnia elegans* revealed that SuSy is highly enriched in differentiating tracheary elements near the plasma membrane, where secondary cell-wall thickening occurs (Salnikov et al., 2001). Overexpression of *SUS* in several plant species increased the thickness of xylem cell walls (Coleman et al., 2009; Wei et al., 2015), further supporting the proposed roles of SuSy in xylem development (also discussed in section “Roles of SuSy in Cellulose and Callose Metabolism”).

## Roles of SuSy in Cellulose and Callose Metabolism

Because Suc cleavage by SuSy yields UDP-G, which is a direct substrate for both cellulose (β1-4) and callose (β1-3) glucans, it is widely assumed that SuSy plays a role in the synthesis of both of these polysaccharides. There is sufficient supporting evidence for these proposed roles from the SuSy subcellular localization to cell walls and adjacent to plasma membranes. Immunolocalization of the cotton fiber SuSy revealed an arrangement pattern similar to that of cellulose microfibril deposition (Amor et al., 1995). It was later found that a cellulose synthase rosette-like structure, isolated from azuki beans, lacks cellulose-synthesis activity in the absence of another particle referred to as the catalytic unit. This catalytic unit was enriched with a 78 kDa active SuSy, further supporting the suggested role of SuSy in supplying UDP-G for cellulose synthesis (Fujii et al., 2010). Another immunolocalization study also demonstrated that cotton fiber SuSy is co-localized with callose, suggesting a dual role for SuSy in cellulose and callose synthesis (Salnikov et al., 2003).

Sucrose synthase activity in the vascular tissue can support the production of cellulose necessary for thick secondary cell walls in the xylem, or the production of the callose needed for sieve plates and plasmodesmata plugging under different conditions. Evidence for a role of SuSy in callose deposition was found in an Arabidopsis double mutant of phloem-specific SUS (*sus5 sus6*). That double mutant had less callose in its phloem plasmodesmata and in response to leaf wounding, as compared with WT or quadruple mutant (*sus1*, *sus2*, *sus3*, and *sus4*) plants (Barratt et al., 2009).

The role of SuSy in the synthesis of cellulose and callose has been thoroughly investigated in cotton, with cotton fibers serving as a model for these processes. The development of cotton fibers starts with the initiation and elongation of the epidermal cells, followed by secondary growth and maturation marked by massive cellulose production. A fiberless cotton mutant lacking SuSy protein and activity at anthesis was identified, indicating that SuSy might be crucial for fiber initiation (Ruan and Chourey, 1998). It was later shown that transgenic cotton plants with *SUS* suppression exhibit reduced fiber initiation and elongation (Ruan et al., 2003) and that SuSy may play a role in both the synthesis of cellulose for cell walls and the deposition of callose in the plasmodesmata, which is required for the turgor build-up necessary for cell elongation (Ruan, 2007). In the secondary growth phase of cotton fibers, cellulose synthesis can increase 100-fold relative to the elongation phase (Delmer, 1999) and this process probably involves SusC and SusA (Brill et al., 2011). It would be very interesting to see whether the proposed roles of the cell wall SuSy in cellulose and callose synthesis could be observed in transgenic cotton plants with SusC suppression or overexpression.

Different reports also support the roles of SuSy in cellulose synthesis in other plant species. Although overexpression of cotton *SUS* in tobacco plants did not affect cellulose content (Coleman et al., 2006), its overexpression in poplar trees did increase their cellulose content, as well as cell-wall thickness and wood density (Coleman et al., 2009). Similarly, overexpression of poplar xylem *SUS* in tobacco plants also resulted in increased cellulose content and xylem cell-wall thickness (Wei et al., 2015). In contrast, *SUS* suppression in the developing wood of hybrid aspen (*Populus tremula* L. × *tremuloides* Michx.) did not affect cellulose synthesis specifically, but instead reduced the total amount of carbon incorporated into wood cell walls (Gerber et al., 2014).

## Role of SuSy in Low-Oxygen Environments

Oxygen deficiency (hypoxia) and a complete absence of oxygen (anoxia) are forms of serious abiotic stress that often cause reduced plant growth and productivity. Low-oxygen stress in plants is often caused by flooding, but may also occur naturally in dense, bulky and inner organs and tissues or in very rapidly growing tissues in which oxygen consumption is high. Oxygen is the final acceptor in the mitochondrial electron transport chain and the absence of oxygen blocks electron transfer and cellular ATP production. Some of the plants’ responses to oxygen deficiency can occur very rapidly and may involve changes in the transcription and activity of metabolic enzymes (Fukao and Bailey-Serres, 2004).

One of the enzymes thought to be involved in plant responses to hypoxia is SuSy. Transcript levels of some *SUS* genes have been found to increase in response to low levels of oxygen in potato (Biemelt et al., 1999), maize (McCarty et al., 1986; Bailey-Serres et al., 1988; Zeng et al., 1998), rice (Ricard et al., 1991; Hirose et al., 2008), carrot (Sturm et al., 1999), Arabidopsis (Martin et al., 1993; Dejardin et al., 1999; Baud et al., 2004), wheat (Marana et al., 1990), beet (Hesse and Willmitzer, 1996; Klotz and Haagenson, 2008), pigeon pea (*Cajanus cajan*) (Kumutha et al., 2008) and pondweed (*Potamogeton distinctus* ) (Harada et al., 2004, 2005).

Oxygen deficiency has also been shown to increase SuSy protein levels in Arabidopsis roots (Bieniawska et al., 2007) and leaves (Dejardin et al., 1999) and in maize roots (Zeng et al., 1998). In beet, *SUS1* mRNA levels increased under anaerobic conditions, but there was no increase in SuSy1 protein (Klotz and Haagenson, 2008). Increased SuSy activity under low-oxygen conditions has been noted in many plants and is often seen in combination with reduced INV activity in rice seedlings (Guglielminetti et al., 1995), maize seedlings (Zeng et al., 1998, 1999), Arabidopsis roots (Bieniawska et al., 2007), wheat roots (Albrecht and Mustroph, 2003) and potato tubers (Biemelt et al., 1999).

The possible role of SuSy in metabolism under reduced-oxygen conditions is further supported by the findings of studies with *SUS* mutants and transgenic plants. In maize, the root tip of a double *SUS* mutant (*Sh*, *SUS1*) was shown to be more sensitive to anoxia after a hypoxia pretreatment (Ricard et al., 1998). Potato tubers of a *SUS* antisense transgenic line were more sensitive to hypoxia than control plants (Biemelt et al., 1999). A study of potato tubers of transgenic plants overexpressing INV or Suc pyrophosphorylase, which allows a way to bypass the degradation of Suc by SuSy, revealed a steeper reduction in oxygen levels inside the tubers, reduced starch synthesis and a lower ATP to ADP ratio, underscoring the importance of SuSy under low-oxygen conditions (Bologa et al., 2003). In Arabidopsis, a double-knockout mutant (*sus1* and *sus4*) was found to be more sensitive to flooding than the control (Bieniawska et al., 2007), but did not differ from the WT in its responses to hypoxia or anoxia (Santaniello et al., 2014). In cucumber, antisense suppression of *CsSUS3* led to increased sensitivity to hypoxic stress (Wang et al., 2014). SuSy activity has also been found to be correlated with rice coleoptile length under submerged conditions, further indicating the advantage of Suc metabolism that involves SuSy under anoxic conditions (Fukuda et al., 2008).

Overall, the data show that some SuSy isoforms may indeed play a vital role in metabolism under low-oxygen conditions. Suc breakdown by SuSy may be more energy efficient than Suc breakdown via INV, as it may save up to two ATP molecules for each Suc molecule converted into hexose monophosphates (Guglielminetti et al., 1995).

## Role of SuSy in the Shoot Apical Meristem

Sucrose synthase may play another, less studied role in the development of shoot apical meristem (SAM). The SAM receives Suc from the phloem and there is evidence that *SUS* are expressed in the SAM. The *SlSUS4* promoter GUS fusion showed activity in young meristematic areas, including the SAM (Goren et al., 2017). RNAseq data obtained by Park et al. (2012) indicate that out of the six tomato *SUS* genes, *SlSUS1*, *SlSUS3*, and *SlSUS4* transcripts are found in both meristems and in primordia at all development stages in different ratios (Goren et al., 2017). The *SlSUS4* transcript was shown to be present asymmetrically and localized to the primordia from very early stages of development using *in situ* hybridization with an *SlSUS4* antisense probe (Pien et al., 2001). *SlSUS4* transcript levels around the SAM also seemed to increase in response to Suc, Glc and brassinosteroid treatments and to decrease when Fru was applied (Pien et al., 2001). *In situ* hybridization also revealed the presence of *SUS* transcript in young maize leaf primordia, suggesting a role for SuSy in early leaf development (Hanggi and Fleming, 2001).

Functional studies also suggest that *SUS* genes play significant roles in the SAM and in early leaf development. Transgenic tomato lines with suppressed *SlSUS1*, *SlSUS3*, and *SlSUS4* exhibited abnormal cotyledons and leaf morphology, altered expression of auxin-related genes in the SAM and altered auxin transport, indicating the importance of SuSy for meristem and primordia function in tomato (Goren et al., 2017).

Other work involving transgenic plants that overexpress *SUS* genes has revealed altered growth rates that may suggest some possible effects of these genes on SAM function. Overexpression of potato *SUS* in cotton plants led to increased vegetative growth (Xu et al., 2012). Similarly, overexpression of aspen (*Popolus tremuloides*) *SUS* in Arabidopsis resulted in an increased growth rate and increased plant biomass, and also induced early flowering (Xu and Joshi, 2010). Overexpression of cotton *SUS* in tobacco also led to an increased growth rate and taller plants (Coleman et al., 2006). Although the mechanism by which *SUS* overexpression speeds up the growth rate is not clear, it is tempting to speculate that increased SuSy activity in the meristem may facilitate increased cell proliferation.

In another study, researchers examined transgenic tobacco plants overexpressing each of the six Arabidopsis *SUS* genes (*AtSUS1-AtSUS6*) and found that all of those lines grew more quickly and were taller and thicker than the WT plants (Nguyen et al., 2016). The transgenic plants overexpressing *AtSUS1* showed increased chlorophyll levels, as well as increased photosynthesis, TSS (total soluble sugars), starch, Suc and Fru, as well as increased enzymatic activity of SPS and SPP in leaves, indicating increased sugar production in the transgenic plants. In addition, all six lines had increased Suc and Fru levels in their shoot tips and had a high percentage of dichotomous branches (30–40%, as compared to none in the WT), indicating a developmental event in the SAM that divided it into two SAMs and caused the formation of two branches. The results also revealed increased expression of both *WUSCHEL* (*WUS*) and *CycD3*, leading to the increased proliferation of meristematic cells. Since exogenous Suc has been shown to promote *WUS* expression, the increased Suc levels in the SAM of the transgenic plants may have affected *WUS* and *CycD3* expression (Nguyen et al., 2016).

In addition to serving as energy resources and structural components, sugars such as Suc, Glc, and Fru may also act as signaling molecules to regulate developmental processes and responses to environmental changes (Sheen et al., 1999; Eveland and Jackson, 2012). These sugars have also been shown to rapidly affect the expression of many genes, even at concentrations as low as 1 mM (Kunz et al., 2014). *AtSUS1* expression was found to be regulated by Glc via a HXK-dependent pathway (Ciereszko and Kleczkowski, 2002), meaning that not only does *SUS* regulate Suc metabolism, but that its own expression is also regulated by sugars. The role of sugars as signaling molecules in the SAM is a subject of lively debate and it is not always easy to differentiate between their signaling function and their metabolic role. In work with Arabidopsis seedlings conducted by Pfeiffer et al. (2016), it was shown that both sucrose and light affect *WUS* expression in the SAM, although Suc is unable to release SAM dormancy in the dark. Those authors also found that a non-metabolizable Suc analog, palatinose, has no effect on *WUS* expression in the dark, possibly indicating that Suc *per se* does not act as a signaling molecule in the SAM during seedling establishment.

Other studies have found correlations between Suc treatments or levels and flowering, suggesting that Suc may play a signaling role in the development of SAM into flowers (reviewed by Cho et al., 2018). The Suc signal for flowering may be mediated by trehalose 6-phosphate (T6P). T6P is generated from UDP-G and G6P by trehalose phosphate synthase (TPS) and is thought to be a signaling molecule rather than a metabolic substrate, because it exists at very low levels (Lunn et al., 2006). T6P levels is an indicator of high Suc levels and expression of *TPS1* in the SAM of Arabidopsis plants induces early flowering, further supporting the proposed roles of Suc and T6P in SAM in flowering (Wahl et al., 2013).

Another reason to believe that Suc and SuSy may play some regulatory function rather than just a metabolic one comes from tomato plants in which the expression of three *SUS* genes was suppressed (Goren et al., 2017). These plants exhibited abnormal leaf development and irregular auxin patterning, suggesting that altered sugar signaling in the SAM or primordia, rather than lower sugar metabolism, is likely to be the cause of these developmental changes.

## Other Roles of SuSy

Sucrose synthase may also play other important roles, in addition to its role in Suc cleavage. The localization of SuSy to mitochondria and its possible interaction with a high voltage-dependent anion channel suggest that these SuSy may play a role in regulating solute fluxes between the mitochondria and the cytosol (Subbaiah et al., 2006).

Plant SuSy have also been found to play a role in mutualism with symbiotic organisms like *Rhizobium* bacteria. In pea, a *SUS* mutant (*rug4*) exhibited a 90% reduction in SuSy activity in its root nodules. Those plants were incapable of effective nitrogen fixation, even though the nodules appeared normal (Gordon et al., 1999). Although nitrogenase protein levels were normal, there was no nitrogenase activity. It was suggested that Susy activity might be essential for nitrogen fixation in root nodules, due to the low-oxygen environment in the nodules (Gordon et al., 1999). Interestingly, the mutation did not appear to affect the plant’s symbiotic relationship with arbuscular mycorrhizae (Yarnes and Sengupta-Gopalan, 2009).

Sucrose synthase may also play a role in metabolism under heat stress. A recent study found that a *SUS3* allele that is highly expressed during seed ripening may confer resistance to the chalky grain phenotype of brown rice caused by heat stress (Takehara et al., 2018). The expression of the *SUS3* gene was found to be higher in the resistant line under heat stress. Interestingly, transgenic plants of a commercial rice cultivar expressing *SUS3* showed a decreased percentage of chalky grains under heat stress only when both the promoter and the cDNA of the heat-tolerant allele were introduced, indicating not only the importance of the SUS3 protein, but also the response rate to heat stress in terms of gene expression (Takehara et al., 2018). Another potential heat-tolerant SuSy was purified from a heat-tolerant line of wheat (WH-1021). That SuSy exhibited optimum activity at 37°C and was stable at temperatures up to 50°C (Verma et al., 2018), unlike other SuSy proteins, whose stability decreases at temperatures above 30°C (Schmolzer et al., 2016).

In strawberry (*Fragaria ananassa*), SuSy may play an important role in fruit ripening. Strawberry fruits with RNAi suppression of *FaSUS1* by virus-induced gene-silencing exhibited delayed fruit ripening, maintained their firmness and exhibited delayed anthocyanin accumulation (Zhao et al., 2017).

## Summary and Avenues for Future Research

To summarize, plant SuSy activity has been shown to play important roles in plant sugar metabolism, primarily in sink tissues. Plant SuSy proteins are found primarily in the cytosol or adjacent to the plasma membrane, although some SuSy isoforms are found in cell walls, mitochondria or vacuoles, or are bound to actin. Plant SuSy enzymes have been shown to be involved in several different metabolic pathways, such as those for starch, callose and cellulose synthesis, and to play developmental and possibly signaling roles in sink carbohydrate flux, vascular tissues and meristem functioning.

Every Suc molecule must be cleaved by SuSy or INV before it can be metabolized. The reason for the need of two Suc-catabolizing enzymes (SuSy and INV) in plants remains unclear. One possibility is suggested by their differential subcellular locations. The SuSy in plasma membranes and cell walls and their production of UDP-G may be important for directing carbon toward cellulose or callose synthesis; whereas INV may direct carbon to other metabolic pathways. However, SuSy and INV also co-localize to the cytosol. SuSy activity is feedback-inhibited by its product, Fru, and its activity is also reversible. These features of SuSy may help to control the amount of Suc consumed in different organs, for example, in stems and petioles. Only controlled amounts of the transported Suc must be cleaved and metabolized to support the maintenance and development of vascular and other supporting tissues. It is likely that feedback inhibition of SuSy activity and substrate inhibition of fructokinase by Fru (Schaffer and Petreikov, 1997; Kanayama et al., 1998) work together to impose a double break that controls the amount of Suc cleavage (German et al., 2003). That is, in case of excess cleavage of Suc by SuSy, the increased fructose Fru inhibits fructokinase activity so that fructose accumulates and that accumulated Fru inhibits further cleavage of Suc by SuSy.

Although plant SuSy proteins have been the subject of intensive study, we are still faced with major gaps in our understanding of the functions of these enzymes. The main issues that need to be further explored are: (1) the regulation of *SUS* expression and intracellular localization of specific SuSy isozymes, (2) the co-evolution of SuSy and INV and the division of Suc cleavage between these two group of enzymes, (3) the differences between the SUS I, SUS II, and SUS III clades, and (4) the roles of SuSy in meristem and leaf development.

## Author Contributions

OS and DG jointly wrote the manuscript, and read and approved the final manuscript.

## Conflict of Interest Statement

The authors declare that the research was conducted in the absence of any commercial or financial relationships that could be construed as a potential conflict of interest.

## Abbreviations

At: *Arabidopsis thaliana*
BD: *Brachipodium distachion*
Be: *Bamboo emeiensis*
Cit: *Citrus sinensis*
Ga: *Gossypium arboreum*
Gh: *Gossypium hirsutum*
Gm: *Glycine max*
Hb: *Hevea brasiliensis*
HV: *Hordeum vulgare*
MA: *Musa acuminata*
Md: *Malus domestica*
Os: *Oryza sativa*
PAB: *Picea abies*
PME: *Pseudotsuga menziesii*
Pp: *Prunus persica*
PPI: *Pinus pinaster*
PSI: *Pinus sitchensis*
PSY: *Pinus sylvestris*
PTA: *Pinus taeda*
Ptr: *Populus trichocarpa*
SB: *Sorghum bicolor*
SI: *Setaria italica*
Sl: *Solanum lycopersicum*
Vv: *Vitis vinifera*
Zm: *Zea mays*.
