# Supplementary material for: An Overview of Sucrose Synthases in Plants
Source: Front Plant Sci. 2019 Feb 8;10:95. doi: 10.3389/fpls.2019.00095 (PMC6375876; doi:10.3389/fpls.2019.00095)
Supplement: Supplementary file 1 [file Table_1.docx]

>AtSUS1

MANAERMITRVHSQRERLNETLVSERNEVLALLSRVEAKGKGILQQNQIIAEFEALPEQTRKKLEGGPFFDLLKSTQEAIVLPPWVALAVRPRPGVWEYLRVNLHALVVEELQPAEFLHFKEELVDGVKNGNFTLELDFEPFNASIPRPTLHKYIGNGVDFLNRHLSAKLFHDKESLLPLLKFLRLHSHQGKNLMLSEKIQNLNTLQHTLRKAEEYLAELKSETLYEEFEAKFEEIGLERGWGDNAERVLDMIRLLLDLLEAPDPCTLETFLGRVPMVFNVVILSPHGYFAQDNVLGYPDTGGQVVYILDQVRALEIEMLQRIKQQGLNIKPRILILTRLLPDAVGTTCGERLERVYDSEYCDILRVPFRTEKGIVRKWISRFEVWPYLETYTEDAAVELSKELNGKPDLIIGNYSDGNLVASLLAHKLGVTQCTIAHALEKTKYPDSDIYWKKLDDKYHFSCQFTADIFAMNHTDFIITSTFQEIAGSKETVGQYESHTAFTLPGLYRVVHGIDVFDPKFNIVSPGADMSIYFPYTEEKRRLTKFHSEIEELLYSDVENKEHLCVLKDKKKPILFTMARLDRVKNLSGLVEWYGKNTRLRELANLVVVGGDRRKESKDNEEKAEMKKMYDLIEEYKLNGQFRWISSQMDRVRNGELYRYICDTKGAFVQPALYEAFGLTVVEAMTCGLPTFATCKGGPAEIIVHGKSGFHIDPYHGDQAADTLADFFTKCKEDPSHWDEISKGGLQRIEEKYTWQIYSQRLLTLTGVYGFWKHVSNLDRLEARRYLEMFYALKYRPLAQAVPLAQDD

>AtSUS2

MPTGRFETMREWVYDAISAQRNELLSLFSRYVAQGKGILQSHQLIDEFLKTVKVDGTLEDLNKSPFMKVLQSAEEAIVLPPFVALAIRPRPGVREYVRVNVYELSVDHLTVSEYLRFKEELVNGHANGDYLLELDFEPFNATLPRPTRSSSIGNGVQFLNRHLSSIMFRNKESMEPLLEFLRTHKHDGRPMMLNDRIQNIPILQGALARAEEFLSKLPLATPYSEFEFELQGMGFERGWGDTAQKVSEMVHLLLDILQAPDPSVLETFLGRIPMVFNVVILSPHGYFGQANVLGLPDTGGQVVYILDQVRALENEMLLRIQKQGLEVIPKILIVTRLLPEAKGTTCNQRLERVSGTEHAHILRIPFRTEKGILRKWISRFDVWPYLETFAEDASNEISAELQGVPNLIIGNYSDGNLVASLLASKLGVIQCNIAHALEKTKYPESDIYWRNHEDKYHFSSQFTADLIAMNNADFIITSTYQEIAGSKNNVGQYESHTAFTMPGLYRVVHGIDVFDPKFNIVSPGADMTIYFPYSDKERRLTALHESIEELLFSAEQNDEHVGLLSDQSKPIIFSMARLDRVKNLTGLVECYAKNSKLRELANLVIVGGYIDENQSRDREEMAEIQKMHSLIEQYDLHGEFRWIAAQMNRARNGELYRYIADTKGVFVQPAFYEAFGLTVVESMTCALPTFATCHGGPAEIIENGVSGFHIDPYHPDQVAATLVSFFETCNTNPNHWVKISEGGLKRIYERYTWKKYSERLLTLAGVYAFWKHVSKLERRETRRYLEMFYSLKFRDLANSIPLATDEN

>AtSUS3

MANPKLTRVLSTRDRVQDTLSAHRNELVALLSRYVDQGKGILQPHNLIDELESVIGDDETKKSLSDGPFGEILKSAMEAIVVPPFVALAVRPRPGVWEYVRVNVFELSVEQLTVSEYLRFKEELVDGPNSDPFCLELDFEPFNANVPRPSRSSSIGNGVQFLNRHLSSVMFRNKDCLEPLLDFLRVHKYKGHPLMLNDRIQSISRLQIQLSKAEDHISKLSQETPFSEFEYALQGMGFEKGWGDTAGRVLEMMHLLSDILQAPDPSSLEKFLGMVPMVFNVVILSPHGYFGQANVLGLPDTGGQVVYILDQVRALETEMLLRIKRQGLDISPSILIVTRLIPDAKGTTCNQRLERVSGTEHTHILRVPFRSEKGILRKWISRFDVWPYLENYAQDAASEIVGELQGVPDFIIGNYSDGNLVASLMAHRMGVTQCTIAHALEKTKYPDSDIYWKDFDNKYHFSCQFTADLIAMNNADFIITSTYQEIAGTKNTVGQYESHGAFTLPGLYRVVHGIDVFDPKFNIVSPGADMTIYFPYSEETRRLTALHGSIEEMLYSPDQTDEHVGTLSDRSKPILFSMARLDKVKNISGLVEMYSKNTKLRELVNLVVIAGNIDVNKSKDREEIVEIEKMHNLMKNYKLDGQFRWITAQTNRARNGELYRYIADTRGAFAQPAFYEAFGLTVVEAMTCGLPTFATCHGGPAEIIEHGLSGFHIDPYHPEQAGNIMADFFERCKEDPNHWKKVSDAGLQRIYERYTWKIYSERLMTLAGVYGFWKYVSKLERRETRRYLEMFYILKFRDLVKTVPSTADD

>AtSUS4

MANAERVITRVHSQRERLDATLVAQKNEVFALLSRVEAKGKGILQHHQIIAEFEAMPLETQKKLKGGAFFEFLRSAQEAIVLPPFVALAVRPRPGVWEYVRVNLHDLVVEELQASEYLQFKEELVDGIKNGNFTLELDFEPFNAAFPRPTLNKYIGDGVEFLNRHLSAKLFHDKESLHPLLKFLRLHSHEGKTLMLNNRIQNLNTLQHNLRKAEEYLMELKPETLYSEFEHKFQEIGLERGWGDTAERVLNMIRLLLDLLEAPDPCTLENFLGRIPMVFNVVILSPHGYFAQDNVLGYPDTGGQVVYILDQVRALETEMLQRIKQQGLNITPRILIITRLLPDAAGTTCGQRLEKVYGSQYCDILRVPFRTEKGIVRKWISRFEVWPYLETFTEDVAAEISKELQGKPDLIIGNYSDGNLVASLLAHKLGVTQCTIAHALEKTKYPDSDIYWKKLDEKYHFSCQFTADLIAMNHTDFIITSTFQEIAGSKDTVGQYESHRSFTLPGLYRVVHGIDVFDPKFNIVSPGADMSIYFAYTEEKRRLTAFHLEIEELLYSDVENEEHLCVLKDKKKPIIFTMARLDRVKNLSGLVEWYGKNTRLRELVNLVVVGGDRRKESQDNEEKAEMKKMYELIEEYKLNGQFRWISSQMNRVRNGELYRYICDTKGAFVQPALYEAFGLTVVEAMTCGLPTFATCNGGPAEIIVHGKSGFHIDPYHGDKAAESLADFFTKCKHDPSHWDQISLGGLERIQEKYTWQIYSQRLLTLTGVYGFWKHVSNLDRLESRRYLEMFYALKYRPLAQAVPLAHEE

>AtSUS5

MEMTSGSLGNGIPEAMGQNRGNIKRCLEKYIENGRRVMKLNELMDEMEIVINDVTQRRRVMEGDLGKILCFTQEAVVIPPNVAFAVRGTPGNWQYVKVNSSNLSVEALSSTQYLKLKEFLFDENWANDENALEVDFGALDFTLPWLSLSSSIGNGLSFVSSKLGGRLNDNPQSLVDYLLSLEHQGEKLMMNETLNTARKLEMSLILADVFLSELPKDTPFQAFELRFKECGFEKGWGESAGRVKETMRILSEILQAPDPQNIDRFFARVPRIFNVVIFSVHGYFGQTDVLGLPDTGGQVVYILDQVKALEDELLQRINSQGLNFKPQILVVTRLIPDAKKTKCNQELEPIFGTKYSNILRIPFVTENGILRRWVSRFDIYPYLERFTKDATTKILDILEGKPDLIIGNYTDGNLVASLMANKLGITQATIAHALEKTKYEDSDIKWKEFDPKYHFSSQFTADLISMNSADFIIASTYQEIAGSKERAGQYESHMSFTVPGLYRVVSGINVFDPRFNIAAPGADDSIYFPFTAQDRRFTKFYTSIDELLYSQSENDEHIGYLVDKKKPIIFSMARLDVVKNLTGLTEWYAKNKRLRDLVNLVIVGGFFDASKSKDREEISEIKKMHSLIEKYQLKGQFRWITAQTDRTRNGELYRSIADTRGAFVQPAHYEAFGLTVIEAMSCGLVTFATNQGGPAEIIVDGVSGFHIDPSNGEESSDKIADFFEKSGMDPDYWNMFSNEGLQRINECYTWKIYANKVINMGSTYSYWRHLNKDQKLAKQRYIHSFYNLQYRNLVKTIPILSDIPEPPPLPPKPLVKPSASKGSKRTQPRLSFRLFGA

>AtSUS6

MSSSSQAMLQKSDSIAEKMPDALKQSRYHMKRCFASFVGGGKKLMKREHLMNEIEKCIEDSRERSKILEGLFGYILTCTQEAAVVPPFVALAARPNPGFWEYVKVNSGDLTVDEITATDYLKLKESVFDESWSKDENALEIDFGAIDFTSPRLSLSSSIGKGADYISKFISSKLGGKSDKLEPLLNYLLRLNHHGENLMINDDLNTVAKLQKSLMLAVIVVSTYSKHTPYETFAQRLKEMGFEKGWGDTAERVKETMIILSEVLEAPDNGKLDLLFSRLPTVFNVVIFSVHGYFGQQDVLGLPDTGGQVVYILDQVRALEEELLIRINQQGLGFKPQILVVTRLIPEARGTKCDQELEAIEGTKHSHILRVPFVTNKGVLRQWVSRFDIYPYLERFTQDATSKILQRLDCKPDLIIGNYTDGNLVASLMATKLGVTQGTIAHALEKTKYEDSDAKWKELDPKYHFSCQFTADLIAMNVTDFIITSTYQEIAGSKDRPGQYESHTAFTMPGLCRVVSGIDVFDPKFNIAAPGADQSVYFPYTEKDKRFTKFHPSIQELLYNEKDNAEHMGYLADREKPIIFSMARLDTVKNITGLVEWYGKDKRLREMANLVVVAGFFDMSKSNDREEKAEIKKMHDLIEKYKLKGKFRWIAAQTDRYRNSELYRCIADTKGVFVQPALYEAFGLTVIEAMNCGLPTFATNQGGPAEIIVDGVSGFHIDPNNGDESVTKIGDFFSKCRSDGLYWDNISKGGLKRIYECYTWKIYAEKLLKMGSLYGFWRQVNEDQKKAKKRYIEMLYNLQFKQLTKKVTIPEDKPLPLRLASLRNLLPKKTTNLGAGSKQKEVTETEKTKQKSKDGQEQHDVKVGEREVREGLLAADASERVKKVLESSEEKQKLEKMKIAYGQQHSQGASPVRNLFWSVVVCLYICYILKQRFFGANSAQEY

>SlSUS1

MAERVLTRVHRLRERVDATLCAHRNEILLFLSRIESHGKGILKPHELLAEFDAIRQDDKDKLNEHAFEELLKSTQEAIVLPPWVALAIRLRPGVWEYVRVNVNALVVEELSVPEYLQFKEELVDGASNGNFVLELDFEPFTASFPKPTLTKSIGNGVEFLNRHLSAKMFHDKESMAPLLEFLRAHHYKGKTMMLNDRIHNSNTLQNVLRKAEEYLIMLPPETPFFEFEHKFQEIGLEKGWGDTAERVLEMVCMLLDLLEAPDSCTLEKFLGRIPMVFNVVILSPHGYFAQENVLGYPDTGGQVVYILDQVPALEREMLKRIKEQGLDIIPRILIVTRLLPDAVGTTCGQRLEKVYGTEHSHILRVPFGTEKGIVRKWISRFEVWPYMETFIEDVAKEISAELQAKPDLIIGNYSEGNLAASLLAHKLGVTQCTIAHALEKTKYPDSDIYWKKFDEKYHFSSQFTADLIAMNHTDFIITSTFQEIAGSKDTVGQYESHMAFTMPGLYRVVHGINVFDPKFNIVSPGADINLYFPYSESEKRLTAFHPEIDELLYSDVENDEHLCVLKDRTKPILFTMARLDRVKNLTGLVEWYAKNPRLRGLVNLVVVGGDRRKESKDLEEQAEMKKMYELIETHNLNGQFRWISSQMNRVRNGELYRYIADTKGAFVQPAFYEAFGLTVVEAMTCGLPTFATNHGGPAEIIVHGKSGFHIDPYHGEQAADLLADFFEKCKKEPSHWETISTGGLKRIQEKYTWQIYSERLLTLAAVYGFWKHVSKLDRLEIRRYLEMFYALKYRKMAEAVPLAAE

>SlSUS3

MAQRVLTRVHSLRERLDATLDAHRNEILLFLSRIESHGKGILKPHQLLAEFESIQKEDKDKLNDHAFEEVLKSTQEAIVLPPWVALAIRLRPGVWEYVRVNVNALSVEELTVPEFLQFKEELVNGTSSDNFVLELDFEPFTASFPKPTLTKSIGNGVEFLNRHLSAKMFHDKESMTPLLEFLRVHHYNGKSMMLNDRIQNLYTLQKVLRKAEEYLTTLSPETSYSSFEHKFQEIGLERGWGDTAERVLEMICMLLDLLEAPDSCTLEKFLSRIPMVFNVVIPSPHGYFAQENVLGYPDTGGQVVYILDQVPALEREMLKRIKEQGLDIKPRILIVTRLLPDAVGTTCGQRLEKVFGTEHSHILRVPFRTEKGIVRKWISRFEVWPYMETFIEDVGKEITAELQAKPDLIIGNYSEGNLAASLLAHKLGVTQCTIAHALEKTKYPDSDIYLNKFDEKYHFSAQFTADLIAMNHTDFIITSTFQEIAGSKDTVGQYESHMAFTMPGLYRVVHGIDVFDPKFNIVSPGADVNLYFPYSEKEKRLTTFHPEIEDLLFSDVENEEHLCVLKDRNKPIIFTMARLDRVKNLTGLVEWYAKNPRLRELVNLVVVGGDRRKESKDLEEQAEMKKMYELIKTHNLNGQFRWISSQMNRVRNGELYRYIADTRGAFVQPAFYEAFGLTVVEAMSCGLPTFATNQGGPAEIIVHGKSGFQIDPYHGEQAADLLAEFFEKCKVDPSHWEAISKGGLKRIQEKYTWQIYSDRLLTLAAVYGFWKHVSKLDRLEIRRYLEMFYALKFRKLAELVPLAVE

>SlSUS4

MSNPKLSRIPSMRERVEDTLSAHRNQLVALLSRYVAQGKGILQPHHLIDELNNAVCDDTACEKLKEGPFC

EILKSTQEAIVLPPFVAIAVRPRPGVWEYVRVNVYDLSVEQLTVPEYLRFKEELVDGEDHNHLFVLELDF

EPFNASVPRPSRSSSIGNGVQFLNRHLSSNMFRSNESLDPLLDFLRGHNHKGNVLMLNERIQRISRLESS

LNKADDYLSKLPPDTPYTDFEYALQEMGFEKGWGDTANRVLETMHLLSDILQAPDPSTLETFLGRLPMVF

NVVILSPHGYFGQANVLGLPDTGGQVVYILDQVRALEAEMLLRIKQQGLNFKPRILVVTRLIPDAKGTTC

NQRLERISGTEYSHILRVPFRTENGILHKWISRFDVWPYLEKFTEDVAGEMSAELQGVPDLIIGNYSDGN

LVASLLAYKMGITQCTIAHALEKTKYPDSDIYWKKFEEKYHFSCQFTADLLSMNHSDFIITSTYQEIAGT

KNTVGQYESHTAFTLPGLYRVVHGIDVFDPKFNIVSPGADMTIYFPYFDKEKRLTSLHPSIEKLLFDPEQ

NEVHIGSLNDQSKPIIFSMARLDRVKNITGLVECYAKNATLRELANLVVVAGYNDVKKSNDREEIAEIEK

MHALMKEHNLDGQFRWISAQMNRARNGELYRYIADKRGIFVQPAYYEAFGLTVVEAMTCGLPTFATCHGG

PMEIIQDGVSGYHIDPYHPNKAAELMVEFFQRCEQNPTHWENISASGLQRILDRYTWKIYSERLMTLAGV

YGFWKLVSKLERRETRRYLEMFYILKFRELVKSVPLAVDEKQ

>SlSUS5

MAASGLSIKERLEEAILARPDEISALKSRIESEGKGVMKPLDLLNHLISVNSKKNGVNVGNSALVEILSY

SQEAIVVPPQLALAVRPRPGVWEYLSLNLKQQKVAELTIPEYLQLKENVFDESGNILEMDFEPFTTVTPT

KTLSDSIGNGLEFLNRHIASTMFHDKEIAKCLLDFLRQHNYKGKSLMVKESIQSLESFQFVLKKAEEYLC

TLSSETPYSDFESKFEEIGLERGWGNTAERVQETIRHLLHLLEAPNASSLENFLGRIPLVFNVVILTPHG

YFAQENVLGYPDTGGQVVYILDQVPAMETEMLLRLKHQGLDDIVPRILVVTRLLPDAVGTTCGERMEKVY

GAEHSHIIRVPFRTEKGMLRKWISRFEVWPYMETFTEDVAEELVKELQAKPDLIIGNYSEGNLAASLLAK

KFGATQCTIAHALEKTKYPNSDLYWKKFDDKYHFSSQFTADLYAMNHTDFIITSTFQEIAGSKNTVGQYE

SHTAFTMPGLYRVVHGIDSFDPKFNIVSPGADMSIYFPYTEKEKRLTKFHPEIEELLYSPVENKEHLCVL

KDRSKPILFTMARLDRVKNLTGLVEWYAKNARLRELVNLVVVGGDRRKESKDLEEQAEMKKMYDLIETYN

LNGQFRWISSQMNRVRNGELYRYIADTRGAFVQPAFYEAFGLTVVESMTCGLPTFATCNGGPFEIIVHGK

SGFHIDPNQGDKATDLLVKFFEKSKEDPSYWENISKGGLQRIIEKYTWQIYSQKVMTLSGIYGFWKFATK

NDKVASAKKRYLEMFYELMFKKSAEKVPLAIDE

>SlSUS6

MATTPVADSMPDALKQSRYHMKRCFARFIATGSRLMKLKYLMEDIENTIEDKAERTKVLEGSLGQTLSST

QEAAVVPPYVAFAVRHNPGCWDYVKVNADNLSVEAISPKEYLKFKEMIFDEEWAKDDNALEVDFGAFDYS

NPRLALSSSVGNGLNFVSKVMSSKLGGKPEEAQPLLDYLLALNHQGENLMINENLNSVSKLQAALIVAEV

FVSSFSKDTPYKNFEHKLKEWGFEKGWGDSAGRVRETMRLASEILQAPDPINMESFFSRLPTTFNIVIFS

IHGYFGQADVLGLPDTGGQVVYILDQVRALEEEMLSRIKQQGLNMKPKILVVTRLIPDARGTTCNQELEP

ILNSSHSHILRIPFRTEKGVLRQWVSRFDIYPYLENYAKDATVKILELMEGKPDLIIGNYTDGNLVASLL

ANKLGVTQGTIAHALEKTKYEDSDVKLKEFDPKYHFSCQFTADLLAMNAADFIITSTYQEIAGSETRPGQ

YESHTAFTMPGLYRVVSGINVFDPKFNIASPGAEQSAYFPFTERKKRFVKFGPAIEELLYSKEENNEHIG

FLADRRRPIIFSMARFDSVKNLTGLTEWFGKNKKLQNLVNLVIVGGFFDPSKSKDREEAAEIKKMHELIE

KYNLKGQMRWIAAQTDKYRNSELYRTIADTKGAFVQPALYEAFGLTVIEAMNCGLPTFATNQGGPAEIIV

DGVSGFHIDPYNGDESSNKIADFFEKCQVDSIYWNRISEGGLKRIEECYTWKIYANKVLNMGSIYGFWRR

FNVGQKQAKQRYFEMFYNPLFRKLANNVPIPYEEPLPVAPLDTIPSQEQKLPVPVPTAVAELPTLPTIAF

QRTEQKEEEKQVDTTTTSTTEIAKQATHWICLCVSASIIVYAMVKLYRIVE

>SlSUS7

MATTPALKRSESIADSMPEALRQSRYHMKRCFAKYIEQGKRMMKLHNLMDELEKVIDDPAERNHVLEGLL

GYILCTTMEAAVVPPYIAFATRQNPGFWEYVKVNANDLSVDGITATEYLKFKEMIVDESWAKDEYALEID

FGAVDFSTPRLTLSSSIGNGLSYVSKFLTSKLNASSMSAQCLVDYLLTLNHQGDKLMINETLSTVAKLQA

ALVVAEASISSLPTDTPYQSFELRFKEWGFEKGWGDTAERVRDTMRTLSEVLQAPDPSNIEKFFGRVPTV

FNIVLFSVHGYFGQADVLGLPDTGGQVVYVLDQVVAFEEELLQRIKQQGLNVKPQILVLTRLIPDAKGTK

CNQELEPINNTKHSHILRVPFRTEKGVLNQWVSRFDIYPYLERYTQDASDKIIELMEGKPDLIIGNYTDG

NLVASLMARKLGITLGTIAHALEKTKYEDSDIKLKELDPKYHFSCQFTADLIAMNSADFVITSTYQEIAG

SKDRPGQYESHSAFTLPGLYRVASGINVFDPKFNIAAPGADQSVYFPYTEKQKRLTDFRPAIEKLLFSKV

DNDEHIGYLEDRTKPILFTMARLDTVKNTTGLTEWFGKNKKLRSLVNLVVVGGSFDPTKSKDREEAAEIK

KMHVLIEKYQLKGQIRWIAAQTDRYRNSELYRTIADSKGAFVQPALYEAFGLTVIEAMNCGLPTFATSQG

GPAEIIVDGISGFHIDPNNGDESSNKIANFFQKCKEDPEHWNRISAQGLKRIYECYTWKIYANKVLNMGS

IYTFWRTLYKDQKQAKQRYIDTFYNLEFRNLIKDVPIKIDEKTEGPKERPERVKVKPQLSQRRSQSRLQK

LFGSSNSQS

>PsSUS1

MATDRLTRVHSLRERLDETLTANRNEILALLSRIEAKGKGILQHHQVIAEFEEIPEENRQKLTDGAFGEV

LRSTQEAIVLPPWVALAVRPRPGVWEYLRVNVHALVVENLQPAEFLKFKEELVDGSANGNFVLELDFEPF

TASFPRPTLNKSIGNGVQFLNRHLSAKLFHDKESLHPLLEFLRLHSYKGKTLMLNDRIQNPDSLQHVLRK

AEEYLGTVAPDTPYSEFEHRFQEIGLERGWGDTAERVLESIQLLLDLLEAPDPCTLETFLDRIPMVFNVV

ILSPHGYFAQDDVLGYPDTGGQVVYILDQVRALESEMLNRIKKQGLDIVPRILIITRLLPDAVGTTCGQR

LEKVYGTEHCHILRVPFRDQKGIVRKWISRFEVWPYLETYTEDVAHELAKELQGKPDLIVGNYSDGNIVA

SLLAHKLGVTQCTIAHALEKTKYPESDIYWKKFEEKYHFSCQFTADLFAMNHTDFIITSTFQEIAGSKDT

VGQYESHTAFTLPGLYRVVHGIDVFDPKFNIVSPGADQTIYFPYTETSRRLTSFYPEIEELLYSTVENEE

HICVLKDRSKPIIFTMARLDRVKNITGLVEWYGKNAKLRELVNLVVVAGDRRKESKDLEEKAEMKKMYEH

IETYKLNGQFRWISSQMNRVRNGELYRVICDTKGAFVQPAVYEAFGLTVVEAMATGLPTFATLNGGPAEI

IVHGKSGFHIDPYHGDRAADLLVEFFEKVKTDPSHWDKISQGGLQRIEEKYTWQIYSQRLLTLTGVYGFW

KHVSNLDRLESRRYLEMFYALKYRKLAESVPLAVEE

>PsSUS2

MSTHPKFTRVPSIRDRVQDTLSAHRNELISLLSRYVAQGKGILQPHNLIDELDNILGEDHATLDLKNGPF

GQIINSAQEAIVLPPFVAIAVRPRPGVWEYVRVNVFELSVEQLSVSEYLSFKEELVEGKSNDNIILELDL

EPFNASFPRPTRSSSIGNGVQFLNRHLSSNMFRNKDCLEPLLDFLRVHTYKGHALMLNDRIQSISKLQSA

LVKAEDHLSKLAPDTLYSEFEYELQGTGFERGWGDTAARVLEMMHLLLDILQAPDPSTLETFLGRVPMVF

NVVILSPHGFFGQANVLGLPDTGGQVVYILDQVRALESEMLVRIKKQGLDFTPRILIVTRLIPDAKGTTC

NQRLERVSGTEYTHILRVPFRSEKGILRKWISRFDVWPFLETFAEDVASEIAAELQCYPDFIIGNYSDGN

LVASLLAYKMGVTQCTIAHALEKTKYPDSDIYWKKFEDKYHFSCQFTADLIAMNNADFIITSTYQEIAGT

KNTIGQYESHTAFTLPGLYRVVHGIDVFDPKFNIVSPGADMTIYFPYSDKEKRLTALHSSIEKLLYGTEQ

TDEYIGSLTDRSKPIIFSMARLDRVKNITGLVESYAKNSKLRELVNLVVVAGYIDVKKSSDREEIEEIEK

MHDLMKQYNLNGEFRWITAQTNRARNGELYRYIADTKGAFVQPAFYEAFGLTVVEAMTCGLPTFATNHGG

PAEIIEHGVSGFHIDPYHPDQASELLVDFFQRCKEDPNHWNKVSDGGLQRIYERYTWKIYSERLMTLAGV

YSFWKYVSKLERRETRRYLEMFYILKFRDLANSVPIAKG

>PsSUS3

MASLTHSTSLRQRFDETLTAHRNEILSLLSRIEAKGKGILQHHQIIAEFEEIPEENRQKLVNGVFGEVLR

STQEAIVLVPFVALAVRPRPGVWEYLRVDVHGLVVDELSAAEYLKFKEELVEGSSNENFVLELDFEPFNA

SIPKPTQNKSIGNGVEFLNRHLSAKLFHGKESLQPLLEFLRLHNHNGKTIMVNDRIQNLNSLQHVLRKAE

DYLIKIAPETPYSEFEHKFQEIGLERGWGDTAERVVETIQLLLDLLDGPDPGTLETFLGRIPMVFNVVIL

SPHGYFAQDNVLGYPDTGGQIVYILDQVRALEEEMLKRIKQQGLDITPRILIITRLLPDAVGTTCGQRLE

KVYNTEHCHILRVPFRTEKGIVRKWISRFEVWPYLETFSEDVANELAKELQGKPDLIVGNYSDGNIVASL

LAHKLGVTQCTIAHALEKTKYPESDIYWKKFDDKYHFSSQFTADLFAMNHTDFIITSTFQEIAGSKDTVG

QYESHTAFTLPGLYRVVHGIDVFDPKFNIVSPGADLSIYFPYTETERRLTSFHPDIEELLYSTVENEEHI

CVLKDRSKPIIFTMARLDRVKNITGLVECYGKNARLRELVNLVVVAGDRRKESKDLEEIAEMKKMYGLIE

TYKLNGQFRWISAQMDRIRNGELYRVICDTKGAFVQPAIYEAFGLTVIEAMSCGLPTFATCNGGPAEIIV

HGKSGYHIDPYHGDRAAETLVEFFEKSKADPTYWDKISHGGLKRIHEKYTWQIYSDRLLTLTGVYGFWKH

VTNLERRESKRYLEMFYALKYSKLAESVPLAVEE

>PsSUS4

MATDRLTRVHSLRERLDETLTANRNEILALLSRIEAKGKGILQHHQVIAEFEEIPEENRQKLTDGAFGEVLRSTQEAIVLPPWVALAVRPRPGVWEYLRVNVHALVVENLQPAEFLKFKEELVDGSANGNFVLELDFEPFTASFPRPTLNKSIGNGVQFLNRHLSAKLFHDKESLHPLLEFLRLHSYKGKTLMLNDRIQNPDSLQHVLRKAEEYLGTVAPDTPYSEFEHRFQEIGLERGWGDTAERVLESIQLLLDLLEAPDPCTLETFLDRIPMVFNVVILSPHGYFAQDDVLGYPDTGGQVVYILDQVRALESEMLNRIKKQGLDIVPRILIITRLLPDAVGTTCGQRLEKVYGTEHCHILRVPFRDQKGIVRKWISRFEVWPYLETYTEDVAHELAKELQGKPDLIVGNYSDGNIVASLLAHKLGVTQCTIAHALEKTKYPESDIYWKKFEEKYHFSCQFTADLFAMNHTDFIITSTFQEIAGSKDTVGQYESHTAFTLPGLYRVVHGIDVFDPKFNIVSPGADQTIYFPYTETSRRLTSFYPEIEKLLYSTGGNEEHICVLKDRNKPIIFTMARLDRVKNITGLVEWYGKNAKLRELVNLVVVAGDRRKESKDLEEKAEMKKMYEHIETYKLNGQFRWISSQMNRVRNGELYRVICDTKGAFVQPAVYEAFGLTVVEAMATGLPTFATLNGGPAEIIVHGKSGFHIDPYHGDRAADLLVEFFEKVKTDPSHWDKISQGGLQRIEEKYTWQIYSQRLLTLTGVYGFWKHVSNLDRLESRRYLEMFYALKYRKLAESVPLAVEE

>GaSUS1

MAERALTRVHSLRERLDSTLTAHRNEILALLSRIEGKGKGILLHHQIILEFEAIPEENRKKLADGAFFEI

LKASQEAIVLPPWVALAVRPRPGVWEYIRVNVHALVVEELTVAEYLRFKEELVDGSSNANFVLELDFEPF

NASFPRPTLSKSIGNGVEFLNRHLSAKLFHDKESMHPLLEFLKVHCHKGKNMMLNDRIQNLNSLQHVLRK

AEEYLVALPAETPYAEFEHKFQEIGLERGWGDTAERVLEMIQLLLDLLEAPDPCTLEKFLGRIPMVFNVV

ILTPHGYFAQDNVLGYPDTGGQVVYILDQVRALENEMLNRIKQQGLNITPRILIITRLLPDAVGTTCGQR

LEKVYGTEYSDILRIPFRTEKGIVRRWISRFEVWPYLETYTEDVAHEISKELQGKPDLIIGNYSDGNIVA

SLLAHKLGVTQCTIAHALEKTKYPDSDIYWKKLEDKYHFSCQFTADLFAMNHTDFIITSTFQEIAGSKDT

VGQYESHTAFTLPGLYRVVHGIDVFDPKFNIVSPGADMSIYYPYTEEKKRLKHFHSEIEELLYSKVENEE

HWCVLNDRNKPILFTMARLDRVKNLTGLVEWYGKNAKLRELVNLVVVGGDRRKESKDLEEKAEMKKMFEL

IEKYKLNGQFRWISSQMNRVRNGELYRYICDTKGAFVQPALYEAFGLTVVEAMTCGLPTFATCNGGPAEI

IVHGKSGFNIDPYHGDQAAEILVDFFEKCKTDPSYWTKISEGGLKRIEEKYTWKIYSERLLTLTGVYGFW

KHVSNLDRLESRRYLEMFYALKYRKLAESVPLAVEE

>GaSUS2

MAERFDETLTSHRNEILPFLLRIEGKGKGILQHHQIALLIEDNRKKLADGAFYEILRAIQEATVSPPWVA

LAIRPRPGVWQYIKVNVHTLVVEDLTVSKYLHFKEQLVDGSANGNFVLELDFEPFNASFPRPTLSNAIGN

GAEFLNRHLSATLFHDDNENMHPLLEFLKLHCLPRLRMPDLNMMLLNDKIQNLNALRHVLRKAEEYLDTL

PSEILYAEFKHEFREIGLEPGWGDTAEHVLEMIRILSDLLEAPNPYNLEKFLGRVPMVFNVVILSPHGYF

AQDNVLGYPDTGGQVVYILDQVRALENEMIHRIKQQGLDITPRILIITRLLPDAVGTTCSERVEKVHGTE

YSDILRVPFRTENGIVRQWISRFEVWPYLETYTEDVANEITKELRGKPDLIIGNYSDGNIVASLLAHKLG

VTQCTIAHALEKTKYPNSDLYWKELEDKYHFSCQFTADLIAMNHTDFIITSTFQEIAGSKDSVGQYESHA

AFTLPGLYRVVHGIDVFDPKFNIVSPGADMSTFFPYTNEKQRLKHFHPEIEDLLYGKVENEEYICVLNDR

NKPILFTMARLDRVKNLTGLVEWYGKNPKLRKLVNLVVVAGDRRKESKDLEEKAEMKKMFELIEKYKLNG

QFRWISSQMNRIRNGELYRYVCDTKGAFVQPALYEAFGLTVVEAMTCGLPTFATCNGGPAEIIVHGKSGF

NIDPYQGDKAAEIIVGFFEKCKKDPSHWNEISNGGLKRIQEKYTWKIYSERLLTLTGVYSFWKHVSKLDR

RKSRRYLEMFYALNYRKLVESVPLTGEE

>GaSUS3

MADRVITRVHSLRERLDETLLAHRNEILALLSRIEGKGKGILQHHQIILEFEAIPEENRKKLANGAFFEV

LKASQEAIVLPPWVALAVRPRPGVWEYIRVNVHALVVEELTVAEYLHFKEELVDGSSNGNFVLELDFEPF

NSSFPRPTLSKSIGNGVEFLNRHLSAKLFHDKESMHPLLEFLRVHCHKGKNMMLNDRIQNLNALQHVLRK

AEEYLGTLPPETPCAEFEHRFQEIGLERGWGDTAERVLEMIQLLLDLLEAPDPCTLEKFLGRIPMVFNVV

ILTPHGYFAQDNVLGYPDTGGQVVYILDQVRALENEMLLRIKQQGLNITPRILIISRLLPDAVGTTCGQR

LEKVYGTEHSDILRVPFRTEKGIVRKWISRFEVWPYLETYTEDVAHEISKELQGKPDLIIGNYSDGNIVA

SLLAHKLGVTQCTIAHALEKTKYPDSDIYWKKLEDKYHFSCQFTADLFAMNHTDFIITSTFQEIAGSKDT

VGQYESHTAFTLPGLYRVVHGIDVFDPKFNIVSPGADMEIYFPYTEEKRRLKHFHPEIEDLLYSKVENEE

HLCVLNDRNKPILFTMARLDRVKNLTGLVEWYGKNAKLRELANLVVVGGDRRKESKDLEEKAEMKKMFEL

IDKYNLNGQFRWISSQMNRIRNGELYRYICDTKGAFVQPALYEAFGLTVVEAMTCGLPTFATCNGGPAEI

IVHGKSGFNIDPYHGDQAADILVDFFEKCKKDPSHWDKISQGGLKRIEEKYTWKIYSERLLTLTGVYGFW

KHVSNLERRESRRYLEMFYALKYRKLAESVPLAEE

>GaSUS4

MAERVITRVHSLRERLDDTLIAHRNEVLALLTRIEGKGKGILQHHQIILEFEAIPEETRKKLADGAFSEI

LRASQEAIVLPPWVALAVRPRPGVWEYIKVNVHALVVEELTVAEYLHFKEELVDGSANGNFVLELDFEPF

NASFPRSTLSKSIGNGVEFLNRHLSAKLFHDKESMHPLLEFLKVHCHKGKNMMLNDRIQNLNSLQYVLRK

AEEYLGTLPAETPYTELEHKFQEIGLERGWGDTAGRVLEMIQLLLDLLEAPDPCTLEKFLGRVPMVFNVV

ILTPHGYFAQDNVLGYPDTGGQVVYILDQVRALENEMLLRIKQQGLNITPRILIITRLLPDAVGTTCGQR

VEKVYGTEYSDILRVPFRTEKGIVRRWISRFEVWPYLETYTEDVAHEISKELQGKPDLIIGNYSDGNIVA

SLLAHKLGVTQCTIAHALEKTKYPDSDIYWKKLEDKYHFSCQFTADLIAMNHTDFIITSTFQEIAGSKDT

VGQYESHTAFTLPGLYRVVHGIDVFDPKFNIVSPGADMSIYFPYTEKKRRLKHFHPEIEDLLYSKVENEE

HLCVLNDRNKPILFTMARLDRVKNLTGLVEWYGKNAKLRELVNLVVVGGDRRKESKDLEEKAEMKKMFEL

IKTYKLNGQFRWISSQMNRVRNGELYRYICDTKGAFVQPALYEAFGLTVVEAMTCGLPTFATCKGGPAEI

IVHGKSGFNIDPYHGDQAAEILVDFFDKCKKEPSHWNDISEGGLKRIQEKYTWQIYSERLLTLTGVYGFW

KHVSNLDRRESRRYLEMFYALKYRKLAESVPLAEEE

>GaSUS5

MASISVCERLGESLATHPQQAKSILSRIESLGKGIHKSQKLLSVLDKEAGNQALDGMVVEVLRSTQEAVV

SSPLVALAIRSAPGVWEYIAVEVQKLFVEEMPVAEYLRLKEELVDGSSNGEFMLELDFGAFNNSVPRPSL

SKSIGNGMDFLNRHLSAKLFQDKENLSLLLEFLQIHCQKGKGMLLNDRIQDVNSLQHALRKAEEYLTPLT

SDTPYSVFEKRFREIGLEKGWGDNAEHVLEMIHLLLDLLQAPDPVALESFLGTIPLVANVVIMTPHGYFA

QDNVLGYPDTGGQVVYILDQVRALEEELLHRFKLQGLDITPRILVITRLLPDAVGTTCGQRLEKVYGTKY

SDILRVPFRTEKGIVRPWISRFKVWPYLETYTKDVAAEVTKEFQGKPDLIVGNYSDGNIVASLLALKFDV

TQCTIAHALEKTKYPDSDINWKQLEDKYHFSCQFTADLIAMNHTDFIITSTFQEIAGSKDTLGQYESHIA

FTLPGLYRVVDGIDVFDPKFNIVSPGADMSIYFPYTEEKRRLKKFHPEIEELLYSPVENTEHLCVLKDRN

KPVLFTMARLDRVKNLTGLVEFYAKNSRLRELVNLVVVGGDRRKESKDLEEKAEMKKMYELIEKYKLNGQ

LRWISSQMNRVRNGELYRYICDTKGAFVQPAIYEAFGLTVVEAMTCGLPTFATCYGGPAEIIVHGKSGFN

IDPYNGDLAAETLANFFEKCKADPSYWDEISQGGLKRIQEKYTWQIYSEKLLTLTGVYGFSKHVAYQEQR

GRKRYIEMLHALMYNNRVKTVPLAVE

>GaSUS6

MANPKLGRSPSMRDRVEDTLSAHRNELVALLSRYVAQGKGILQPHTLIDELENVVGDDKAREKLSDGPFS

EVLKSAQEAIILPPYVAIAVRPRPGVWEYVRVNVHELSVEQLDVSEYLRFKEALADVGEDNHFVLELDFE

PFNASFPRPNRSSSIGNGVQFLNRHLSSNMFRNKDSLEPLLNFLRAHKYKGHALMLNDRIQSIPRLQAAL

AKAEDHLAKLSPDAPYSEFEYELQGMGFERGWGDTAAHVLETMHLLLDILQAPDPSILETFLGRVPMVFN

VVILSPHGYFGQANVLGLPDTGGQVVYILDQVRALENEMLLRIKRQGLDITPRILIVTRLIPDAKGTSCN

QRLERVSGTEHTHILRVPFRSEHGVLRKWISRFDVWPYLETYAEDVAREIAAELQGIPDFIIGNYSDGNL

VASLLAYKMGVTQCTIAHALEKTKYPDSDIYWKKFDEKYHFSCQFTADLIAMNNADFIITSTYQEIAGTK

NTVGQYESHTAFTLPGLYRVVHGIDVFDPKFNIVSPGADMCIYFPYSEKEKRLTALHGSIEELLFDPKQN

DEHIGTLSDRSKPLIFSMARLDRVKNMTGLVELYAKNNKLRELANLVVVAGYIDVKKSKDREEIAEIEKM

HDLMKEYKLDGQFRWIAAQTNRARNGELYRYIADSKGIFVQPAFYEAFGLTVVEAMTCGLPTFATLHGGP

AEIIEHGISGFHIDPYHLDQTAELLATFFERCKEDPSHWTKISDGGLKRIYERYTWKIYSERLMTLAGVY

GFWKYVSKLERRETRRYLEMFYILKFRELVKSVPLASDD

>GaSUS7

MTSTSTGKLSDSIADNIRNALKQSQSYMKRCFSKYMEKGKRILKAHELRDEFEKVMDDKNETLGTMFSSA

QEAVVTPPYVTFTVRPTPGCWEFVKVNSVDLSDVKQISSAEYLKLKETTADENWSKDENALEVDFEAFDF

SMPKLTLASSIGKGLNFVSKYITSKLSGSVDNAQPLVDYLLSLEYQGEKLMINEILNTAAKLQLALIVAE

VSLSDLPRDTPYQSIELRFKEWGFERGWGDTVERVHETIRSLSEVLQAPDPQNLEKLFSKLPTIFKVVIF

SPHGYFGQSDVLGLPDTGGQVVYILDQVRAMEEELVLKIKSQGLNIKPQILVVTRLIPDARGTKCNQEWE

PVIGTKYSQILRVPFKTETGILRRWVSRFDIYPYLETFAQDVTSKILDAMEGKPDLIIGNYTDGNLVSSL

VASKLGITQATIAHALEKTKYEDSDVKWKELDPKYHFSCQFIADTIAMNAADFIIASTYQEIAGSKERPG

QYESHAAFTLPGLCRVVSGINVYDPKFNIAAPGADQSVYFPYTETGKRFTSFHPAIEELLYSKVDNDEHI

GYLADRKKPIIFSMARLDTVKNLTGLTEWYGKNKRLRSLVNLVIVGAFFNPSKSKDREEVAEIKKMHALI

EKYQLKGQIRWIAAQTDRNRNGELYRCIADTKGAFVQPALYEAFGLTVIEAMNCGLPTFATNQGGPAEII

VDGVSGFHINPTNGDESSNKIADFFEKCKTNPAYWNQFSADGLKRINECYTWKIYANKVLNMGCMYRFWK

QLNKDQKQAKQRYIQAFYNLMFRNLVKNVPLASDETQQPDSKPAAKPQPTPRHV

>GhSUS-D

MAERALTRVHSLRERLDSTLTAHRNEILALLSRIEGKGKGILLHHQIILEFEAIPEENRKKLADGAFFEILKASQEAIVLPPWVALAVRPRPGVWEYIRVNVHALVVEELTVAEYLRFKEELVDGSSNANFVLELDFEPFNASFPRPTLSKSIGNGVEFLNRHLSAKLFHDKESMHPLLEFLKVHCHKGKNMMLNDRIQNLNSLQHVLRKAEEYLVALPAETSYADFEHKFQEIGLERGWGDTAERVLEMIQLLLDLLEAPDPCTLEKFLGRIPMVFNVVILTPHGYFAQDNVLGYPDTGGQVVYILDQVRALENEMLNRIKQQGLNITPRILIITRLLPDAVGTTCGQRLEKVYGTEYSDILRIPFRTEKGIVRRWISRFEVWPYLETYTEDVAHEISKELQGKPDLIIGNYSDGNIVASLLAHKLGVTQCTIAHALEKTKYPDSDIYWKKLEDKYHFSCQFTADLFAMNHTDFIITSTFQEIAGSKDTVGQYESHTAFTLPGLYRVVHGIDVFDPKFNIVSPGADMSIYYPYTEEKKRLKHFHSEIEQLLYSKVENEEHWCVLNDHNKPILFTMARLDRVKNLSGLVEWYGKNAKLRELVNLVVVGGDRRKESKDLEEKAEMKKMFELIEKYKLNGQFRWISSQMNRVRNGELYRYICDTKGAFVQPALYEALGLTVVEAMTCGLPTFATCNGGPAEIIVHGKSGFNIDPYHGDQAAEILVDFFEKCKTDSSYWTKISEGGLKRIEEKYTWKIYSERLLTLTGVYGFWKHVSNLDRLESRRYLEMFYALKYRKLAESVPLAVEE

>GhSUS-A

MAERALTRVHSLRERLDETLLAHRNEILALLSRIEGKGKGILQHHQIILEFEAIPEENRKKLANGAFFEVLKASQEAIVLPPWVALAVRPRPGVWEYIRVNVHALVVEELTVAEYLHFKEELVDGSSNGNFVLELDFEPFNSSFPRPTLSKSIGNGVEFLNRHLSAKLFHDKESMHPLLEFLRVHCHKGKNMMLNDRIQNLNALQHVLRKAEEYLGTLPPETPCAEFEHRFQEIGLERGWGDTAERVLEMIQLLLDLLEATDPCTLEKFLGRIPMVFNVVILTPHGYFAQDNVLGYPDTGGQVVYILDQVRALENEMLLRIKQQGLNITPRILIITRLLPDAVGTTCGQRLEKVYGTEHSDILRVPFRTEKGIVRKWISRFEKVWPYLETYTEDVAHEISKELHGTPDLIIGNXSDGNIVASLLAHKLGVTQCTIAHALEKTKYPDSDIYWKKLEDKYHFSCQFTADLFAMNHTDFIITSTFQEIAGSKDTVGQYESHTAFTLPGLYRVVHGIDVFDPKFNIVSPGADMEIYFPYTEEKRRLKHFHPEIEDLLYTKVENEEHLCVLNDRNKPILFTMPRLDRVKNLTGLVEWCGKNPKLRELANLVVVGGDRRKESKDLEEKAEMKKMFELIDKYNLNGQFRWISSQMNRIRNVELYRYICDTKGAFVQPALYEAFGLTVVEAMTCGLPTFATCNGGPAEIIVHGKSGFNIDPYHGDQAADILVDFFEKCKKDPSHWDKISQGGLKRIEEKYTWKIYSERLLTLTGVYGFWKHVSNLERRESRRYLEMFYALKYRKLAESVPLAEE

>GhSUS-B

MAERVITRVHSLRERLDDTLIAHRNEVLALLTRIEGKGKGILQHHQIILEFEAIPEETRKKLADGAFSEILRASQEAIVLPPWVALAVRPRPGVWEYIKVNVHALVVEELTVAEYLHFKEELVDGSANGNFVLELDFEPFNASFPRSTLSKSIGNGVEFLNRHLSAKLFHDKESMHPLLEFLKVHCHKGKNMMLNDRIQNLNSLQYVLRKAEEYLGTLPAETPYTELEHKFQEIGLERGWGDTAGRVLEMIQLLLDLLEAPDPCTLEKFLGRVPMVFNVVILTPHGYFAQDNVLGYPDTGGQVVYILDQVRALENEMLLRIKQQGLNITPRILIITRLLPDAVGTTCGQRVEKVYGTEYSDILRVPFRTEKGIVRRWISRFVVWPYLETYTEDVAHEISKELQGKPDLIIGNYSDGNIVASLLAHKLGVTQCTIAHALEKTKYPDSDIYWKKLEDKYHFSCQFTADLIAMNHTDFIITSTFQEIAGSKDTVGQYESHTAFTLPGLYRVVHGIDVFDPKFNIVSPGADMSIYFPYTEEKRRLKHFHPEIEDLLYSKVENEEHLCVLNDRNKPILFTMARLDRVKNLTGLVEWYGKNAKLRELVNLVVVGGDRRKESEDLEEKAEMKKMFELIETYKLNGQFRWISSQMNRVRNGELYRYICDTRVAFVQPALYEAFGLTVVEAMTCGLPTFATCNGGPAEIIVHGKSGFNIDPYHGDQAAEILADFFDKCKKDPSHWNDISEGGLKRIQEKYTWQIYSERLLTLTGVYGFWKHVSNLDRRESRRYLEMFYALKYPKLAESVPLAEE

>GhSUS-C

MASISVCERLGESLATHPQQAKSILSRIESLGKGIHKSQKLLSVLDKEAGNQALDGMVVEVLRSTQEAVVSSPLVALAIRSAPGVWEYIAVEVQKLFVEEMPVAEYLRLKEELVDGSSNGEFMLELDFGAFNNSVPRPSLSKSIGNGMDFLNRHLSAKLFQDKENLNLLLEFLQIHCQKGKGMLLNDRIQDVNSLQHALRKAEEYLTPLSSDTPYSVFEKRFLGIGLEKGWGDNAEHVLEMIHLLLDLLQAPDPVALESFLGRIPLVANVVIMTPHGYFAQDNVLGYPDTGGQVVYILDQVRALEEELLHRFKLQGLDITPRILVITRLLPDAVGTTCGQRLEKVYGTKYSDILRVPFRTEKGIVRPWISRFKVWPYLETYTKDVAAEITKEFQGKPDLIVGNYSDGNIVASLLAHKFDVTQCTIAHALEKTKYPDSDINWKQLEDKYHFSCQFTADLIAMNHTDFIITSTFQEIAGSKDTLGQYESHIAFTLPGLYRVVDGIDVFDPKFNIVSPGADMSIYFPYTEEKRRLKKFHPEIEELLYSPVENTEHLCVLKDRNKPILFTMARLDRVKNLTGLVEFYAKNSRLRELVNLVVVGGDRRKESKDLEEKAEMKKMYELIEKYKLNGQFRWISSQMNRVRNGELYRYICDTKGAFVQPPIYEAFGLTVVEAMTCGLPTFATCYGGPAEIIVHGKSGFNIDPYNGDLAAETLANFFEKCKADPSYWDEISQGGLKRIQEKYTWQIYSEKLLTLTGVYGFSKHVAYQEQRGRKRYIEMLHAWMYNNRVKTVPLAVE

>MdSUS1.1

MAERALTRVQSLRERLDETLXTHRNEILALLSRIESKGKGFLQPHQLHAEFEAIPENNRQKLLDGAFGEV

LKHTQEAVVLPPWVAFAVRPRPGVWEYIRVNVHALVLEELRVAEYLQFKEELVDGSSNGNFVLELDFQPF

HASFPRPTLSKSIGKGVEFLNRHLSAKLFHDKESLHPLLEFLRVHCYEGRNMMLNNRIKNVNELERVLRK

AEDFLSSXVPGTPYKEFEPKXQXIGLERGWGDTAERVLEMIQLLLDLIEAPDPFTLEKFLGQIPMVFNVV

ILSPHGYFAQDHVLGFPDTGGQVVYILDQVRALESEMLKRIKQQGLDITPRIIILTRLLPDAVGTTCGDR

LEKVYGCEHSDILRVPFRDXKGXIVRRWISRFEVWPYLZXYAXDAIXELSKEMQGKPDLIIGNYSDGNIV

ASLMAHXLGVTQCTIAHALEKSKYPDSDLYWKKLDENYHFSCQFTADLIAMNHTDFIITSTFQEIAGSKD

TVGQYESHTAFTLPGLYRVVHGIDVFDPKFNIVSPGADXBVYFPXSEKEKRLTHFHPEIEELLYGQVENI

EXLCVLKDRNKPIIFTMARLDRVKNITGLVEWYGKNAKLRELVNLVVVAGDRRNESKDNEEKAELKKMYE

LIDTYKLNGQLRWISSQMNRVRNGELYRYICDTRGAFVQPAVYEAFGLTVIEAMTCGLPTFATCKGGPAE

IIVNGKSGYHIDPYHGDQAAEIXVDFFEKNKADPSHWDKISQGGLQRIYEKYTWQIYSERLLTLTGVYGF

WKDVSNLDRLESRRYLEMFYALKFRKLAASVPLAVEE

>MdSUS1.2

MAERALTRVQSLRERLDETLFTHRNEILALLSRIESKGKGFLQPHQLHAEFEAIPENNRQKLLDGAFGEV

LKHTQEAVVLPPWVAFAVRPRPGVWEYIRVNVHALVLEELRVAEYLQFKEELVDGSSNGNFVLELDFQPF

HASFPRPTLSKSIGKGVEFLNRHLSAKLFHDKESLHPLLEFLRVHCYEGRNMMLNNRIKNVNELERVLRK

AEDFLSSVVPGTPYKEFEPKMQVIGLERGWGDTAERVLEMIQLLLDLIEAPDPFTLEKFLGQIPMVFNVV

ILSPHGYFAQDHVLGFPDTGGQVVYILDQVRALESEMLKRIKQQGLDITPRIIILTRLLPDAVGTTCGDR

LEKVYGCEHSDILRVPFRDEKGAIVRRWISRFEVWPYLQTYAEDAIKELSKEMQGKPDLIIGNYSDGNIV

ASLMAHKLGVTQCTIAHALEKSKYPDSDLYWKKLDENYHFSCQFTADLIAMNHTDFIITSTFQEIAGSKD

TVGQYESHTAFTLPGLYRVVHGIDVFDPKFNIVSPGADKDVYFPYSEKEKRLTHFHPEIEELLYGQVENI

EQLCVLKDRNKPIIFTMARLDRVKNITGLVEWYGKNAKLRELVNLLVVAGDRRNESKDNEEKAELKKMYE

LIDTYKLNGQLRWISSQMNRVRNGELYRYICDTRGAFVQPAVYEAFGLTVIEAMTCGLPTFATCKGGPAE

IIVNGISGYHIDPYHGDQAAEILVDFFEKSKADPSHWDKISQGGLQRIYEKYTWQIYSERLLTLTGVYGF

WKDVSNLDRLECRRYLEMFYALKFRKLAASVPLAVE

>MdSUS1.3

MAERVLTRVQSLRERLDETLSTQRNDFLALLSRIESKGKGFLQPHQLLAEFEAIPENNRQKLLDGAFGEV

LKHTQEAIVLPPWVALAVRPRPGVWEYIRVNVHALALEELCVAEYLQFKEELVDGSSNGNFVLELDFQPF

NASFPRPTLSKSIGNGVEFLNRHLSAKLFHDKESLHPLLEFLRVHCYEGRNMMLNNRIKNVNELQRVLRK

AEDFLSSIVPRTPYKEFEPKLQAIGLERGWGDTAERVLEMIQLLLDLLEAPNPFTLEKFLGQIPMVFNVV

ILSPHGYFAQDHVLGFPDTGGQVVYILDQVRALESEMLKRIKQQGLDITPRIIILTRLLPDAVGTTCGDR

LEKVYGCEHSDILRVPFRDKKGAIVRRWISRFEVWPYLETYAGDAITELSKEMQGKPDLIIGNYSDGNIV

ASLMAHXLGVTQCTIAHALEKSKYPDSDLYWKKLDENYHFSCQFTADLIAMNHTDFIITSTFQEIAGSKD

TVGQYESHTAFTLPGLYRVVHGIDVFDPKFNIVSPGADMNVYFPFSEKEKRLTHFHPEIEELLYGQVENI

EHLCVLKDRNKPIIFTMARLDRVKNITGLVEWYGKNAKLRELVNLVVVAGDRRNESKDNEEKAELKKMYE

LIDTYKLNGQLRWISSQMNRVRNGELYRYICDTRGAFVQPAVYEAFGLTVIEAMTCGLPTFATCKGGPAE

IIVNGKSGYHIDPYHGDQAAEIXVDFFEKNKADPSHWDKISQGGLQRIYEKYTWQIYSERLLTLTGVYGF

WKDVSNLDRLESRRYLEMFYALKFRKLAASVPLAVEE

>MdSUS1.4

MAERVLTRVQSLRERLDETLSAHRNEILALLSRIESKGKGFLQPHQLLAEFEEIPEANRQKLLDGAFGEV

LKNTQEVIVLPPWVALAVRPRPGVWEYIRVNVHALVVEELRVAEYLQFKEELVDGSANGNFVLELDFEPF

NASFPRPTLSKSIGNGVEFLNRHLSAKLFHDKESLHPLLKFLRVHCYEGRNLMLNNKIKDVNELQHVLRK

AEDFLSAIAPETPYKDFEPKLQAIGLERGWGDTAGRVLEMIELLLDLLEAPDPCTLEKFLDQIPMVFNVV

ILSPHGYFAQDNVLGYPDTGGQVVYILDQVRALEAEMLKRVKQQGLDIIPRIIILTRLLPDAVGTTCGDR

LEKVYGTEHSDILRVPFRDEKGAIVRRWISRFEVWPYLETYAEDAIKELSKEMHGKPDLIIGNYSDGNIV

ASLMALKLGVTQCTIAHALEKSKYPDSDLYWKKLDDSYHFSCQFTADLIAMNHTDFIITSTYQEIAGSKE

TVGQYESHTAFTLPGLYRVVHGIDVFDPKFNIVSPGADMSVYFPYSEKEKRLTNFHPEIEELLYSQVENK

EHLCVLKDRNKPIIFTMARLDRVKNITGLVEWYGKNAKLRELVNLVVVAGDRRKESKDNEEKAEMKKMYE

LIDTYKLNGQFRWISSQMNRVRNGELYRYICDTKGAFVQPAVYEAFGLTVVEAMTCGLPTFATCKGGPAE

IIVHGKSGYHIDPYHGDQAAEILVDFFEKSKADPSHWDKISQGGLQRIYEKYTWQIYSQRLLTLTGVYGF

WKHVSNLDRLESRRYLEMFYALKYSKLAASVPLAVEE

>MdSUS2.1

MANRPKFTRALSLRERVEDTLSDHRNELVALLSRYLDQGKRILQPHDLIDQLDIVIGDDEAKRQLKTGPF

SEVLKSAQEAIILPPYVALAVRPRPGVWDYVRVNVYELSVEELTVSEYLRFKEELVDGESSDKYALELDF

EPFNAAFPRPTRSSSIGNGVQFLNRHLSSIMFRNKESLEPLLDFLKAHKYKGHPLMLNDRIQSVSKLQSA

LAKAEDHLSKLQPETPYSEFEYLFQGMGFERGWGDTAVHVLEMMHLLLDILQAPDPSILETFLGRIPMMF

NVVILSPHGYFGQANVLGLPDTGGQIVYILDQVRALEKEMLERIRLQGLDFTPRILIVTRLIPEAKGTTC

NQRLERISGTEHTHILRVPFRSEKGILRKWISRFDVWPYLETFAEDAAGEIIAELQGYPDFIIGNYSDGN

LVASLLAYKMGVTQCTIAHALEKTKYPDSDIYWKKFEEKYHFSTQFTADLIAMNNADFIITSTYQEIAGT

KDTVGQYESHSSYTLPGQYRVVHGINVFDPKFNIVSPGADMAIYFPYSEKQKRLTSLHGSLEELLYNPDQ

NDVHIGTLSDRSKPIIFSMARLDQVKNMTGLVECYAKCSKLRDLANLVIVAGYIDAKKSQDREEIAEIEK

MHNLMTEYKLDGQFRWISXQTNRVSNGELYRYIADTRGAFAQPAFYEAFGLTVVEAMTCGLPTFATVHGG

PAEIIEHGVSGFHIDPYHPEKAAALMADFFQRCKEDPSYWNTISDAGLQRIYEKYTWKIYSERLMTLAGV

YGFWKYVSKLERRETRRYLEMFYILKFRDLAKSVPEAIDDAH

>MdSUS2.3

MRNTIQDTLAAHRNELVSLLSRYVARGNGILQPHQMINELENVIMEDEGMQKLKDSSFSKVLQSAQEAIV

LAPFVAFALRPRPGVWEYVRVNVYELSVDHLSVAEFLRFKEELMDGECNDKYVLELDLEPFNASFPRPTR

SSSIGNGVQFLNRHLSSVMFRNKESLEPLLDFLRTHKHDGHAMMLNDRIQSIPRLQSALAKAEEYLSKFP

PTTSYSEFEFDLQGMGFERGWGDTAQRVSEMVHLLLEILQAPDPSTLENFLGRIPMVFNVVIVSPHGYFG

QANVLGLPDTGGQVVYILDQVRALESEMLLRIQNQGLDVIPKILIVTRLIPDAKGTTCNQRLERVSGTEY

THILRVPFRTENGILRKWISRFDVWPYLETFAEDASNEIAAELQGVPDLVIGNYSDGNLVATLLSYKLGI

TQCNIAHALEKTKYPDSDIFWKKHEDKYHFSSQFTADLIAMNNABFIITSTYQEIAGSKNNVGQYESHTA

FTLPGLYRVVHGIDVFDPKFNIVSPGADMCIYFPYSDKEKRLTALHGSIEELLYGAEQNDEHIGLLSDRS

KPIVFSMARLDRVKNLTGLVECYAKSAKLREMVNLVVVGGYMDVKNSRDREEXAEIEKMHDLIKKYNLXG

QFRWIAAQMNRARNGELYRYIADTKGVFVQPAFYEAFGLTVVEAMTCGLPTFATCHGGPAEIIXHGSSGF

HVDPYNPDQVAELLIDFFDXCQKXPGYWEKISQAGLKRIYERYTWKIYSERLLTLAGVYGFWKHVSKLER

RETRRYLEMFYILKYRNLVSNLIISFLVLXHAQNGTGXP

>MdSUS3.1

MASTSSALKRSDTIAETMPDALRESRFHMKKCFASFVGTGKRLIKPQHIMEELEKSIEDRHERSKVLEGL

LGYILSRTQEAAVVPPYVAFAVRPNPGFSEFVKVNADDLAVDGISATQYLKFKEMIFDESWANDENALEI

DFGAFDFSTPRMTLPSSIGNGLNFVLKLISSRLSTHASCSDYAKPLLDYLLPLNYHGENLMINESLDTVE

KLQTALIRAEVLVSTLPKTTPFPNFEQRFKVLGFEKGWGDTAERVGETMRLLSEVLQAPDSVKLESLFSR

LPNTFNIVIFSPHGYFGQSNVLGLPDTGGQVVYILDQVRALEEELLLRIKQQGLAVKPQILVVTRLIPDA

RGTKCNQELEAIIDTKHSHILRVPFRTEKGVLRQWVSRFDIYPYLETFALDATAKILRHMECKPDLIIGN

YSDGNLVASLIASKLGITQGTIAHALEKTKYEDSDAKWKEFDPKYHFSCQFTADIISMNCADFVITSTFQ

EIAGGKDRPGQYESHTAFTMPGLYRVVSGIDVFDPKFNIAAPGADQSVYFPYSEKQKRFTKFQPAIEELL

YKKEENDEHIGFLADQKKPIIFSMARLDTVKNLTGLVEWFGKNKRLRNLVNLVIVGGFFDPSKSKDREEI

AEIKKLHALVQEYQLSGQFRWIAAQTDRYRNGELYRCIADTKGSFVQPALYEAFGLTVIEAMNCGLPTFA

TNQGGPAEIIVDGVSGFHIDPNNGDESSNKIADFFEKCKTDGEYWKKMSAAGLQRINECYTWKIYANKVL

NMGSTYGFWRQLRDAQKLAKETYIHMFYNLLFRKLAKNVAVPSDGYEQPAPKAVTASVDQRAPAAVSKPP

QPDAAPTLAIPQLTPRERDEGGELGQPRSRSRARCPWNCCCVILGFLIILYYKIRNMYN

>MdSUS3.2

MASAAAIKGSESIADNMPDARRQSRYHMKRCFAKYXEKGRRIIKLHHLMSEMETVIDDKAERTQVLEGVL

GYILCSTQEAVVIPPHVVFSIRPNPGYWEFVKVSSEDLSIEGITVRDFLKYKETLYDENWSNDENALEVD

FRAIDFSTPHLTLSSSIGNGINYVTKFTTSKLAGKLENAQPLVDYLLSLNHQGEQLILNETLNTASKLQA

ALIVTDVHLSALPKDTPFQNFELRFKEWGFEKGWGDTAERTKETMGILAEVLQAPDPLIMDRFFSRLPTI

FNVVIFSPHGYFGQADVLGLPDTGGQVVYILDQVKALEEELLVRIKQQGLTVKPQILVVTRLIPEARGTK

CNQELEPINGTKYSNILRVPFRTEKGILRRWVSRFDIYPYLELFTQDATAKILDLMEGKPDLIIGNYTDG

NLVASLMANKLGITQATIAHALEKTKYEDSDINWKXLDPKYHFSCQFLADTISMNATDFVIASTYQEIAG

SKDRPGQYESHTAFTXPGLCRVVSGINVFDPKFNIAAPGADQSVYFPYAEKQKRLTSFHPAIEELLFSKE

DBNEHIGFLVDRKKPIIFSMARLDIVKNITGLVEWYGKNKRLXNLVNLVVVGGFFDPSKSKDREEIAEIK

KMHTLIEKYZLRGQIRWIAAQTDRNRNGELYRCIADTRGAFVQPALYEAFGLTVIEAMNCGLPTFATNQG

GPAEIIVDGISGFHIDPNNGDEASNKIADFFEKSKTDAAYWDRISKAGLQRIYECYTWKIYANKVLNMGS

TYTFWRQLNKEQKQAKQRYIQMFFSLQYRNLVKNVPIPSDETEQPVVPKPTAKPKTTLSTRRSQSRV

>MdSUS3.4

MASAAAVKRSDSIAETMPDALRQSRYHMKRCFAMYIEKGRRIMKLHHLMSEMETVIDDKAERTQVLGGVL

GYILCSTQEAVVIPPHVVFSIRPNPGYWEFVKVSSEDLSVEAITVRDFLKXKEALYDEKWSNDEHVLEVD

FRAIDFSTPXLTLSSSVGNGXXYVTKFTTSRLAGKLENAQPLXDYLLSLNHQXEQLILNETLNTASKLQA

ALIVTEVYLSDLPKDTPFQNFELSFKEWGFEKGWGDTAERTKETMKILLEVLQAPDPLNMDRFFSRLPTI

FNVVIFSPHGYFGQADVLGLPDTGGQVVYILDQVKAMEEELTLRIKQQGLTVKPQILVVTRLIPEARGTK

CNQELEPINGTKYSNILRVPFRTEKGILRRWVSRFDIYPYLELFTQDATAKILNLMEGKPDLIIGNYTDG

NLVASLMANKLGITQATIAHALEKTKYEDSDINWKELDPKYHFSCQFLADTISMNATDFVIASTYQEIAG

SKDRPGQYESHTAFTLPGLCRVVSGINVFDPKFNIAAPGADQSVYFPYSDKXKRLTSFYPAIEELLFSKE

DXSEHLGFLVDRKKPIIFSMARLDIVKNITGLVEWYGKNKRLRNLVNLVVVGGFFDPSKSKDREEIAEIK

KMHTLIEKYQLRGQIRWIAAQTDRNRNGELYRCIADTRGAFVQPALYEAFGLTVIEAMNCGLPTFATNQG

GPAEIIXDGVSGFHIDPNNGDEASXKXADFFENSKTDXAYWDRFSKAGLQRIYECYTWKIYANKVLNMGS

TYTFWRQLNKEQKQAKQRYIQMFFNLQYRNLVKNVPVPXDEPEQPQTTSRHVNLSNETL

>PtrSUS1

MAERALTRVHSIRERVDETLKAHRNEIVALLTRIEGKGKGILQHHQIVAEFEAIPEDTRKTLAGGAFAEV

LRSTQEAIVVPPWIALALRPRPGVWEYIRLNVQALVVEELRVAEYLHFKEELVDGGCNGNFVLELDFEPF

NASFPRPTLSKYIGNGVEFLNRHLSAKLFHDKESLHPLLAFLKVHCHKGKNMMLNDRIRNLDSLQYVLRK

AEEFLSTLKPDTPYSQFEHKFQEIGLERGWGDTAERVLEMIRLLLDLLEAPDPCTLETFLGRIPMVFNVV

IMSPHGYFAQDNVLGYPDTGGQVVYILDQVRALENEMLQRIKKQGLDIIPRILIITRLLPDAVGTTCGQR

LERVYGSEHCDILRVPFRDGKGMVRKWISRFEVWPYLETFTEDVAAEIAKELQGKPDLIIGNYSDGNIVA

SLLAHKLGVTECTIAHALEKTKYPDSDIYWKKFDEKYHFSCQFTADLFAMNHTDFIITSTFQEIAGSKDT

VGQYESHTAFTLPGLYRVVHGIDVFDPKFNIVSPGADESIYFPYTDEKRRLTSFHPEIDELLYSPVENEE

HLCVLKDRNKPILFTMARLDRVKNLSGLVEWYGKNTKLRELVNLVVVGGDRRKESKDLEEQAEMKKMYSH

IEKYNLNGQFRWISSQMNRVRNGELYRYICDTKGAFVQPALYEAFGLTVVEAMTCGLPTFATCNGGPAEI

IVNGKSGFHIDPYHGEKAAELLVDFFEKCKVDPAHWDKISHGGLQRIQEKYTWQIYSQRLLTLTGVYGFW

KHVSNLDRLESRRYMEMFYALKYRKLAESVPLTKE

>PtrSUS2

MSVLTRVQSIRERLDETLKTHRNEIVALLTRIEGKGKGILQHHQIIAEFEAIPEEIRKILAGGAFSEVLR

STQEAIVLPPWVALAVRPRPGVWEYVRVNVQALVVEELRVAEYLHFKEELVDGGSNGNFVLELDFEPFSA

SFPRPTLSKYIGNGVEFLNRHLSAKLFHDKESLHPLLAFLKVHCHKGKNMMLNDRIHNLDSLQYVLRKAE

EYLSSLKPETPYSQFEHKFQEIGLERGWGNTAERVLQMIQLLLDLLEAPDPCTLETFLGRIPMVFNVVIM

SPHGYFAQDNVLGYPDTGGQVVYILDQVRALESEMLLRIKQQGLDITPRILIITRLLPDAVGTTCGQRLE

KVYGSEHCDILRVPFRDEKGMVRKWISRFEVWPYLETYTEDVAAEIAKELQGKPDLIIGNYSDGNVVASL

LAHKLGVTECTIAHALEKTKYPDSDIYWKKFDEKYHFSCQFTADLFAMNHTDFIITSTFQEIAGSKDTVG

QYESHTAFTLPGLYRVVHGIDVFDPKFNIVSPGADESIYFPYTEKKLRLTSFHEEIEELLYSSVENDEHL

CVLKDRNKPILFTMARLDRVKNLTGLVEWYGKNTKLRELANLVVVGGDRRKESKDIEEQAEMKKMYSHIE

KYKLNGQFRWISSQMNRVRNGELYRYICDTKGAFVQPALYEAFGLTVVEAMTCGLPTFATCNGGPAEIIV

HGKSGFHIDPYHGVQAAELLVDFFEKCKADPSYWDKISQGGLQRIQEKYTWKIYSQRLLTLTGVYGFWKH

VSNLDHRESRRYLEMFYALKYRKLADSVPLTIE

>PtrSUS3

MANPKLERIPSMRERVQDTLSANRNVLVSLLSRYVEQGKGILHPNNLIDELDNIVCDDAARLSLKDGPFS

EVLKAAQEAIVLPPFVAVSIRPRPGVWEYVRVDVSQLNVEELTVSQYLRFKEELVDGPSNDPYVLELDFE

PFNAAFPRPTRSSSIGNGVQYLNRHLSSNMFRNKDTLEPLLDFLRVHKYKGHALMLNDRIKSVSRLQSAL

LKAEEYISKLPSETLYTEFEYTFQGMGFERGWGDTAARVLEMMHLLLDILQAPDPSTLETFLGRVPMVFN

VVILSPHGYFGQANVLGLPDTGGQIVYILDQVRALENEMLLRIQQQGLDFKPKILIVTRLIPDSKGTSCN

QRLERVSGTEHTHILRVPFRSEHGILRKWISRFDVWPYLETFAEDAASEIVAELQGIPDFIIGNYSDGNL

VASLLAYKMGVTQCTIAHALEKTKYPDSDIYWKKFDDKYHFSCQFTADVLAMNNADFIITSTYQEIAGTK

TTVGQYESHTAFTLPGLYRVVHGINVFDTKFNIVSPGADMDIYFPYSDKQKRLTTLHGSIEKMLYDSEQT

DDWIGTLTDKSKPIIFSMARLDRVKNISGLVECYGKNARLRELVNLVVVAGYIDVKKSNDREEILEIEKM

HELMKKYKLDGQFRWLTAQTNRARNGELYRYIADTKGAFVQPAFYEAFGLTVVEAMTCGLPTFATCHGGP

AEIIEHGVSGFHMDPYYPDQAAEFMADFFEKCKDDPSYWKKISDAGLQRIYERYTWKIYSERLMTLAGVY

GFWKYVSKLERRETRRYLEMFYILKFRDLVKTVPLSIEDWH

>PtrSUS4

MATLKRSDSIADNMPEALKQSRYHMKKCFAKYIEKGRRTMKLQQLLDEMENVIDDQVERTRVLEGLLGDI

WFSIQEAVVNPPYVAFSIRPSPGFWEYVKVNSANLSVEGITVTDYLKFKEMIYDENWAKDANALEVDFGA

FDFSVPHLTLSSSIGNGLGFVSKFVTSKLSGRLENAQPLVDYLLSLNRQGEKLMINETLGTVGKLQMALI

VAEVYLSGLAKDTPYQNFEISFKEWGFEKGWGDTAERVKETMRCLSEVLQAPDPMNMEKFLSRLPTVFNV

VIFSPHGYFGQADVLGLPDTGGQVVYILDQVKALEEELLLRIKQQGLNVKPQIVVATRLIPDARGTTCNL

EFEAIDGTKYSNILRVPFRVENRVLRQWVSRFDEVTTKILDLMEGKPDLIIGNYTDGNFAATLMAGKLGI

TQATIAHALEKTKYENSDVKWKELESKYHFPCQFMADIVAMNATDFIIASTYQEIAGSKDRTGQYESHAA

FTLPGLCRVVSGVNVFDPKFNIAAPGADQSVYFPHTEKQSRFTQFNPDIEELLYSKVVNDEHIGYLEDKK

KPIIFSMARLDTVKNLTGLTEWYGKNKRLRGLVNLVIVGGFFDPNKSKDREEMAEIKKMHELIEKYQLKG

QIRWIAAQTDRKRNGELYRCIADTKGAFVQPALYEAFGLTVIEAMNCGLPTFATNQGGPSEIIVDGISGF

HIDPKNGDESSNIIADFFEKCKVDPGHWNKYSLEGLKRINECYTWKIYANKLLNMGNVYSFWRQLNKEQK

LAKQRYIQLFFNLKFRELVQSVPIPTEEAQTPASEPTARTQSSAR

>PtrSUS5

MATLKRSDSIADNMPEALKQSRYHMKRCFAKYIEKGRRTMKLQQLLDEMENVIDDQVERTRVLQGLLGDI

WFSIQEAVVNPPYVALSIRPSPGFWEFVKVNSADLSVEGITATDYLKFKEMIYDENWAKDANALEVDFGA

FDFSVPHLTLSSSIGNGLGFVSKFATSKLSGRLESAQPLVDYLLSLNHEGEKLMINETLSSVRKLRMALI

VAEAYLSGLPKDTQYQNFETSFKAWGFEKGWGNTAERVKETMRCLSEVLQAPDPLNMENFFSRLPTVFNV

VIFSPHGYFGQADVLGLPDTGGQVVYILDQVKALEDELLLRIEQQGLNIKPQIVVVTRLIPEARGTKCNQ

ELESINGTKHSNILRVPFSIENKVLRQWVSRFDDVITKLLDLMQRKPDLIIGNYTDGNLAATLMASKLGI

TQATIAHALEKTKYENSDVKWKELDPKYHFSCQFMADTIAMNATDFIIASTYQEIAGSKDRPGQYESHAS

FTLPGLCRVVSGIDVFDPKFNIAAPGADQSVYFPYTEKQSRFTKFHPAIEELLYSKVVNDEHIGYLEDKK

KPIIFSMARLDTVKNLTGLTEWYGKNKRLRGLVNLVIVGGFFDPNKSKDREEMAEITKMHGLIKKYRLNG

QFRWIAAQTDRNRNGELYRCIADTKGAFVQPALYEAFGLTVIEAMNCGLPTFATNQGGPAEIIVDGISGF

HIDPQNGDESSNIIADFFEKCKVDPGYWNKFAAEGLKRINECYTWKIYAKKLLNMGNMYSFWRQLNKEQK

LAKQRYIQMLYNLQFRRLVVWSLSCNQEAWIRTAIILCHLTLLNMFIHNVFYLLLSEFNFSVYDH

>PtrSUS6

MASQTALQRSETITESMPEALRQSRYHMKKCFSRFVAPGKRLMKRQHLMDEVDESIQDKNERQKVLEGLL

GYILSCTQEAAVIPPFVAFAVRPNPGFWEYVKVNAEDLSVEGISVSEYLQLKEMVFDEKWANNENALELD

FGAMDFSTPRLTLSSSIGNGVNYMSKFMSSKLSGSSEAAKPLLDYLLALNHQGENLMINQTLDTVAKLQE

ALIVAEVVVSAFPKDTPYQDFQQRLRELGFETGWGDTAERVKETMRLLSESLQAPYPMKLQLLFSRIPNM

FNIVIFSPHGYFGQSDVLGLPDTGGQVVYILDQVRALEEELLLKIKHQGLGVKPRILVVTRLIPNAGGTK

CNQEVEPIFGTQHSHIVRVPFKTEKGVLPQWVSRFDDAADKVLEHMDSKPDLIIGNYSDGNLVASLMARK

LSITLGTIAHALEKTKYEDSDVKWKELDAKYHFSCQFTADMIAMNSADFIITSTYQEIAGSNVRPGQYES

HTAFTMPGLCRVVSGINVFDPKFNIASPGADQSVYFPYTEKQKRLTSFHPAIEELLYSNEDNHEHIGYLA

DRKKPIIFSMARLDTVKNITGLTEWFGKNTKLRNLVNLVVVAGFFDPSKSNDREEIAEIKKMHALIEKYQ

LKGQFRWIAAQTDRYRNGELYRCIADTKGAFVQPALYEAFGLTVIEAMNCGLPTFATNQGGPAEILVDGI

SGFHIDPNNGDESSNKIADFFEKCKTDAEYWNKMSAAGLQRIYECYTWKIYANKVLNMGSVYGFWRQTNK

EQKLAKQRYIEAFYNLQFNNLVGYCGQLVL

>PtrSUS7

MASAPVLKRSETIAESMPDALRQSRYHMRICFSRFVAPGRRLMKRQHIMDEVDKSIQDKNERQKVLEGLL

GYILSSTQEAAVVPPFVAFAVRPNPGFWEYVKVNAEDLSVDGISVSEYLQFKEMIFDEKWASNENALEVD

FGAMDFSTPRLTLSSSIGNGLNYMSKFMSSKLRGNSDAAKPLLDYLLALDHQGENLMINQALDSVSKLQA

ALIVAEVVVSAFPKDAPYQDFQQSLKRLGFEKGWGDTAERVKETMRMLSESLQAPEPVKLELLFSRIPNV

FNIVIFSPHGYFGQSDVLGLPDTGGQIVYILDQVRALEEELLLKIRQQGLSVKPQILVITRLIPHAGGTK

CNQEVEPIFGTKHSHIVRVPFKTEKGVLPQWVSRFDVYPYLERFAQDAADKVREHMDCKPDLLIGNYSDG

NLVASLMAQKLGTTLGTIAHALEKTKYEDSDAKWKELDPKYHFSCQFTADMIAMNTADFIITSTYQEIAG

SKNRPGQYESHVAFTMPGLCRVVSGINVFDPKFNIASPGADQTVYFPYTEKQKRLTSFHPAIEELLYNNE

DNNEHIGYLADKKKPIIFSMARLDTVKNITGLTEWYGKNAKLRNLVNLVVVAGFFDPSKSNDREEIAEIK

KMHSLIEKYQLKGQFRWIAAQSDRYRNGELYRCIADTKGAFIQPALYEAFGLTVIEAMNCGLPTFATNQG

GPAEIIVDGISGFHIDPNNGDESSNKIADFVEKCKTDAEYWNKMSATGLQRIYECYTWKIYANKVLNMGS

VYGFWRQMNKEQKLLKQRYIEAFYNLQFRNLVGYFRQLVT

>HbSUS1

MASGPSLKRSDSIADNMPEALKQSRYHMKKCFAKYVQKGRRIMKLQHLLDEMEDVIDDQMERTKVLEGLL

GDIWHSTQEALVNPPHVAFSIRPSPGFWEYVKVNSADLTVEGITATEYLKFKEVIFDESWAKDVNALEVD

FGAFDFSMPRLTLSSSIGNGLNFVSKFVTSKLSGSLENAQPLVDYLLSLNHHGEKLMINDNLNTVSKLQM

ALIVAEVYLSGLSKDTSYQNFELSFKEWGFEKGWGDTTERVKETMRSLSEVLQAPDPVNMEKFFSRVPTI

FNVVIFSPHGYFGQSNVLGLPDTGGQVVYILDQVKALEEELLLRIKHQGLDVKPHIIVVTRLIPEARGTK

CNQELEAINGTKHSNILRVPFSIEDRVLRQWVSRFDVYPYIEKFTQDVTVKVLDLMDGKPDLIIGNYTDG

NLAATLMANNLGITQATIAHALEKTKYEDSDIKWKELDPKYHFSCQFIADTIAMNAADFIIASTYQEIAG

SKERPGQYESHTAFTLPGLCRIVSGINVFDPKFNIAAPGADQSVYFPNTEKQKRFTQFHPAIEELLYSKE

ENEEHIGYLADRSKPIIFSMARLDIVKNLTGLTEWYGKNKRLRNLVNLVIVGAFFDPTKSKDREEMAEIR

KMHALIEKYQLKSQFRWIAAQTDRQRNGELYRGIADTKGAFVQPALYEAFGLTVIEAMNCGLPTFATNQG

GPAEIIVDGVSGFLIDPNNGDESSNKIADFFAKCKEDPGHWNKFSVDGLKRINECYTWKIYANRVLNMGC

MYTFWRQLTKEQKQAKQRYIQLLYNLQFRSVVKNVPIPTEEAQQQAEPKPESKAASRQVT

>HbSUS2

MGTPKLARIPSMRDRVEDTLSAHRNELVSLLCRYVDQGKGILQPHTLIDELDNIVSEDEARLGLRDGPFG

EILKSAQEAIVLPPFVAIAIRPRPGVWEYVRVNVYELSVEQLSVSEYLRFKEELVDGPSNDPYVLELDFE

PFNADVPRPNRSSSIGNGVQFLNRHLSSIMFRNKDCLEPLNDFLRAHKYKGHALMLNDRIQSISGLQSAL

AKAEEYISKLPPDSPYSEFEYKLQELGFERGWGDTAARVLETMHLLLDILQAPDPLSLETFLGRIPMVFN

VVILSPHGYFGQANVLGLPDTGGQVVYILDQVRALENEMLLRIQKQGLDFKPRILIVTRLIPDAKGTTCN

QRLERVSGTEHTHILRVPFRSEKGILRKWISRFDVWPYLETFAEDVASEIVAELQGIPDFIIGNYSDGNL

VASLLAYKMGITQCTIAHALEKTKYPDSDIYWKNFDDKYHFSCQFTADLLAMNNADFIITSTYQEIAGTK

NTVGQYESHTAFTLPGLYRVVHGIDVFDPKFNIVSPGADMSIYFPYSEKQKRLTALHASIEKMLYDPEPT

DEWIGKLSDKSKPLIFSMARLDRVKNITGLVEIYGKNTKLRELVNLVVIAGYIDVKKSRDREEIAEIEKM

HDLMKKYNLDGQFRWITAQTNRARNGELYRYIADTKGAFVQPAFYEAFGLTVVEAMTCGLPTFATCHGGP

AEIIEHGMSGFHIDPYHPDQAAEILVDFFQKCKEDPSHWNKISDAGLQRIYERYTWKIYSERLLTLAGVY

GFWKYVSKLDRRETRRYLEMFYILKFRDLVKTVPLAIDDQH

>HbSUS3

MAERVITRVHSFRERLDETLSAHRNEIVALLSRIEGKGKGILQHHHIIAEFEAIPEENRKKLLDSVFGEV

LRSAQEAIVLPPCVAFAVRPRPGVWEYIRVNVHALVVEELRVAEYLHFKEELVNGSVNGNVNGKFVLELD

FEPFNASFPRPTLSKYIGNGVEFLNRHLSAKLFHDKESLHPLLEFLKVHCHKGKSMMLNDRIHSLDSLQY

VLRKAEEYLTALSPETPYSQFEHKFQEIGLERGWGDTAERVLEMIRLLLDLLEAPDPCTLETFLGRIPMV

FNVVIMSPHGYFAQDNVLGYPDTGGQVVYILDQVRALETEMLHRIKQQGLDITPRILIVTRLLPDAVGTT

CGQRLEKVFGTEHSDILRVPFRTEKGIVRKWISRFEVWPYLETYTEDVATEIGKELQGKPDLIIGNYSDG

NIVASLLAHKLGVTECTIAHALEKTKYPESDIYWKKFDEKYHFACQFTADLIAMNHTDFIITSTFQEIAG

SKDTVGQYESHTAFTLPGLYRVVHGIDVFDPKFNIVSPGADMSIYFPYTEEKLRLTSFHPEIEELLYSPV

ENEEHLCVLKDRNKPIIFTMARLDRVKNLTGLVEWYGKNAKLRELANLVVVGGDRRKESKDLEEQAEMKK

MHGLIEKYNLNGQFRWISSQMNRVRNGELYRYICDTKGVFVQPALYEAFGLTVVEAMTCGLPTFATCNGG

PAEIIVHGKSGFNIDPYHGDQAAELLVDFFEKCKVDPSYWNNISHGAMQRIQEKYTWQIYSQRLLTLTGV

YGFWKHVSKLDRLESKRYLEMFYALKYHKLAESVPLTVED

>HbSUS4

MAERVITRVHSIRERLDETLSAHRNEIVALLSRIEGKGKGILQHHHIIAEFEAIPEENRKKLLDSVFGEV

LRSTQEAIVLPPWVALAVRPRPGVWEYIRVNVHALVVEELRVAEYLHFKEELVDGSVNGNFVLELDFEPF

NASFPRPTLSKYIGNGVEFLNRHLSAKLFHDKESLHPLLEFLKVHCHKGKNMMLNDRIHNLDSLQYVLRK

AEEYLTALSPETPYSQFEHKFQEIGLERGWGDTAERVLEMIRLLLDLLEAPDPCTLETFLGRIPMVFNVV

IMTPHGYFAQDNVLGYPDTGGQVVYILDQVRALETEMLHRIKQQGLDITPRILIITRLLPDAVGTTCGQR

LEKVFGTEHSDILRVPFRTEKGIVRKWISRFEVWPYLETYTEDVATEIGKELQGKPDLIIGNYSDGNIVA

SLLAHKLGVTECTIAHALEKTKYPESDIYWKKFDEKYHFSCQFTADLIAMNHTDFIITSTFQEIAGSKDT

VGQYESHTAFTLPGLYRVVHGIDVFDPKFNIVSPGADESIYFAYTEEKRRLTSFHPEIEELLFSPVENEE

HLCVLKDRNKPIIFTMARLDRVKNLTGLVEWYGKNAKLRELANLVVVGGDRRKESKDLEEQAEMKKMHGL

IEKYNLNGHFRWISSQMNRVRNGELYRYICDTKGVFVQPALYEAFGLTVVEAMTCGLPTFATCNGGPAEI

IVHGKSGFNIDPYHGDQAAELLVDFFEKSKADPSHWNNISQGAMQRIQEKYTWQIYSQRLLTLTGVYGFW

KHVSKLDRRESRRYLEMFYALKYRKLAESVPLTVED

>HbSUS5

MANPKLGRIPSMRERVEDTLSAHRNELVSLLCRYVDQGKGILQPHTLIDEFDNIVGEDEARLGLRDGPFG

EILKSAQEAIVLPPFVAIAIRPRPGVWEYVRVNVFELSVEQLSVSEYLQFKEELVDGPSHDPYVLELDFE

PFNADVPRPNRSSSIGNGVQFLNRHLSSKMFRDKDCLEPLNDFLRAHKYKGHALMLNDRIQSISRLQSAL

AKAEEYISKLPPDTPYTDFEYTLQGLGFERGWGDTAARVQETMHLLSDILQAPDPSSLETFLGRIPMVFN

VVILSPHGYFGQANVLGLPDTGGQVVYILDQVRALENEMLLRIQKQGLDFKPRILIVTRLIPDAKGTTCN

QRLERVTGAEHTHILRVPFRSEKGILRKWVSRFDVWPYLETFAEDVASEIVAELQGFPDFIIGNYSDGNL

VASLLAYKMGVTQCTIAHALEKTKYPDSDIYWKNFDDKYHFSCQFTADLLAMNNADFIITSTYQEIAGTK

NTVGQYESHTAFTLPGLYRVVHGIDVFDPKFNIVSPGADMSIYFPYSVKEKRLTALHGSIEKMLYDPEPT

AEWIGTLSDKSKPLIFSMARLDQVKNITGLVEMYGKNTRLRELVNLVVVAGYIDVKKSKDREEIAEIEKM

HDLMKKYNLDGQFRWITAQTNRARNGELYRYIADTKGAFVQPAFYEAFGLTVVEAMTCGLPTFATCHGGP

AEIIENGKSGFHIDPYHPDQAAQIMVDFFQQCKEDPSHWNKISNAGLQRIYERYTWKIYSERLLTLAGVY

GFWKYVSKLERLETRRYLEMFYILKFRDLVKTVPLPVDDQH

>HbSUS6

MASGPVLKRSETIAESMPDALRQSRYHMKICFSSFVATGKKLLKRQHIMDEMEKSIQDKVERKRVLEGLL

GYIMSATQEAAVIPPYVAFAVRPNPGFWEYVKVNAEDLSVDGISASEYLQFKEMIFDENWASDENALEID

FGAIDFSTPRLTLSSSIGNGMKYISKFMSSKLNGSSGSAKPLLDYLLALDYQGENMMINEKLDTVAKLQV

ALLGAEDVLSAFPKHTPYQDFQHSLKELGFEKGWGNTAERVKETMRMLSESLQAQEPAKLELFFGRLPNI

FNIVIFSPHGYFGQADVLGLPDTGGQVVYILDQVRALEEELLLRIKQQGLNMKPQILVITRLIPDARGTK

CNQEMEPIIDTKHSNILRVPFMTEKGVLPQWVSRFDVYPYLEKFAQDAADKVLEHMECKPDLIIGNYSDG

NLVASLMANRLGITLGTIAHALEKTKYEDSDAKWKQLDPKYHFSCQFTADMIAMNTADFIITSTYQEIAG

SKDRPGQYESHETFTMPGLCRVVSGINVFDPKFNIAAPGADQSVYFPCTEKRRRLTSFYPAIEELLYNKE

DNNEHIGYLADRKKPIIFSMARLDTVKNITGLTEWYGQNKRLRNLVNLVVVAGFFDPSKSKDREEIAEIN

KMHALIAKYQLVGQIRWIAAQTDRYRNGELYRCIADTKGAFVQPALYEAFGLTVIEAMNCGLPTFATNQG

GPAEIIVDGVSGFHIDPNNGDESSKKIADFFEKCKTDPEYWNKMSTAGLQRIYECYTWKIYANKVLNMGS

IYGFWRKLNKEQKFAKQRYIETFYNLQFRNLVKNVPVPSVEPRKLPSLPSSAATSKPQEQAPSAPSKPKK

SQPTAPMEISEPQTTPRQEETEKKQLVSTQSNRVWISWSWWFLIITSLFAVWYVLMKLYSRFTR

>PpSus1

MAERALTRVQSLRERLDVTLSAHRNEIVALLSRIVNKGKGFMQPHELVAEFEAIPESNRQKLLDGAFGEV

LTHTQEVIVLPPWVALAVRPRPGVWEYIRVNVDALVVEELQVPEYLHFKEELVDGSANGNFVLELDFEPF

NASFPRPTLSKSIGNGVEFLNRHLSAKLFHDKESMHPLLEFLRVHCYKGKNMMLNDRIHNVNELQHVLRK

AEDYLSTIAPETPYKQFEDKLQKLGLERGWGDTAERVLEMIQLLLDLLEAPDPCTLEKFLGQIPMVFNVV

ILSPHGYFAQDNVLGYPDTGGQVVYILDQVRALETEMLKRIKQQGLDITPRIIILTRLLPDAVGTTCGER

LEKVYNTEYSHILRVPFRTEKGIVRRWISRFEVWPYLETYAEDAIQELSKELHGKPDLIIGNYSDGNIVA

SLMAHKLGVTQCTIAHALEKTKYPDSDLYWKKLDDKYHFSSQFTADLIAMNHTDFIITSTFQEIAGSKDT

VGQYESHTAFTLPGLYRVVHGIDVFDPKFNIVSPGADMSIYFPYSEKEKRLTSFHPEIEELLYSQVENKE

HLCVLKDRNKPIIFTMARLDRVKNITGLVEWYGKNAKLRELVNLVVVAGDRRKESKDNEEKAEMKKMYEL

IDTYNLNGQFRWISSQMNRVRNGELYRVIADTKGAFVQPAVYEAFGLTVVEAMTCGLPTFATCKGGPAEI

IVHGKSGYHIDPYHGDQAADILVDFFEKSRADPSHWDKISQGGLQRIYEKYTWQIYSERLLTLTGVYGFW

KHVSNLDRLESRRYLEMFYALKYRKLAESVPRAEEE

>PpSus2

MRKSIEDTLAAHRNELVSLLSRYVDRGNGILQPHQMINELENVIAEDEGMQKLKDSPFSIVLQSAQEAIV

QTPFVALALRPRPGVWEYVRVNVYELSVDLLSVAEYLWFKEELLDGECNDKYVLELDLEPFNATFPRPTR

SSSIGNGVQFLNRHLSSIMFRNKESLEPLLDFLRTHKHDGHAMMLNDRIQSIPRLQSALAKAEEYLSKLL

ATTPYSDFEFDLQGMGFERGWGDTAQRVSEMVHLLLEILQAPDPSTLETFLGRIPMVFNVVIVSPHGYFG

QANVLGLPDTGGQVVYILDQVRALENEMLLRIQNQGLDVIPKILIVTRLIPDAKGTTCNQRLERISGTEH

THILRVPFRTENGIMRKWISRFDVWPYLETFAEDASNEIAAELQGVPDLIIGNYSDGNLVATLLSNKLGI

SQCNIAHALEKTKYPDSDIYWKKHEDKYHFSSQFTADLIAMNNADFIITSTYQEIAGSKNNVGQYESHTA

FTLPGLYRVVHGIDVFDPKFNIVSPGADMCIYFPYSEKERRLTALHGSIEELLYGAEQNEEHIGILSDRS

KPIVFSMARLDRVKNLTGLVECYGKSTKLRELVNLVVVGGYIDAKNSRDREEVAEIEKMHDLVKKYNLSG

QFRWIAAQMNRARNGELYRYIADTKGVFVQPAFYEAFGLTVVEAMTCGLPTFATCHGGPAEIIEHGISGF

HIDPYHQDQVAALLIDFFDQCQKHPGYWEKISEAGLKRIYERYTWKIYSERLLNLAGVYGFWKHVSKLER

RETRRYLDMFYILKYRNLVKSIPLAVDEQH

>PpSus4

MGFSTYILVSLIFRCFMQEVCAGEEAIVLPPFVALAFRPRPGVWEYVRVNVYELSVDHLTVAEYLQFKEE

LIDGECNDNYVLELDFEPFNAAFPRPTRSSSIGNGVQYLNRHLSSIMFSKKESLEPLLDFLRTHKHDGHA

MMLNDRIHSILRLQSALAKAEEYLSKFPPTTPYSEFQFDLQGMGFERGWGDTAQRVSEMVHLLLEILQAP

DPSTLESFLGRIPMVFNVVIVSPHGYFGQANVLGLPDTGGQVVYILDQVRALENEMLLRIQNQGLGVIPK

ILIVTRLIPDAKGTTCNQRLERVSSTEHTHILRVPFRTKNGILRKWLSRFDVWPYLETFAEDASNEIAAV

FQGVPDLIIGNYSDGNLVATLLSYKLGITQCNIAHALEKTKHPDFDIYWKKHEDKYHFSSQFTADLIAMN

NADFIITSTYQEIAGSKNNVGQYESHTAFTLPGLYRVVHGIDVFDPKFNIVSPGADMCIYFPYSEKERRL

TALHGSIEELLYGAEQNEEHIGILSDRSKRIVFSMARLDRVKNLTGLVEFYAKSTKLRELVNLVVVGGYI

DVKNCRDREEMAEIEKMHGLIKKYNLSGQFRWIVTQMNHARNGELYRYVADTKGVFVQPAFYEAFGLTVV

EAMSCGLPTFATCHGGPAEIIEHGISGFHIDPYHPDQVAALLVDFFDQCQKDPGYWERISETGLKRIFER

YTWKIYSERLLNLAGVYGFWKHVSKLERRETRRYLEMFYILTYRNLVSSVILPSMTQ

>PpSus5

MASGAAIKRSESIAESMPEALRQSRYHMKRCFAKYIEKGKRIMKLPHLMSEMETVIDDKVERNQVLEGVL

GYILCSTQEAVVIPPFVVFAIRPNPGYWEFVKVSSEDLSVESITVRDYLKFKETLYDEKWSNDENTLEVD

FRAIDFSTPHLTLSSSIGNGLNFVSKFTSSKLAGRLENAQPLVDYLLSLNHEGENLILNENLNTASKLQT

ALIVTEVYLSALPKDMPYQNFELRFKEWGFEKGWGDTAERTKETMKLLSEVLQAPDPLNLERFFSRLPII

FNVVIFSPHGYFGQADVLGLPDTGGQVVYILDQVQALEEELLLRIKQQGLTVKPQILVVTRLIPEAKGTK

CNQELEPINGTKYSNILRVPFRTEKGILRRWVSRFDIYPYLELFAQDASAKVLDIMEGKPDLIIGNYSDG

NLVASLMASKLGITQATIAHALEKTKYEDSDIKWKELDPKYHFSCQFLADTISMNATDFVIASTYQEIAG

SKDRPGQYESHTAFTLPGLCRVVSGINVFDPKFNIAAPGADQSVYFPYTEKQKRLTSFHPAIEELLYSKE

DNSEHIGFLADRKKPIIFSMARLDTVKNITGLVEWYGKNKRLRNLVNLAVVGGFFDPSKSKDREEIAEIK

KMHTLIEKYQLRGQIRWIAAQTDRNRNGELYRCIADTRGAFVQPALYEAFGLTVIEAMNCGLPTFATNQG

GPAEIIVDGISGFHIDPNNGDEASNKIADFFEKSKTDATYWDRFSKAGLQRIYECYTWKIYANKVLNMGS

TYTFWRQLNKEQKQAKQRYIQMFFNLQYRNLVKNVPIPSDEAEQPVPKPTAKSQPTPRHVNLN

>PpSus6

MASTPALKRSDTIAETMPDALRQSRYHMKKCFARFVAMGKRLMKMQHVMEELEKSIEDKHERSKVLEGLL

GYIISSTQEAAVVPPYVAFAVRPNPGFWEFVKVNADDLAVDGITASEYLKFKEMIFDDNWANDENALEID

FGGIDFATPRMKLPSSIGNGLNFILKLISSRLATANSSDYAKPLLDYLSQLHYHGENLMINETLNTVAKL

QTALIQAEVVVSTLPKDTPFPNFEHRLKELGFEKGWGDTAERVGETMKMLSEVLQAPDSAKLECFFSRLP

NTFNIVIFSPHGYFGQSDVLGLPDTGGQVVYILDQVRALEEELLLRIKQQGLAVKPQILVVTRLIPDARG

TKCNQELEAIINTKHSHILRVPFRTHRGILRQWLSRFDIYPYLETFAQDATAKILERMECKPDLIIGNYS

DGNLVASLTAGKLGITQGTIAHALEKTKYEDSDAKWKEFDPKYHFSCQFTADIISMNSADFVITSTFQEI

AGGKDRPGQYESHTAFTMPGLYRVVSGIDVFDPKFNIAAPGADQSVYFPCSEKQRRFTKFHPAIEELLYN

KAENDEHIGYLADKKKPIIFSMARLDTVKNLSGLVEWFGKNKRLRSLVNLVIVGGFFDPSKSKDREEIAE

IKKVHALVQEYRLTGQFRWIAAQTDRYRNGELYRCIADTRGAFVQPALYEAFGLTVIEAMNCGLPTFATN

QGGPAEIIVDGVSGFHIDPNNGDESSNKIADFFEKCKTDGEYWNKMSAAGLQRIYECYTWKIYANKVLNM

GSTYGFWRQLRKEQKLANQTYIHMFYSLLFRNLARNVGVPSDGFEQPTAKAITAAGQPTPVAPPTSPIPQ

LITPTPRERDEGLEEKQKQQQLGEPRSPTARCILNCCCVIIGFLILVYYKIRNMYNYN

>CitSUS1

MAERALTRVHSLRERLDETLSAHRNEILALLSRIEGKGKGILQNHQLIAEFESISEENRKHLTEGAFGEVLRATQEAIVLPPWVALAVRPRPGVWEYIRVNVHALVVEELLVAEYLHFKEELVDGGSNGNFVLELDFEPFNASFPRPTLSKSIGNGVEFLNRHLSAKLFHDKESMHPLLEFLRVHCHKGKNMMLNDRIQNLNSLQHVLRKAEEYLTTVVPETPFSELALRFQEIGLERGWGDTAERALEMIQLLLDLLEAPDPCTLETFLGRIPMVFNVVILTPHGYFAQDDVLGYPDTGGQVVYILDQVRALEDEMLLRIKQQGLDITPQILIITRLLPDAVGTTCGQRLEKVYGTKYSDILRVPFRTEKGVVRKWISRFEVWPYLETYTEDVAVEIAKELQGKPDLIIGNYSDGNIVASLLAHKLGVTQCTIAHALEKTKYPDSDIYWKNLDDKYHFSCQFTADLIAMNHTDFIITSTFQEIAGSKDTVGQYESHTAFTLPGLYRVVHGIDVFDPKFNIVSPGADMSIYFPYTEEKRRLKSFHPEIEELLYSDVENKEHLCVLKDRNKPILFTMARLDRVKNLTGLVEWYGKNAKLRELVNLVVVGGDRRKESKDLEEQAEMKKMYSLIDQYKLNGQFRWISSQMNRVRNGELYRYICDTKGAFVQPALYEAFGLTVVEAMTCGLPTFATCKGGPAEIIVNGKSGYHIDPYHGEQAAEILVDFFEKCKADPSYWDKISLGGLKRIEEKYTWKIYSQRLLTLTGVYGFWKHVSNLDRLESRRYLEMFYALKYRKLAESVPLAVE

>CitSUS2

MFRFPPYFVFVCYSIERLGCGTYKRQQILSLLEAESNGAAIADVLNATQEAAVSSPWVAFAVRTSPGVWCYIRVNVQTVDVEEISVSKYLLFKEEIVDGRKSNGNFAFEVDFEPFRALPHPTLSNSIGHGMEFLNRHMSAKLFNDKESMQSLLEFLRVHSHMGKNMMLNEKIQDLGTLQSSLRMAEKYLSMLAPDTSYAEFEQKFQEIGLERGWGDNAEHVLGMIQLLLDLLQAPESSTLETFLGKIPRVFNVVIFTPHGYFAQDNVLGYPDTGGQVVYILDQVRALENEMLLRIKQQGLDITPRILIITRLLPDAVGTTCGQRVEKVYGTKYSDILRVPFRTEEGIVRKWISRFEVWPYLETFTEDVATEIIQELQCKPDLIIGNYSDGNIVASLLAHKLDVTQCTIAHALELTKYPDSDINWKKLDDKYHFSCQFTADLFAMNRTDFIITSTFQEIAGSKDTVGQYESHTAFSLPGLYRVVNGIDAFDPKFNIVSPGADMTIYFPYMEEKRRLKHFHSEIEELLYSPVENKEHLCVLKDSSKPILFTMARLDRVKNLTGLVEWYGKNAKLRELVNLVVVGGDRRKESKDLEEQAEMKKMYGLVDTYKLNGQFRWISSQMNRVRNGELYRYICDTKGAFVQPALYEAFGLTVVEAMTCGLPTFATCNGGPAEIIVHGKSGFHIDPYKGDQAAGILVDFFEKCKVDPGHWDEISQGGLKRIQEKYTWKIYSERLLNLSGVYGFWKHLSKLDSREKNRYLEMFYSLMYRKQVQTVPLAVDE

>CitSUS3

MAAPKLSRIPSIRERVEDTLSVHRNELVSLLSRYVAQGKGILQPHVLIDELDNIFGDDEGRQNLRDGPFSEVIKSAQEAIVLPPFVAIAVRPRPGVWEYVRVNVYELSVEQLSVSEYLHFKEELVDAAFNERFVLELDFEPFNATFPRPNRSSSIGNGVQFLNRHLSSSMFRNKDCLEPLLDFLRAHKYKGHLLMLNDRIQSISRLQSSLSKAEDHLSKLPPDTPFSQFEYVLQGMGFEKGWGDTAEHVLEMMHLLLDILQAPDPSTLEKFLGRLPMVFNVVILSPHGYFGQANVLGLPDTGGQVVYILDQVRALENEMLLRIKRQGLDISPKILIVTRLIPDAKGTTCNQRLERVSGTEHTHILRVPFRSEKGILRQWISRFDVWPYLETFTEDVGSEITAELQGFPDFIIGNYSDGNLVASLLAYKMGITQCTIAHALEKTKYPDSDIYWKKFDEKYHFSCQFTADLIAMNNADFIITSTYQEIAGTKNTVGQYESHTAFTLPGLYRVVHGIDVFDPKFNIVSPGADMDIYFPYSEKQKRLTALHGSIEQLLFDPEQNDEHVGTLSDRSKPIVFSMARLDHVKNMTGLVECYGKNSQLRELVNLVVVAGYIDVNKSKDREEIAEIEKMHELMKTYKLDGQFRWIAAQTNRARNGELYRYIADTKGAFVQPAFYEAFGLTVVEAMTCGLPTFATCHGGPAEIIEHGASGFHIDPYHPDQAAELMADFFGKCKENPSHWKKISDGGLKRIYERYTWKIYSERLMTLAGVYGFWKYVSKLERRETRRYLEMFYILKFRDLVKSVPLASENQH

>CitSUS4

MSSSPSLKRSDTIADTMPDALRQSRYYMKKCFSRFVAKGKRLMKRHHLMDEVEKSIEDKIERGKVLEGLLGYILSSTQEAAVVPPNVAFAVRPNPGSWEYVKVNSEDLTVDGINVLEYLKFKETIFDQDWAKDENALELDFGAMDFSSPRLTLSSSIGNGVNYVSKFMSTRLSANSEKAKQFLDYLLALNHRGEQLMINDTLDTVDKLQAALIVAEVSISDLPKDTPYQEFQQRFKEWGFEKGWGNTAERVRETMRLFSEVLQAPDAAKLQVLFSRLPNMFNVVIFSPHGYFGQADVLGLPDTGGQVVYILDQVRALEEELLLRIKQQGLSVKPQILVVTRLIPNSKGTKCNQELEPIYDTKHSHILRIPFKTEQAILPQWVSRFDIYPYLGRFAQDATAKILDLMEGKPDLIIGNYSDGNLVASLMASKLGITQATIAHALEKSKYEDSDAKWKELDPKYHFSCQFTADLIAMNQTDFIITSTYQEIAGSKDRPGQYESHTAFTMPGLCRVVSGINVFDPKFNIAAPGADQSVYFPYTEKQKRLTSFHPDIEELLYSKEDNSEHIGYLADRKKPIIFSMARLDTVKNITGLTEWYGKNKRLRNMVNLVVVAGFFDPSKSHDREEIAEIKKMHTLIEKYQLQGQFRWIAAQTDRYRNGELYRCIADTKGAFVQPALYEAFGLTVIEAMNCGLPTFATNQGGPAEIIIDGVSGFHIDPNNGDESSNKIADFFEKCKTDAGYWNQMSAAGRQRIYECYTWKIYANKVLNMGSIYGFWRQINKEPKEAKQRYIQMFYSLLFRKLASNVPIKVPEPLQSAQTSPVESQQPAAATGIAKPQPPASAVIDKPNQQEKTAQQKKRHVRKTMTVI

>CitSUS5

MASATSLKRSDSIADNMPDALKQSRYHMKRCFVRYIEKGKRIMKLHDLMDELNEVIDDEDVRTQVLEGLLGYILCSTQEAVVMPPHVAFAIRPNPGFWEFVKVNSDDLSVEAITVTDFLKFKELVFDEDWAKDENALEVDFGAYEFSLPQLTLSSSIGNGISFVSKFVTAKLSGRQDCAQPLVDYLLSLDHQGEKLMINDNLNTAEKLQMALIVAEVSLSTLPKDTPYQKFELRFKEWGFEKGWGHTAERVRETMRSLSEVLQAPDPLHMEKFLSSLPILFNVVIFSPHGYFGQADVLGLPDTGGQVVYILDQVKALEEELLLRIKQQGLYIKPQIVVVTRLIPDARGTKCNQELEPIEGTKHSNILRVPFKTDKGILHRWVSRFDVYPYLEGFAQDATTMILELLGGKPDLIIGNYSDGNLVASLMASKLGITQATIAHALEKTKYEDSDVKWKELDPKYHFSCQFIADTIAMNATDFIIASTFQEIAGSKDRPGQYESHTAFTLPGLCRVVKGIDVLDPKFNIAAPGADQSVYFPYTEKQRRLTKFHPEIEELLYNKEDNNEHIGYLADRKKPIIFSMARLDVVKNLTGLTEWYGKNKRLRNLVNLVIVGAFFDPSKSKDREETAEIKKMHALMEKYQLKGQMRWIAAQSDRLRNGELYRCIADTKGAFVQPALYEAFGLTVIEAMNCGLPTFATNQGGPAEIIVDGVSGFHIDPYNGDESSDKIADFFEACKVDPTYWNKFSTEGLKRINECYTWKIYANKMLNMGCMYSFWKQLNKGQKLAKQRYIEMFYNLLFKNLVKNVPVPNEEAQQPMSEPAVKPQHSLRQARSSTNMLHF

>CitSUS6

MPHSKLDSMRDRVQDTLSVHRNELVSLLSRYAGKGKGILQRHHLTEEMDDIVKEDEGMQKLSKSPFMKVLQSAQEAIILPPFVVLAVRPRPGVWEYVRVNVYELSVDRLNVAEYLKSKEELVEGQSGDNYVLELDLEPFNATFPRPTRSSSIGNGVQFLNRHLSSVMFRNKESLEPLLNFLRVHKHDGFVMMLNDRIQSISKLQSALQRAEEYLSKFPPDTPYSEFEFEIQGMGFERGWGDTAQRVSEMVHLLLDILQAPDPATLETFLGRIPMVFNVVIVSPHGYFGQANVLGLPDTGGQVVYILDQVRALENEMLLRIQNQGLDVIPKILIVTRLIPDAKGTTCNQRLERISGTEHTHILRVPFRTENGILRKWISRFDVWPYLETFAEDASNEIAAELQGVPDLIIGNYSDGNLVATLLSYKLGVTQCNIAHALEKTKYPDSDLYWRKFEEKYHFSSQFTADLTAMNNADFIITSTYQEIAGSKNNVGQYENHTAFTLPGLYRVVHGIDVFDPKFNIVSPGADMCIYFPYSDKEKRLIALHGQIEDLLYDPKQNDEHVGILNDRSKPLIFSMARLDGVKNLTGLVECYGKSSKLRELVNLVVVGGYMDVKNSRDREEMAEIEKMHGLIKQYNLHGQFRWISAQMNRVRNGELYRYIADTRGVFVQPAFYEAFGLTVVEAMTCGLPTFATCHGGPAEIIEHGVSGFHIDPYHPDQVAELMIEFFEKCYNDPSHWNKISDGGLKRIYERYTWKIYSERLLTLAGVYGFWKYVSKLDRRETRRYLEMFYILKFRDLAKSVRLAVDEQH

>GmSUS1

MASAPALKRTDSVVDNMPDALRQSRYHMKRCFAKYLEKGRRIMKLHHLMEEMELVIDDKSERSQVLEGILGFILSSTQEAVVDPPYVAFAIRPNPGVWEFVKVSSEDLSVEAITPTDYLKFKERVHDEKWATDENSFEADFGAFDSQIPLLTLSSSIGNGLEFTSKFLTSKLTGKLEKTQAIVDYLLTLNHQGESLMINDSLNSAAKLQMALVVADAFLSGLSKDTAYQNFELRFKEWGFERGWGDTAGRVKETMRTLSEVLQAPDPMNLEKFLSNLPIIFNVVIFSVHGYFGQADVLGLPDTGGQVVYILDQVKSLEAELLLRIRQQGLNVKPQILVVTRLIPDARGTKCHHELEPISDTKHSHILRVPFQTDKGILRQWISRFDIYPYLERFTQDATAKILEFMEGKPDLVIGNYTDGNLVASLMARKLGITQGTIAHALEKTKYEDSDVKWKELDPKYHFSCQFMADTVAMNASDFIITSTYQEIAGSKDRPGQYESHAAFTLPGLCRVVSGINVFDPKFNIVAPGADQSVYFPYTEKEKRLSQFHPAIEDLLFSKVDNIEHIGYLADRRKPIIFSMARLDVVKNLSGLVEWYGKNKRLRNLVNLVIVGGFFDPSKSKDREEMAEIKKMHDLIDKYQLKGQFRWIAAQTNRYRNGELYRCIADTRGAFVQPALYEAFGLTVIEAMNCGLPTFATNQGGPAEIIVDGVSGFHIDPLNGEESSNKIADFFEKCKVNQSQWNVISEAGLQRINECYTWKIYANKMVNMGNIYTFWRQVNKEQKEAKQRYIQMFYNLIFKNLVKTVPAPSDEPQQPVGKQPSLKSRSTGRSQSRLQRLFGN

>GmSUS2

MSTQPKLGRISSIRDRVEDTLSAHRNELISLLSRYVAQGKGILQPHNLIDELDNIPGDDEAIVDLKNGPFGEIVKSAKEAIVLPPFVAIAVRPRPGVWEYVRVNVSDLSVEQLSISEYLSFKEELVDGKINENFVLELDFEPFNATFPRPTRSASIGNGVQFLNRHLSSIMFRNKDSLQPLLDFLRAHKYKGHALMINDRVQTISNLQSALAKTEDYLSKLASDTLYSEFEYVLQGMGFERGWGDTAERVLEMMHLLLDILQAPDPSTLETFLGRVPMVFNVVILSPHGYFGQANVLGLPDTGGQVVYILDQVRALENEMLLRIKKQGLDFTPRILIVTRLIPDAKGTTCNQRLERVSGTDHTHILRVPFRSESGTLRKWISRFDVWPYLETYAEDVASEIAAELQGYPDFIIGNYSDGNLVASLLAYKMGVTQCTIAHALEKTKYPDSDLYWKKFEDKYHFSCQFTADLIAMNNADFIITSTYQEIAGTKNTVGQYESHTGFTLPGLYRVVHGIDVFDPKFNIVSPGADMSIYFPYSEKQNRLTALHGSIEKLLFDPEQTDEYIGSLKDKSKPIIFSMARLDRVKNITGLVECFGKNSKLRELVNLVVVAGYIDVKKSSDREEIAEIEKMHELMKKYNLNGDFRWIAAQTNRARNGELYRYIADTQGAFIQPAFYEAFGLTVVEAMTCGLPTFATCHGGPAEIIEHGISGFHIDPYHPDQASELLVEFFQKSKEDPDHWKKISNGGLQRIYERYTWKIYSERLMTLAGVYSFWKYVSKLERRETRRYLEMFYILKFRDLANSVPLAKDDAS

>GmSUS3

MANHPLTHSHSFRERFDETLTGHRNEILALLSRLEAKGKGILQHHQVVAEFEEIPEESRKKLQDGVFGEVLRSTQEAIVLPPFVALAVRPRPGVWEYLRVNVHMLVVDELRPAEYLRFKEELVEGSSNGNFVLELDFEPFNASFPRPTLNKSIGNGVEFLNRHLSAKLFHDKESMQPLLEFLRLHSYKGKTMMLNDKVQSLDSLQHVLRKAEEYLISVAPETPYSEFENRFREIGLERGWGDTAERVLEMIQLLLDLLEAPDPCTLETFLGRVPMVFNVVILSPHGYFAQDNVLGYPDTGGQVVYILDQVRALENEMLNRIKKQGLDITPRILIITRLLPDAVGTTCGQRLERVYDTEYCDILRVPFRTEKGIVRKWISRFEVWPYLETYTEDVALELAKELQAKPDLIVGNYSDGNIVASLLAHKLGVTQCTIAHALEKTKYPESDIYWKKFEEKYHFSCQFTADLFAMNHTDFIITSTFQEIAGSKDTVGQYESHTAFTLPGLYRVVHGIDPFDPKFNIVSPGADMGIYFPYTETERRLTEFHSDIEELLYSSVENEEHICVLKDRNKPIIFTMARLDRVKNITGLVEWYGKNARLRELVNLVVVAGDRRKESKDLEEKAEMKKMYGLIETYKLNGQFRWISSQMNRVRNGELYRVICDTRGAFVQPAVYEAFGLTVVEAMTCGLPTFATCNGGPAEIIVHGKSGYHIDPYHGDHAAEILVEFFEKSKADPSHWDKISQGGLKRIHEKYTWQIYSDRLLTLTGVYGFWKHVTNLERRESKRYLEMFYALKYRKLVYFFHYYTIQWLHL

>GmSUS4

MASTASNSALKRSDSITDSMPEALKQSRFHMKRCFARFVASGKRLMKQQHVMDDAEKTVEDKVERKKLLDGMLGYIFSCTQEAAVVPPYIAFAVRPNPGFWEYIKVNADDLQVEGIEAVEYLKYKEMIFDEKWANDENALELDFGAIDFSTPRMVLSSSIGNGLNFTTKILTSRLSESSQNINPLLDYLLSLNYQGENLMIKDTLNTMPKLQQALKVAEAYVSALHKDTPYQKFEDRFKEWGFDKGWGNTAGRVKETMKLLSEVLESADPVKLESLFSRLPNMFNIVILSIHGYFGQADVLGLPDTGGQVVYILDQVRALEEELLHKIELQGLDVKPQILVVTRLIPDAKGTTCNQELEPVTHTKHSNILRVPFYTDKGMLHQWVSRFDIYPYLERFSQDATAKILELMEDKPDLIIGNYTDGNLVSSLMASKLGVTQATIAHALEKTKYEDSDAKWMAFDEKYHFSCQFTADIISMNAADFIITSTYQEIAGSKQKPGQYETHTAFTMPGLCRAVSGINVFDPKFNIAAPGADQSVYFPSTEKEQRLIAFHPAIEELLFSKDDNEEHIGFLEDMKKPIIFSMARLDKVKNLSGLVEWYARNKRLRSLVNLVVVGGFFNPAKSKDREETEEIKKMHFLMKEYNLKGQFRWIAAQTDRYRNSELYRCISDSKGAFVQPALYEAFGLTVIEAMNCGLPTFATNQGGPAEIIVDEVSGFHIDPYNGDESSDKIADFFEKCKIDSEHWNRMSKAGLQRINECYTWKIYAKKVLNMGSIYGFWKRLNKEQKLAKERYNHMFYNLQFRNLAKQVPIPSERPQDPTQMPKPSAPAPSRRPAAKARPKKVSEHGIVGAPLTLLTAAATPKIKDHPTTSGEGVSARTATSEQSGGGGGLFGLRWLVSIISFLCAIHYLLKNLDRLFTREQ

>GmSUS5

MATDRLTRVHSLRERLDETLTANRNEILALLSRIEAKGKGILQHHQVIAEFEEIPEENRQKLTDGAFGEVLRSTQEAIVLPPWVALAVRPRPGVWEYLRVNVHALVVEELQPAEYLHFKEELVDGSSNGNFVLELDFEPFNAAFPRPTLNKSIGNGVQFLNRHLSAKLFHDKESLHPLLEFLRLHSVKGKTLMLNDRIQNPDALQHVLRKAEEYLGTVPPETPYSEFEHKFQEIGLERGWGDNAERVLESIQLLLDLLEAPDPCTLETFLGRIPMVFNVVILSPHGYFAQDNVLGYPDTGGQVVYILDQVRALENEMLHRIKQQGLDIVPRILIITRLLPDAVGTTCGQRLEKVFGTEHSHILRVPFRTEKGIVRKWISRFEVWPYLETYTEDVAHELAKELQGKPDLIVGNYSDGNIVASLLAHKLGVTQCTIAHALEKTKYPESDIYWKKLEERYHFSCQFTADLFAMNHTDFIITSTFQEIAGSKDTVGQYESHTAFTLPGLYRVVHGIDVFDPKFNIVSPGADQTIYFPHTETSRRLTSFHPEIEELLYSSVENEEHICVLKDRSKPIIFTMARLDRVKNITGLVEWYGKNAKLRELVNLVVVAGDRRKESKDLEEKAEMKKMYGLIETYKLNGQFRWISSQMNRVRNGELYRVICDTRGAFVQPAVYEAFGLTVVEAMTCGLPTFATCNGGPAEIIVHGKSGFHIDPYHGDRAADLLVDFFEKCKLDPTHWDKISKAGLQRIEEKYTWQIYSQRLLTLTGVYGFWKHVSNLDRRESRRYLEMFYALKYRKLAESVPLAAE

>GmSUS6

MASAPALKRTDSVVDNMPDALRQSRYHMKRCFAKYLGKGRRIMKLHHLMEEMELVIDDKSERSQVLEGILGFILSSTQEAVVDPPYVAFAIRPYPGVWEFVKVSSEDLSVEAITPTDYLKFKERVHDEKWATDENSFEADFGAFDFQIPQLTLSSSIGNGLQFTSKFLTSKLTGKLEKTQAIVDYLLTLNHQGESLMINESLNSSAKLQMALVVADAFLSGLPKDTAYQNFELRFKEWGFERGWGDTAGRVKETMRTLSEVLQAPDPVNLEKFLSSLPIIFNVVIFSVHGYFGQADVLGLPDTGGQVVYILDQVKSLEAELLLRIKQQGLNVKPQILVVTRLIPDARGTKCHQELEPISDTKHSHILRVPFQTDKGILHQWISRFDIYPYLERFTQDATAKILEFMEGKPDLVIGNYTDGNLVASLMARKLGITQGTIAHALEKTKYEDSDVKWKELDPKYHFSCQFMADTVAMNASDFIITSTYQEIAGSKDRPGQYESHAAFTLPGLCRVVSGINVFDPKFNIAAPGADQSVYFPYTEKEKRLSQFHPAIEDLLFSKVDNIEHIGYLADRRKPIIFSMARLDVVKNLTGLVEWYGKNKRLRNLVNLVIVGGFFDPSKSKDREEMAEIKNMHDLIDKYQLKGQFRWIAAQTNRYRNGELYRCIADTRGAFVQPALYEAFGLTVIEAMNCGLPTFATNQGGPAEIIVDGVSGFHIDPLNGDESSNKIADFFEKCKMNQSQWNVISAAGLQRINECYTWKIYANKMVNMGNIYTFWRQVNKEQKEAKQRYIQMFYNLIFKNLVKTVPVPSDEPQQPVGKQPSLKSRSTGRSHSRLQRLFGN

>GmSUS7

MANHPLTHSHSFRERFDETLTGHRNEILALLSRLEAKGKGILQHHQVVAEFEEIPEESRKKLQGGVFGEVLRSTQEAIVLPPFVALAVRPRPGVWEYLRVNVHMLVVDELLPAEYLRFKEELVEGSSNGNFVLELDFEPFNASFPRPTLNKSIGNGVEFLNRHLSAKLFHDKESMQPLLEFLRLHSYKGKTMMLNDKVQSLDSLQHVLRKAEEYLTSVAPETPYSEFENKFREIGLERGWGDIAERVLEMIQLLLDLLEAPDPCTLETFLGRVPMVFNVVILSPHGYFAQDNVLGYPDTGGQVVYILDQVRALENEMLNRIKKQGLDITPRILIITRLLPDAVGTTCGQRLERVYDTEYCDILRVPFRTEKGIVRKWISRFEVWPYLETYTEDVALELAKELQAKPDLIVGNYSDGNIVASLLAHKLGVTQCTIAHALEKTKYPESDIYWKKFEEKYHFSCQFTADLFAMNHTDFIITSTFQEIAGSKDTVGQYESHTAFTLPGLYRVVHGIDPFDPKFNIVSPGADMSIYFPYTETERRLTEFHPDIEELLYSSVENEEHICVLKDRNKPIIFTMARLDRVKNITGLVEWYGKNARLRELVNLVVVAGDRRKESKDLEEKAEMKKMYGLIETYKLNGQFRWISSQMNRVRNGELYRVICDTRGAFVQPAVYEAFGLTVVEAMTCGLPTFATCNGGPAEIIVHGKSGYHIDPYHGDRAAEILVEFFEKSKADPSHWDKISQGGLKRIHEKYTWQIYSDRLLTLTGVYGFWKHVTNLERRESKRYLEMFYALKYRKLAESVPLAIEE

>GmSUS8

MPATSVRERVLDTLSRYRNEFISLLSRYVAGGKGILQPHDLLYEVEKLLEEDEGMQKLKDSPFVKELESAKEAIVLPPFVSIALRPRPGVWEYVRVDAFELSVDNLSVAEYLRLKEELVDGQCTDKYVLELDFEPFNVTLPRPTRSSSIGDGVQFLNRHLSSFMFRSKESLEPLLAFLRTHRYDGHAMMLNDRIYNLSKLQSSLAKAEELLSRLLPNAPYSDFEYELQGLGFERGWGDTAERVSEMVHLLLEILQAPDPNTLESFLGRIPMVFNVVVVSPHGYFGQANILGLPDTGGQLVYILDQVRALENEMLIKIQKQGLDVSPKILIVTRLIPEAKGTTCNQRLERVSGTEHSYILRVPFRTKNGILRKWISRFDMWPYLETFAEDASHEIAGELQGIPDLIIGNCSDGNLVATLLSYKLGITQCNIAHALEKTKHPDSDIYWKKYEDKYHFTCQFTADLIAMNNADFIITSTYQEIAGSKNNVGQYESYTAFTLPGLYRVVHGIDVFDPKFNIVSPGADMCIYFPYSDRERRLTSLHGSIEKLVYGAEQNEEHIGLLNDRSKPIIFSMARIDPVKNITGLVECFGKSSKLRELVNLVVVGGYIDVQKSTDIEEMREIEKMHNLIEEYNLHGQFRWIKAQMNRARNGELYRYIADVKGAFVQPALYEAFGLTVVEAMTCGLPTFATCHGGPAEIIEHGVSGFHIEPHHPDHVAAILINFFEQCQSDPGYWNKISDAGLRRIHERYTWKIYSERLLTLAGVYGFWKHVSKLEKRETRRYLEMFYILKFRDLVKSIPLAVN

>GmSUS9

MASTAPNSALKRSDSITDSMPEALKQSRFHMKRCFARFVASGKRLMKQQHVMDDVEKTVEDKAERKKFLDGMLGYIFSCTQEAAVVPPYVAFAVRPNPGFWEYIKVNADDLQVEGIEAVEYLKYKEMIFDEKWANDENALELDFGAIDFSTPQMVLSSSIGNGLNFTTKILTSRLSGSSQSINPLLDYLLSLNYQGENLMIKDTLNTMPKLQQALKVAEAYVSALNKDTAYQKFEDRFKEWGFDKGWGNTAGRVKETMKLLSEVLESADPVKLESLFSRLPNMFNIVILSIHGYFGQADVLGLPDTGGQVVYILDQVRALEEELLHKIELQGLDVKPQILVVTRLIPDAKGTTCNQELEPVTNTKHSNILRVPFYTDKGMLRQWVSRFDIYPYLERFSQDATAKIFDLMEDKPDLIIGNYTDGNLVSSLMASKLGVTQATIAHALEKTKYEDSDAKWMAFDEKYHFSCQFTADIISMNAADFIITSTYQEIAGSKQKPGQYETHTAFTMPGLCRAVSGINVFDPKFNIAAPGADQSVYFPSTAKEQRLTSFHPAIEELLYSKDDNEEHIGLLEDMKKPIIFSMARLDKVKNLSGLVEWYARNKRLRSLVNLVVVGGFFNPAKSKDREETEEIKKMHFLMKEYNLKGQFRWIAAQTDRYRNSELYRCISDTKGAFVQPALYEAFGLTVIEAMNCGLPTFATNQGGPAEIIVDGVSGFHIDPYNGDESSDKIADFFEKCKTDSQHWNRMSKAGLQRINECYTWKIYAKKVLNMGSIYGFWRRLNREQKLAKERYIHMFYNLQFRNLAKQVPIPSETPQDPTQMPKPSAPAPSRRSAAKARPKKVSEHWIVGAPLTLLTAAATPKIKDHPTPSGEGVSEGTATSEQSGGGGLFGLRWLVPIIAFVCAIHYFLKNLDRLFTREQ

>GmSUS10

MATDRLTRVHSLRERLDETLTANRNEILALLSRIEAKGKGILQHHQVIAEFEEIPEENRQKLTDGAFGEVLRSTQEAIVLPPWVALAVRPRPGVWEYLKVNVHALVVEELQPAEYLHFKEELVDGSSNGNFVLELDFEPFNAAFPRPTLNKSIGNGVQFLNRHLSAKLFHDKESLHPLLEFLRLHSVKGKTLMLNDRIQNPDALQHVLRKAEEYLGTVPPETPYSEFEHKFQEIGLERGWGDNAERVLESIQLLLDLLEAPDPCTLETFLGRIPMVFNVVILSPHGYFAQDNVLGYPDTGGQVVYILDQVRALENEMLHRIKQQGLDIVPRILIITRLLPDAIGTTCGQRLEKVFGTEHSHILRVPFRTEKGIVRQWISRFEVWPYLETYTEDVAHELAKELQGKPDLIVGNYSDGNIVASLLAHKLGVTQCTIAHALEKTKYPESDIYWKKLEERYHFSCQFTADLFAMNHTDFIITSTFQEIAGSKDTVGQYESHTAFTLPGLYRVVHGIDVFDPKFNIVSPGADQTIYFPPTETSRRLTSFHPEIEELLYSSVENEEHICVLKDRSKPIIFTMARLDRVKNITGLVEWYGKNAKLRELVNLVVVAGDRRKESKDLEEKAEMKKMYGLIETYKLNGQFRWISSQMNRVRNGELYRVICDTKGAFVQPAIYEAFGLTVVEAMTCGLPTFATCNGGPAEIIVHGKSGFHIDPYHGDRAADLLVDFFEKCKLDPTHWETISKAGLQRIEEKYTWQIYSQRLLTLTGVYGFWKHVSNLDRRESRRYLEMFYALKYRKLAESVPLAVE

>GmSUS11

MSTQPKLGRIPSIRDRVEDTLSAHRNELISLLSRYVAQGRGILQPHNLIDELDNIPGDDQAIVDLKNGPFGEIVKSAKEAIVLPPFVAIAVRPRPGVWEYVRVNVSELSVEQLSVSEYLSFKEELVDGKINDNFVLELDFEPFNATFPRPTRSASIGNGVQFLNRHLSSIMFRNKDSLQPLLDFLRAHKYKGHALMLNDRIQTISKLQSALAKAEDYLSKLAHDTLYSEFEYVLQGMGFERGWGDTAERVLEMMHLLLDILQAPDPSTLETFLGRVPMVFNVAILSPHGYFGQANVLGLPDTGGQVVYILDQVRALENEMLLRIKKQGLDFTPRILIVTRLIPDAKGTTCNQRLERVSGTDHTHILRVPFRSESGTLRKWISRFDVWPYLETYAEDVASEIAAELQGYPDFIIGNYSDGNLVASLLAYKMGVTQCTIAHALEKTKYPDSDLYWKKFEDKYHFSCQFTADLIAMNNADFIITSTYQEIAGTKNTVGQYESHAGFTLPGLYRVVHGIDVFDPKFNIVSPGADMSIYFPYSEKQNRLTALHGSIEQLLFAPEQTDEYIGLLKDKSKPIIFSMARLDRVKNITGLVESFGKNSKLRELVNLVIVAGYIDVKKSSDREEIAEIEKMHELMKKYNLVGDFRWIAAQTNRARNGELYRYIADTQGAFVQPAFYEAFGLTVVEAMNCGLPTFATCHGGPAEIIEHGISGFHIDPYHPDQASQLLVEFFQKSKEDPSHWKKISDGGLQRIYERYTWKIYSERLMTLAGVYSFWKYVSKLERRETRRYLEMFYILKFRDLANSVPLAKDDAS

>VvSUS1

MASKPTLKRADSMAENMPDALRQSRYHMKRCFARYIGKGKRLMKLNHLMDEMEAVIDDKNERTQVLEGVL

GFILCSTQEAVAIPPHVIFSIRSNPGFWEYVKVSSDDLSVEAITAADYLKFKEMVFDENWAKDDNALELN

FSAFDFPMPRLTLSSSIGNGVSLVSKFMTSKLNGNSQSAQPLVDYLLSLNHQGEKLMITNTLNTPTKLQM

ALIVAEVFVSALPKDTPYPSFELRFKEWGFEKGWGNTAERVKETMRSLSEALEAPDPMNMEKFLSRLPTI

FNVVIFSPHGYFGQSDVLGLPDTGGQVVYILDQVRALEEELLLRIKLQGLNVKPQILVVTRLIPDARGTK

CNQEWEPIDNTKHSTILRIPFRTEKGILNQWVSRFDIYPYLERFTQASIITSMDATAKIIEHMEGKPDLI

IGNYTDGNLVASLMATKLGITQGTIAHALEKTKYEDSDVKWKELEPKYHFSCQFTADTISMNAADFIITS

TYQEIAGSKDRPGQYESHTSFTLPGLCRVVSGINLFDPKFNIAAPGADQSVYFPYMERHKRLTSFQPAIE

ELLYSKQDNNEHIGFLADRKKPIIFSMARLDIVKNITGLTEWFGNNKRLRSLVNLVIVAGFFDPSKSKDR

EEMAEIKKMHTLIEKYQLKGQIRWIAAQNDRRRNGELYRCIADTKGAFVQPAIYEAFGLTVIEAMNCGLP

TFATNQGGPAEIIVDGVSGFHIDPNIGDESSNKIADFFEKCRDDSDHWNKISKAGLQRINECYTWKIYAN

KVLNMGCVFSFWRQLNTEHKQAKQKYIHMFYTLQFRNLVKNIPIPASEVQPPVSRAITKVPPTQRHVTYP

TN

>VvSUS2

MPHRYDGQSMRERFQETLSAHRNELVSLFTGYVAQGKGILQPHHMIDELDKVVGKDEGMQKLRDSPFSKVLKSAQEAIVLPPFVAIAIRPRPGVWEYIRV
NVYELNVDQLSVSEYLQFKEELVDGQIKGNYVLELDFEPFNATFPRPTRSSSIGNGVQFLNRHLSSIMFRNKESLEPLLDFLRAHKHDGHVMMLNDRIQN
ISRLQSALARAEEYLSKLPPLTPYSEFEFELQGMGFEKGWGDTAQRVSEMVHLLLEILQAPDPSTLETFLGRIPMVFNVVIVSPHGYFGQANVLGLPDTG
GQIVYILDQVRALENEMLLRIQKQGLDVIPKILIVTRLIPDAKGTTCNQRLERISGTEHTHILRVPFRTENGILRKWISRFDVWPYLETFAEDASNEIAA
ELQGVPDLIIGNYSDGNLVASLLSYKMGITQCNIAHALEKTKYPESDIYWRKFEDKYHFSSQFTADLIAMNNADFIITSTYQEIAGSKNHVGQYESHTAF
TLPGLYRVVHGIDVFDPKFNIVSPGADMSIYFSYSEKERRLTALHDSIESLLYDSEQNDDHIGMLSDRSKPIIFSMARLDRVKNITGLVECFGKSSKLRE
LVNLVVVAGYIDVTKSRDREETKEIEKMHDLIKKYNLHGQFRWIPAQMNRARNGELYRYIADTKGAFVQPAFYEAFGLTVVEAMTCGLPTFATCHGGPAE
IIEDGLSGFHIDPYHPDQVALRLADFFERCQKDPSYWDEISNGGLKRIYERYTWKIYTERLLTLAGVYGFWKHVSKLERRETRRYLEMFYILKLKDLATS
IPLAVDEH

>VvSUS3

MVTPKLGRSPSIRDRVEDTLSAHRNELVALLSRYVAQGNGILQPHHLIDELDNIVGDDVGRQKLSDGPFGQILKSTQEAIILPPFVAIAVRPRPGVWEYV
RVNVHELSVDQLSVSEYLRFKEELVDGMFNDYYVLELDFEPFNASFPRPNRSSSIGNGVQFLNRHLSSIMFRNKESLEPLLDFLRVHKYKGQVIMLNDRI
QSISRLQSALVKADDHLTKLPPETPFGEFEYEFQGMGFERGWGDTAQRVLEMIHLLLDILQAPDPSTLETFLGRIPMVFNVVILSPHGYFGQANVLGLPD
TGGQVVYILDQVRALENEMLLRMQKQGLDVTPRILIVTRLIPDAKGTTCNQRLERVSGTEHSHILRVPFRTDKGILRKWISRFDVWPYLETFAEDAASEI
AAELQGVPELIIGNYSDGNLVASLLASKLGVTQCTIAHALEKTKYPDSDIYWKNFDDKYHFSCQFTADLIAMNNADFIITSTYQEIAGSKNTVGQYESHT
AFTLPGLYRVVHGIDVFDPKFNIVSPGADMCIYFPYSDVEKRLTALHGSIEKLLYDPEQNEEHIGMLNDRSKPIIFSMARLDQVKNITGLVECYAKNAKL
REMANLVVVAGYNDVKKSNDREEIVEIEKMHDLMKEYNLHGQFRWMSSQTNRARNGELYRYIADTRGIFVQPAFYEAFGLTVVEAMTCGLPTFATCHGGP
AEIIENGVSGFHIDPYHPDQVATTMVDFFEKCKEDSSHWNKISDAGLQRIYERYTWKIYSERLMTLAGVYGFWKYVSKLSRRETRRYLEMFYTLKFRDLA
KSVPLAIDDQH

>VvSUS4

MADGVLTGVHSLRARVDETLTAHRNEILSFLSRIEGHGKGILQPHQLLAEFEALPEVNRKKLSDGPFGDILKSIQEAIVLPPWIAFAVRPRPGVWEYIRV
NVSALVVEELLVPEYLHFKEELVDGSCNGNFVLELDFEPFTASVPRPTLSKSIGNGVEFLNRHLSAKMFHDKDSMQPLLDFLRTHQYKGKTMMLNDRIQN
LDTLQFVLRKAEEYLSSQAPETPYPEFEHKFQEIGLERGWGDTAERVLEMIHLLLDLLEAPDPCTLEQFLGRIPMVFNVVILSPHGYFAQDNVLGYPDTG
GQVVYILDQVRAMETEMLLRIKQQGLDITPKIIIVTRLLPDAVGTTCNQRIEKVYGTEHSIILRVPFRTEKGIVRKWISRFEVWPYLETYTEDVAKELAT
ELQTKPDFIIGNYSDGNIVASLLAHKLGVTQCTIAHALEKTKYPESDIYWKKLEDKYHFSCQFTADLIAMNHTDFIITSTFQEIAGSKDTVGQYESHTGF
TMPGLYRVVHGIDVFDPKFNIVSPGADMTIYFSYTEEKMRLKALHPEIEELLFSPVENKEHLCVLKDRNKPIIFSMARLDRVKNLTGLVEWYGKNTRLRE
LVNLVVVGGDRRKESKDLEEQSEMKKMHELIETYKLNGQFRWISSQMDRVRNGELYRYIADTKGVFVQPAFYEAFGLTVVEAMTCGLPTFATCNGGPAEI
IVHGKSGFHIDPYHGDKAAELLANFFEKCKADPTHWEKISKAGLKRIEEKYTWKIYSERLLTLAGVYGFWKYVSNLDRRETRRYLEMFYALKYRKLAQSV
PLAVEE

>VvSUS5

MASSSAPVIKQQDIADTMPDALKQSRYHMKRCFARFVGSGRRLMKYRHIMEEIEKSIEDKAERSRVMDGLLGYILNTTQEAAVVPPYVAFAVRPSPGLWE
FVKVSADDLGVDGITSAEYLKFKETIFDENWATDENTLEIDFGAFDYSTPHLTLNSSIGNGLNYVSKFMTSKLSGSSENAKPLVEYLLAMNHQGESLMIN
EMLNTVSKLQTALIVAEVFVSSLPKDTPYQSFEQRLKDWGFEKGWGDSAERVKDTMRTLSEVLQAPDPMKMELLFSRLPNMFNIVVFSPHGYFGQADVLG
LPDTGGQVVYILDQVKALEEELLHRIKQQGLIVKPQILVVTRLIPDARGTKCDQEIEPVLNTKHSHILRVPFRTENGVLRQWVSRFDIYPYLERYAQDAS
AKILAHMECKPDLIIGNYTDGNMVASLMASKLGVTQGTIAHALEKTKYEDSDVKWKELDGKYHFSCQFTADMFAMNATDFIITSTFQEIAGSKDRPGQYE
NHAAFTMPGLCRVVSGINVFDTKFNIAAPGADQSVYFPYMEKQKRLTSFHPAIEELLYSKEDNKEHLGYLSDRKKPIIFSMARLDTVKNITGLTEWYGKN
KRLRSLVNLVVVAGFFDPSKSKDREEIAEIKKMHSLIEKYQLKGQLRWIAAQNDRNRNGELYRCIADTKGAFVQPALYEAFGLTVIEAMNCGLPTFATNQ
GGPAEIIFDGVSGFHIDPSNGDESSDKIADFFEKCKTDSEYWNKISTAGLQRIYECYTWKIYATKVLNMGSTYGFWRQLNKDQKNAKNRYLQLFYNLQFR
KLAKGVPILNEEPREEPQQPAATAITKPQQPAPTEGAKPRPSAPTTAPKPQPAARRPQSGVQRVNEGLDQKQPGLPTRIRAFCPWLWWFFIINISLFLIW
YLLMKL

>ZmSH1

MAAKLTRLHSLRERLGATFSSHPNELIALFSRYVHQGKGMLQRHQLLAEFDALFDSDKEKYAPFEDILRA

AQEAIVLPPWVALAIRPRPGVWDYIRVNVSELAVEELSVSEYLAFKEQLVDGQSNSNFVLELDFEPFNAS

FPRPSMSKSIGNGVQFLNRHLSSKLFQDKESLYPLLNFLKAHNYKGTTMMLNDRIQSLRGLQSSLRKAEE

YLLSVPQDTPYSEFNHRFQELGLEKGWGDTAKRVLDTLHLLLDLLEAPDPANLEKFLGTIPMMFNVVILS

PHGYFAQSNVLGYPDTGGQVVYILDQVRALENEMLLRIKQQGLDITPKILIVTRLLPDAAGTTCGQRLEK

VIGTEHTDIIRVPFRNENGILRKWISRFDVWPYLETYTEDVSSEIMKEMQAKPDLIIGNYSDGNLVATLL

AHKLGVTQCTIAHALEKTKYPNSDIYLDKFDSQYHFSCQFTADLIAMNHTDFIITSTFQEIAGSKDTVGQ

YESHIAFTLPGLYRVVHGIDVFDPKFNIVSPGADMSVYYPYTETDKRLTAFHPEIEELIYSDVENSEHKF

VLKDKKKPIIFSMARLDRVKNMTGLVEMYGKNARLRELANLVIVAGDHGKESKDREEQAEFKKMYSLIDE

YKLKGHIRWISAQMNRVRNGELYRYICDTKGAFVQPAFYEAFGLTVIESMTCGLPTIATCHGGPAEIIVD

GVSGLHIDPYHSDKAADILVNFFDKCKADPSYWDEISQGGLQRIYEKYTWKLYSERLMTLTGVYGFWKYV

SNLERRETRRYIEMFYALKYRSLASQVPLSFD

>ZmSUS1

MGEGAGDRVLSRLHSVRERIGDSLSAHPNELVAVFTRLKNLGKGMLQPHQIIAEYNNAIPEAEREKLKDG

AFEDVLRAAQEAIVIPPWVALAIRPRPGVWEYVRVNVSELAVEELRVPEYLQFKEQLVEEGPNNNFVLEL

DFEPFNASFPRPSLSKSIGNGVQFLNRHLSSKLFHDKESMYPLLNFLRAHNYKGMTMMLNDRIRSLSALQ

GALRKAEEHLSTLQADTPYSEFHHRFQELGLEKGWGDCAKRAQETIHLLLDLLEAPDPSTLEKFLGTIPM

VFNVVILSPHGYFAQANVLGYPDTGGQVVYILDQVRAMENEMLLRIKQCGLDITPKILIVTRLLPDATGT

TCGQRLEKVLGTEHCHILRVPFRTENGIVRKWISRFEVWPYLETYTDDVAHEIAGELQANPDLIIGNYSD

GNLVACLLAHKMGVTHCTIAHALEKTKYPNSDLYWKKFEDHYHFSCQFTTDLIAMNHADFIITSTFQEIA

GNKDTVGQYESHMAFTMPGLYRVVHGIDVFDPKFNIVSPGADLSIYFPYTESHKRLTSLHPEIEELLYSQ

TENTEHKFVLNDRNKPIIFSMARLDRVKNLTGLVELYGRNKRLQELVNLVVVCGDHGNPSKDKEEQAEFK

KMFDLIEQYNLNGHIRWISAQMNRVRNGELYRYICDTKGAFVQPAFYEAFGLTVVEAMTCGLPTFATAYG

GPAEIIVHGVSGYHIDPYQGDKASALLVDFFDKCQAEPSHWSKISQGGLQRIEEKYTWKLYSERLMTLTG

VYGFWKYVSNLERRETRRYLEMLYALKYRTMASTVPLAVEGEPSSK

>ZmSUS3

DRVEDTLHAHRNELVALLSKYVNKGKGILQPHHILDALDEVQGSGGRALAEGPFLDVLRSAQEAIVLPPF

VAIAVRPRPGVWEYVRVNVHELSVEQLTVSEYLRFKEELVDGQHNDPYVLELDFEPFNVSVPRPNRSSSI

GNGVQFLNRHLSSIMFRNRDCLEPLLDFLRGHRHKGHVMMLNDRIQSLGRLQSVLTKAEEHLSKLPADTP

YSQFAYKFQEWGLEKGWGDTAGHVLEMIHLLLDIIQAPDPSTLEKFLGRIPMIFNVVVVSPHGYFGQANV

LGLPDTGGQIVYILDQVRALENEMVLRLKKQGLDVSPKILIVTRLIPDAKGTSCNQRLERISGTQHTYIL

RVPFRNENGILKKWISRFDVWPYLETFAEDAAGEIAAELQGTPDFIIGNYSDGNLVASLLSYKMGITQCN

IAHALEKTKYPDSDIFWKNFDEKYHFSCQFTADIIAMNNADFIITSTYQEIAGSKNTVGQYESHTAFTLP

GLYRVVHGIDVFDPKFNIVSPGADMSIYFPHTEKAKRLTSLHGSIENLIYDPEQNDEHIGHLDDRSKPIL

FSMARLDRVKNITGLVEAFAKCAKLRELVNLVVVAGYNDVNKSKDREEIAEIEKMHELIKTHNLFGQFRW

ISAQTNRARNGELYRYIADTHGAFVQPALYEAFGLTVVEAMTCGLPTFATLHGGPAEIIEHGVSGFHIDP

YHPEQAVNLMADFFDRCKQDPDHWVNISGAGLQRIYEKYTWKIYSERLMTLAGVYGFWKYVSKLERLETR

RYLEMFYILKFRELAKTVPLAIDQPQ

>OsSUS1

MAAKLARLHSLRERLGATFSSHPNELIALFSRYVNQGKGMLQRHQLLAEFDALIEADKEKYAPFEDILRA

AQEAIVLPPWVALAIRPRPGVWDYIRVNVSELAVEELSVSEYLAFKEQLVDGHTNSNFVLELDFEPFNAS

FPRPSMSKSIGNGVQFLNRHLSSKLFQDKESLYPLLNFLKAHNHKGTTMMLNDRIQSLRGLQSSLRKAEE

YLMGIPQDTPYSEFNHRFQELGLEKGWGDCAKRVLDTIHLLLDLLEAPDPANLEKFLGTIPMMFNVVILS

PHGYFAQSNVLGYPDTGGQVVYILDQVRALENEMLLRIKQQGLDITPKILIVTRLLPDAVGTTCGQRVEK

VIGTEHTDILRVPFRSENGILRKWISRFDVWPFLETYTEDVANEIMREMQAKPDLIIGNYSDGNLVATLL

AHKLGVTQCTIAHALEKTKYPNSDIYLDKFDSQYHFSCQFTADLIAMNHTDFIITSTFQEIAGSKDTVGQ

YESHIAFTLPGLYRVVHGIDVFDPKFNIVSPGADMSVYFPYTEADKRLTAFHPEIEELLYSEVENDEHKF

VLKDKNKPIIFSMARLDRVKNMTGLVEMYGKNAHLRDLANLVIVCGDHGNQSKDREEQAEFKKMYGLIDQ

YKLKGHIRWISAQMNRVRNGELYRYICDTKGVFVQPAFYEAFGLTVIEAMTCGLPTIATCHGGPAEIIVD

GVSGLHIDPYHSDKAADILVNFFEKCKQDSTYWDNISQGGLQRIYEKYTWKLYSERLMTLTGVYGFWKYV

SNLERRETRRYIEMFYALKYRSLASAVPLAVDGESTSK

>OsSUS2

MGEAAGDRVLSRLHSVRERIGDSLSAHPNELVAVFTRLVNLGKGMLQAHQIIAEYNNAISEADREKLKDG

AFEDVLRSAQEGIVISPWVALAIRPRPGVWEYVRVNVSELAVELLTVPEYLQFKEQLVEEGTNNNFVLEL

DFEPFNASFPRPSLSKSIGNGVQFLNRHLSSKLFHDKESMYPLLNFLRAHNYKGMTMMLNDRIRSLSALQ

GALRKAEEHLSGLSADTPYSEFHHRFQELGLEKGWGDCAKRSQETIHLLLDLLEAPDPSTLEKFLGTIPM

VFNVVIMSPHGYFAQANVLGYPDTGGQVVYILDQVRAMENEMLLRIKQQGLNITPRILIVTRLLPDATGT

TCGQRLEKVLGTEHTHILRVPFRTENGIVRKWISRFEVWPYLETFTDDVAHEIAGELQANPDLIIGNYSD

GNLVACLLAHKMGVTHCTIAHALEKTKYPNSDLYWKKFEDHYHFSCQFTTDLIAMNHADFIITSTFQEIA

GNKDTVGQYESHMAFTMPGLYRVVHGIDVFDPKFNIVSPGADMSIYFPYSESRKRLTSLHPEIEELLYSE

VDNNEHKFMLKDRNKPIIFSMARLDRVKNLTGLVELYGRNPRLQELVNLVVVCGDHGNPSKDKEEQAEFK

KMFDLIEQYNLNGHIRWISAQMNRVRNGELYRYICDTKGAFVQPAFYEAFGLTVVESMTCGLPTFATAYG

GPAEIIVNGVSGFHIDPYQGDKASALLVEFFEKCQEDPSHWTKISQGGLQRIEEKYTWKLYSERLMTLTG

VYGFWKYVSNLERRETRRYLEMLYALKYRTMASTVPLAVEGEPSNK

>OsSUS3

MGETTGERALNRLHSMRERIGDSLSAHTNELVAVFSRLVNQGKGMLQPHQIIAEYNAAIPEGEREKLKDS

ALEDVLRGAQEAIVIPPWIALAIRPRPGVWEYLRINVSQLGVEELSVPEYLQFKEQLVDGSTQNNFVLEL

DFEPFNASFPRPSLSKSIGNGVQFLNRHLSSKLFHDKESMYPLLNFLRAHNYKGMTMMLNDRIRSLDALQ

GALRKAEKHLAGITADTPYSEFHHRFQELGLEKGWGDCAQRVRETIHLLLDLLEAPEPSALEKFLGTIPM

VFNVVILSPHGYFAQANVLGYPDTGGQVVYILDQVRAMENEMLLRIKQQGLNITPRILIVTRLLPDAHGT

TCGQRLEKVLGTEHTHILRVPFRTENGTVRKWISRFEVWPYLETYTDDVAHEISGELQATPDLIIGNYSD

GNLVRCLLAHKLGVTHCTIAHALEKTKYPNSDLYWKKFEDHYHFSCQFTADLIAMNHADFIITSTFQEIA

GNKETVGQYESHMAFTMPGLYRVVHGIDVFDPKFNIVSPGADMSIYFPFTESQKRLTSLHLEIEELLFSD

VENTEHKFVLKDKKKPIIFSMARLDHVKNLTGLVELYGRNPRLQELVNLVVVCGDHGKESKDKEEQAEFK

KMFNLIEQYNLNGHIRWISAQMNRVRNGELYRYICDMRGAFVQPALYEAFGLTVIEAMTCGLPTFATAYG

GPAEIIVHGVSGYHIDPYQNDKASALLVEFFEKCQEDPNHWIKISQGGLQRIEEKYTWKLYSERLMTLSG

VYGFWKYVTNLDRRETRRYLEMLYALKYRKMATTVPLAIEGEASTK

>OsSUS4

MSGPKLDRTPSIRDRVEDTLHAHRNELVALLSKYVSQGKGILQPHHILDALDEVQSSGGRALVEGPFLDV

LRSAQEAIVLPPFVAIAVRPRPGVWEYVRVNVHELSVEQLTVSEYLRFKEELVDGQYNDPYILELDFEPF

NASVPRPNRSSSIGNGVQFLNRHLSSIMFRNKDCLEPLLDFLRGHRHKGHVMMLNDRIQSLGRLQSVLTK

AEEHLSKLPADTPYSQFAYKFQEWGLEKGWGDTAGYVLEMIHLLLDVLQAPDPSTLETFLGRIPMIFNVV

VVSPHGYFGQANVLGLPDTGGQIVYILDQVRALENEMVLRLKKQGLDFTPKILIVTRLIPEAKGTSCNQR

LERISGTQHTYILRVPFRNENGILRKWISRFDVWPYLEKFAEDAAGEIAAELQGTPDFIIGNYSDGNLVA

SLLSYKMGITQCNIAHALEKTKYPDSDIYWTKYDEKYHFSCQFTADIIAMNNADFIITSTYQEIAGSKNT

VGQYESHTAFTLPGLYRIVHGIDVFDPKFNIVSPGADMSIYFPYTEKAKRLTSLHGSLENLISDPEQNDE

HIGHLDDRSKPILFSMARLDRVKNITGLVEAYAKNARLRELVNLVVVAGYNDVKKSKDREEIAEIEKMHE

LIKTYNLFGQFRWISAQTNRARNGELYRYIADTHGAFVQPAFYEAFGLTVVEAMTCGLPTFATVHGGPAE

IIEHGISGFHIDPYHPDQAANLIADFFEQCKQDPNHWVEVSNRGLQRIYEKYTWKIYSERLMTLAGVYGF

WKYVSKLERRETRRYLEMFYILKFRELAKTVPLAVDEAH

>OsSUS5

MASKLSFKRMDSIAETMPDALRQSRYQMKRCFQRYVSKGKRLLKNQQLMEELEKSLDDKVEKEKLVEGFL

GYIICSTQEAVVLPPFVAFAVRMNPGIWEYVKVHSDDLSVEGITPSEYLKFKETLYDEKWAKDDNSLEVD

FGALDLSTPHLTLPSSIGNGLQFVSKFMSSKLGGKPESMKPLLDYLLTLNYRGEKLMINDTIDTVSKLQT

ALLLAEVFVSGLPKYTPYLKFEQRFQEWGLERGWGDTAERCKETLNCLSEVLQAPDPTNMEKFFSRVPSI

FNIVIFSIHGYFGQEKVLGLPDTGGQVVYILDQVRAMEEELLQRIKQQGLHVTPKILVLTRLIPDAKGTK

CNVELEPVENTKYSHILRVPFKTEDGKDLRQWVSRFDIYPYLERYAQDSCAKILDILEGKPDLIIGNYTD

GNLVASLLSNKLCVTQGTIAHALEKTKYEDSDVKWREMDQKYHFSCQFTADMISMNTSDFIITSTYQEIA

GSKEKPGQYEHHYAFTMPGLCRYATGINVFDPKFNIAAPGADQSIYFPFTQKQKRLTDLHPQIDELLYSK

DDTDEHIGYLADRNKPIIFSMARLDKVKNITGLVEWYGQNKKLRDLVNLVVVAGLLDASQSKDREEIEEI

NKMHNLMDRYQLKGQIRWIKAQTDRVRNGELYRCIADTKGAFVQPALYEAFGLTVIEAMNCGLPTFATNQ

GGPAEIIIDGVSGFHVNPINDREAGIKIADFFQKCKEDPSYWNKVSTAGLQRICECYTWKIYATRVLNMG

STYSFWKTLNKEERQAKQRYLQIFYNVQYRNLAKAMARAGDQQARQTTTGVAPSEIVVRPKERKPQTRMQ

RILTRLAGQKPPVSE

>OsSUS6

MAVGLRRSDSIADMMPEALRQSRYQMKRCFQRYVSQGKRLMKRQQLLDELDKSVDDKADKDQLLQGFLGY

VISSTQEAAVLPPFVAFAVRMNPGIWEFVKVHSANLSVEQMTPSDYLKNKEALVDDKWGAYDDDSQLEVD

FGALDLSTPHLTLPSSIGKGAHLVSRFMSSKLTDNKKPLLDYLLALSHRGDKLMINDILDTVDKLQTALL

LAEVYVAGLHPDTNYSEFEQKFQEWGLEKGWGDTAETCKETLSSLSEVLQAPDPINMEKFFSTVPCVFTV

VIFSIHGYFGQEKVLGMPDTGGQVVYILDQVRALEDELLQRIKQQGLNATPKILVLTRLIPEAKGTKCNV

ELEPIENTKHSNILRVPFKTEDGKVLPQWVSRFDIYPYLERYAQDSSVKILEILEGKPDLVIGNYTDGNL

VASLLTSKLGVTQGTIAHALEKTKYEDSDIKWRELDHKYHFSCQFTADMIAMNTSDFIIASTYQEIAGSK

EKPGQYESHYAFTMPGLCRYATGINVFDPKFNIAAPGADQSVYFPFTQKQKRLTDLHPQIEELLYSKEDN

NEHIGHLADRSKPIIFSMARLDKIKNITGLVEWYGQNKRLRDLVNLVIVGGLLDPSQSKDREEIEEINKM

HSLINKYQLVGQIRWIKGQTDRVRNGELYRCIADTKGAFVQPALYEAFGLTVIEAMNCGLPTFATNQGGP

AEIIVDEVSGFHINPLNGKEASDKIADFFQKCKEDLIYWSKMSTAGLQRIYECYTWQIYATKVLNMASIY

GFWRTLDKEERQAKQHYLHMFYNLQFRKLAKNVPTLGEQPAQPTESAEPNRIIPRPKERRTQIRIQRIAT

NLLGPLLPASNFSTDGA

>BD1G20890

MGETTGDQRTLVRLHSVRERICDSLAVHTNELVAVFSRLVNQGKGMLQPHQITAEYDAAIPEAEREKLKDSAFEDLLRGAQEAIVIPPWVALAIRPRPGVWEYVRVNVSELGVEELTVPEYLQFKELLVNGRTDNNFVLELDFGPFNASFPRPSLSKSIGNGVQFLNRHLSSKLFHDKESMSPLLNFLREHNYKGMTMMLNDRIRSISALQAALRMAEKHLSGLPVDTPYSEFHHRFQELGLEKGWGDCAHRVSNTIHLLLDLLEAPDPSALEKFLGTIPMVFNVVILSPHGYFAQANVLGYPDTGGQVTRLLPDAHGTTCGQRLEKVLGTEHTHILRVPFKTEDGIVRKWISRFEVWPYLEAYTDDVAHEIAGELQATPDLIIGNYSDGNLVACLLAHKLGVTHCTIAHALEKTKYPNSDLYWKKFEDHYHFSCQFTADLIAMNHADFIITSTFQEIAGNKDTVGQYESHMAFTMPGLYRVVHGIDVFDPKFNIVSPGADMSIYFPYAEQQKRLTSLHTEIEELLFSDVENAEHKFVLKEKKKPIIFSMARLDRVKNMTGLVEFYGRNPRLQELVNLVVVCGDHGKESKDKEEQAEFKKMFDLINQYNLKGHIRWISAQMNRVRNGELYRYICDMKGAFVQPAFYEAFGLTVIEAMTCGLPTFATAYGGPAEIIVHGVSGYHIDPYQNDKASELLVEFFEKSQEDPSHWNKISQGGLQRIEEKYTWKLYSERLMTLSGVYGFWKYVSNLDRRETRRYLEMLYALKYRKMASTVPLAVEGETSSK*

>BD1G29570

MASKLSFKRMDSVAESMPDALRQSRYQMKRCFQRYVSKGRRLLKNQQLMEELEKSLDDKVEKEKLVEGFLGYIICSTQEAVVLPPFVAFAVRMNPGIWEYVKVHSDDLSVEGITPSEYLKFKETLYDEKWAKDDNSLEIDFGALDLSTPHLTLPSSIGNGMQFVSKFMSSKLSGKPESMKPLLDYLLALNYRGEKLMVNDTIDTVNKLQTALLLAEVFVSGLPKYTPYLKFEQRFQEWGLEKGWGENAERCKETLNFLSEVLQAPDPINMEKFFSRVPSIFNIVVFSIHGYFGQEKVLGLPDTGGQVVYILDQVRSMEEELLQRIKQQGLNVTPKILVLTRLIPDSKGTKCNVELEPVENTQYSHILRVPFKTEDGKDLRQWVSRFDIYPYLERYAQDASVKILDMLEGKPDLIIGNYTDGNLVASLMSSKLGVTQGTIAHALEKTKYEDSDVKWRELDQKYHFSCQFTADMIAMNTTDFIITSTYQEIAGSKEKPGQYEHHYAFTMPGLCRYATGINVFDPKFNIAAPGADQSVYFPYTQKQKRLTGLHPQIEELLYSKEDTDEHIGYLADRNKPIIFSMARLDKVKNITGLVEWYGQNKKVRDLVNLVVVAGLLNASQSKDREEIDEINKMHNLIDKYQLKGQIRWIKAQTDRVRNGELYRYIADTKGAFVQPALYEAFGLTVIEAMNCGLPTFATNQGGPAEIIVDGVSGFHINPMNGREAGNKIADFFQKCKEDPSYWNKVSTAGLQRIYECYTWKIYATKVLNMGSMYGFWRTLNKEEKVAKQRYIQMFYNLQFRNLVKTVPRVGEQPPRTAASTSTGMVAPNEIVLRPKERKPQNRMQRIMTSLLGPKPPTYEQNSYR*

>BD1G46670

MAAKLTRLHSLRERLGATFSSHPNELIALFSRYVHQGKGMLQRHQLLAEFDALMDGDKEKYAPFEDILRAAQEAIVLPPWVALAIRPRPGVWDYIRVNVSELAVEELTVSEYLAFKEQLVDEHASSNFVLELDFEPFNASFPRPSMSKSIGNGVQFLNRHLSSKLFQDKESLYPLLNFLKAHNHKGTTMMLNDRIQSLRGLQSALRKAEEYLISIPEDTPCSEFNHRFQELGLEKGWGDTAKRVHDTIHLLLDLLEAPDPANLEKFLGTIPMMFNVVILSPHGYFAQSNVLGYPDTGGQVVYILDQVRALENEMLLRIKQQGLDITPKILIVTRLLPDAVGTTCGQRLEKVIGTEHTDILRVPFRTEKGILRKWISRFDVWPFLETYTEDVANELMREMQTKPDLIIGNYSDGNLVATLLAHKLGVTQCTIAHALEKTKYPNSDIYLDKFDSQYHFSCQFTADLIAMNHTDFIITSTFQEIAGSKDSVGQYESHIAFTLPGLYRVVHGIDVFDPKFNIVSPGADMSVYFPYTETDKRLTAFHPEIEELIYSDVENSEHKFVLKDKNKPIIFSMARLDRVKNMTGLVEMYGKNAHLKDLANLVIVAGDHGKESKDREEQAEFKRMYSLIEEYKLKGHIRWISAQMNRVRNGELYRYICDTKGAFVQPAFYEAFGLTVIEAMTCGLPTIATCHGGPAEIIVDGVSGLHIDPYHSDKAADILVNFFEKCKVDPTYWDKISQGGLKRIYEKYTWKLYSERLMTLTGVYGFWKYVSNLERRETRRYLEMFYALKYRSLAAAVPLAVDGDNAGN*

>BD1G60320

MGEAAGDRVLSRLQSVRERIGDSLSAHPNELVAVFTRLVNLGKGMLQPHQIISEYNTAIPEAAREKLKDGAFEDVLRAAQEAIVISPWVALAIRPRPGVWEYIRVNVSELAVEELSVPEYLQFKEQLVEGSNKDFVLELDFEPFNASFPRPSLSKSIGNGVQFLNRHLSSKLFHDKESMYPLLNFLRAHNYKGMTMMMNDRIRSLSALQGALRKAEEHLSGLPADTPYSDFHHRFQELGLEKGWGDCAKRAQETLHLLLDLLEAPDPSTLEKFLGTIPMVFNVVILSPHGYFAQANVLGYPDTGGQVVYILDQVRAMESEMLLRIKQQGLDITPRILIVTRLLPDATGTTCGQRLEKVLGTEHTHILRVPFRTENGIVRKWISRFEVWPYLETFTDDVAHEISGELQANPDLIIGNYSDGNLVACLLAHKMGVTHCTIAHALEKTKYPNSDLYWKKFEDHYHFSCQFTTDLIAMNHADFIITSTFQEIAGNKDTVGQYESHMAFTMPGMYRVVHGIDVFDPKFNIVSPGADMSIYFPYSESQRRLTSLHPEIEELLYSDVDNNEHKYVLKDRNKPIIFSMARLDRVKNLTGLVELYGRNPRLQELVNLVIVCGDHGNPSKDKEEQAEFKKMFDLIEQYNLNGHVRWISAQMNRVRNAELYRYICDTKGAFVQPALYEAFGLTVIEAMTCGLPTFATAYGGPAEIIVNGVSGYHIDPYQGDTASALLVDFFEKCQGDPSHWTKISQGGLQRVEEKYTWKLYSERLMTLTGVYGFWKYVSNLERRETRRYLEMLYALKFRTMASTVPLAVEGEASSK*

>BD1G62957

MAAPKLDRTPSIRDRVEDTLHAHRNELVALLSKYVSQGKGILQPHHILDTLDEVQGSVAHALADEPFLEVMRSAQEAIVLPPFVAIAVRPRPGVWEFVRVNVHELSVDQLSVSEYLRFKEELVDGQHNDPYVLELDFEPFTALIPRPNRSSSIGNGVQFLNRHLSSILFRNRDCLEPLLDFLRRHRHKGHVMMLNDRIQSVGRLQSVLTKAEEHLSKFPADTPYSQFANQFQEWGLEKGWGDTAEHILEMIHLLLDVLQAPDPSTLETFLGRIPMIFNVVIVSPHGYFGQANVLGMPDTGGQIVYILDQVRALENEMVLRLKKQGLDVTPKILIVTRLIPDSKGTTCNQRLERISGTQHTFILRVPFRNENGILRKWISRFDVWPYLEKFAEDAAGEIAAELQGTPDFIIGNYSDGNLVASLLSYKMGITQCNIAHALEKTKYPDSDIYWKKFDEKYHFSCQFTADIIAMNSADFIITSTYQEIAGSKNTVGQYESHTAFTLPGLYRIVHGIDVFDPKFNIVSPGADMSIYFPYTEKARRLTALHGSIESLIYDPEQNDEHIGHLDDRSKPILFSMARLDRVKNITGLVEGYSKNAKLRELVNLVVVAGYNDVNKSKDREEIAEIEKMHELIKTYNLSGQFRWISAQTNRARNGELYRYIADTHGAFVQPALYEAFGLTVVEAMTCGLPTFATLHGGPAEIIEHGVSGFHIDPYHPDQAASLMADFFEQCKQEPDHWVKISDKGLQRIYEKYTWKIYSERLMTLAGVYGFWKYVSKLERRETRRYLEMFYILKFRELVKSVPLALDQAH*

>BD3G60687

MPGGGLKRSDSIANMMPEALRQTRYQMKGCFQRYVSKGRRLMKNQQLMEELERSVDDNLEKTKLEEGFLGYIICSTQEAVVLPPFVSFAVRMNPGIWEYIKVHSADLSVEQVTPADYLKSKETLFDEKWACDDNSLEVDFGALDLSTPRLTLPSSIGNGMQFVSRFMCSKLSGKPEDMKPLLDYLLTLNYRGEKLMISDTLDTVNKLQTALLLAEVFVAGLQRNTPYQKFEQKFQEWGLEKGWGDTAETCRETLNCLSEVLQAPDPFNMEKFFNRVPSVFNIVIFSIHGYFGQEKVLGMPDTGGQVVYILDQVRALEEELLQRIKQQGLNVTPKILVLTRLIPEAKGTKCNVELEPVEHTKHSSIVRVPFKSDDGKDLRHWVSRFDIYPYLERYAQDSSVKILEILEGKPDLVIGNYTDGNLVASLMSSKLGVTQGTIAHALEKTKYEDSDVKWREMDQKYHFSCQFTADMITMNTSDFVVASTYQEIAGSKEKPGQYESHYAFTMPGLCRYATGINVFDPKFNIAAPGADQSVYFPFTQKQKRLTDLHPQIEELLYSKEDNDEHIGYLEDRNKPIIFSMARLDKVKNITGLVEWYGQNKKLRELVNLVIVGGLLEPSQSKDREEIEEINRMHSLMNKYLLKGQIRWIKAQTERVRNGELYRCIADTRGAFVQPALYEAFGLTVIEAMNCGLPTFATNQGGPAEIIVDEVSGFHINPLNGKEASDKIAGFFQKCKEDPTCWNKMSTAGLQRIYECYTWQIYATKVLNMGSMYGFWRTLNKEERQAKLCYLQMFYNLQFRQLVKTVPKLGEQPAQPVGSAMPGRIVPRPKERQVCPLLRNLLRKERGSN*

>BeSUS1

MGEAAGDRVLSRLHSVRERIGDSLSAHPNELVAVFTRLVNLGKGMLQPHQIIAEYNNAIPEAEREKLKDG

AFEDVLRAAQEAIVIPPWVALAIRPRPGVWEYVRVNVSELAVEELRVPEYLQFKEQLVEGSTNNNFVLEL

DFEPFNASFPRPSLSKSIGNGVQFLNRHLSSKLFHDKESMYPLLNFLRAHNYKGMTMMLNDRIRSLSALQ

GALRKAEEHLSGLSADTSYSDFHHRFQELGLEKGWGDCVKRAQETIHLLLDLLEAPDPSTLEKFLGTIPM

VFNVVTLSPHGYFAQANVLGYPDTGGQVVYILDQVRAMENEMLLRIKQQGLNITPRILIVTRLLPDATGT

TCGQRLEKVLGTEHTHILRVPFRTENGIVRKWISRFEVWPYLETFTDDVAHEIAGELQANPDLIIGNYSD

GNLVACLLAHKMGVTHCTIAHALEKTKYPNSDLYWKKFEDHYHFSCQFTTDLIAMNHADFIITSTFQEIA

GNKDTVGQYESHMAFTMPGLYRVVHGIDVFDPKFNIVSPGADLSIYFPYTESHKRLTSLHPEIEELLYSD

VDNHEHKFVLKDRNKPIIFSMARLDRVKNLTGLVELYGRNPRLQEQVNLVVVCGDHGNPSKDKEEQAEFQ

KMFDLIEQYNLNGHIRWISAQMNRVRNGELYRYICDTKGAFVQPAFYEAFGLTVVESMTCGLPTFATAYG

GPAEIIVDGVSGFHIDPYQGDKASALLVEFFEKCQQDPSHWTKISQGGLQRIEEKYTWKLYSERLMTLTG

VYGFWKHVSNLERRETRRYLEMLYALKYRTMASTVPLAVDGEPSSK

>BeSUS3

MGETAGDRVLSRLHSVRERIGDSLSAHPNELVAVFTRLVNLGKGMLQPHQIIAEYNNSIPEAERDKLKDG

AFEDVLRAAQEAIVIPPWVALAIRPRPGVWEYVRVNVSELAVEELRVPEYLQFKEQLVEGSTNNNFVLEL

DFVPFNASFPRPSLSKSIGNGVQFLNRHLSSKLFHDKESMYPLLNFLRAHNYKGMTMMLNDRIRSLSALQ

GALRKAEEHLSGLSADTPYSDFHHRFQELGLEKGWGDCAKRAQETIHLLLDLLEAPDPSTLEKFLGTIPM

VFNVVILSPHGYFAQANVLGYPDTGGQVVYILDQVRAMENEMLLRIKQQGLNIMPRILIVTRLLPDATGT

TCGQRLEKVLGTEHTHILRVPFRTENGIVRKWISRFEVWPYLETFTDDVAHEIAGELQANPDLIIGNYSD

GNLVACLLAHKMGVTHCTIAHALEKTKYPNSDLYWKKFEDHYHFSCQFTTDLIAMNHADFIVTSTFQEIA

GNKDTVGQYESHMAFTMPGLYRVVHSIDVFGPKFDIVSPGADLSIYFPYSESPKRLTSLHPEIEELLYSD

VDNNEHKFVLKDRNKPIIFSMARLDRVKNLTGLVELYGWNPRLQELVNLVVVCGDHGNPSKDKEEQAEFK

KMFDLIEQYNLNGHIRWISAQMNRVRNGELYRYIGDTRGALVQPAFYEAFGLTVVESMTCGLPTFATAYG

GPAEIIVHGVSGFHIDPYQGDKASALLVEFFEKCQQDPTHWTKISQGGLQRIEEKYTWKLYSERLMTLTG

VCGFWKYVSNLERRETRRYLEMLYALKYRKMASTVPLAVDGEPSNK

>BeSUS4

MAAKLTRLHSLRERLGASFSSHPNELIALFSRYVNQGKGMLQRHQLLAEFDALIDADKEKYAPFEDILRA

AQEAIVLPPWVALAIRPRPGVWDYIRVNVSELAVEELSVSEYLAFKEQLVDGHTNSNFVLELDFEPFNAS

FPRPSMSKSIGNGVQFLNRHLSSKLFQDKESLYPLLNFLKAHNHKGKAMMLNDRIQSLRGLQSALRKAEE

YLISIPQDTPCSEFNHRFQELGLEKGWGDTAKRVLDTIHLLLDLLEAPDPANLEKFLGTIPMTFNVVILS

PHGYFAQSNVLGYPDTGGQVVYILDQVRALENEMLLRIKQQGLDVTPKVLIVTRLLPDAVGTTCGQRLEK

VIGTEHTDILRVPFRTENGILRKWISRFDVWPFLETYTEDVANEIMREMQAKPDLIIGNYSDGNLVATLL

AHKLGVTQCTIAHALEKTKYPNSDIYLDKFDSQYHFPCQFTADLIAMNHTDFIITSTFQEIAGSKDTVGQ

YESHIAFTLPGLYRVVHGIDVFDPKFNIVSPGADMSVYFPYTETDKRLTAFHPEIEGLIYSDVENSEHQF

VLKNKNKPIIFSMARLDRVKNMTGLVEMYGKNAHLRDLANLVIVAGDHGKESKDREEQAEFKRMYSLIEE

YKLKGHIRWISAQMNRVCNGELYRYICDTKGVFVQPAFYEAFGLTVIESMTCGLPTIATCHGGPAEIIVD

GVSGLHIDPYHSDKAADILVNFFEKCKEDPTYWDKISQGGLKRIYEKYTWKLYSERLMTLTGVYGFWKYV

SNLERRETRRYLEMFYALKYRSLASAVPLAVDGDSVAK

>BeSUS5

MGEAAGDRVLSRLRSVRERIGDSLSAHPNELVAVFTRLVNLGKGMLQPHQIIAEYNNAIPEAEREKLKDG

AFEDVLRAAQEAIVIPPWVALAIRPRPGVWEYVRVNVSELAVEELRVPEYLQFKEQLVEGSTNNNFVLEL

DFEPFNASFPRPSLSKSIGNGVQFLNRHLSSKLFHDKESMYPLLNFLRAHNYKGMTMMLNDRIRSLSALQ

GALRKAEEHLSGLSADTSYSDFHHRFQELGLEKGWGDCAKRAQETIHLLLDLLEAPDPSTLEKFLGTIPM

VFNVVILSPHGYFAQANVLGYPDTGGQVVYILDQVRAMENEMLLRIKQQGLNITPRILIVTRLLPDATGT

TCGQRLEKVLGTEHTHILRVPFRTENGIVRKWISRFEVWPYLETFTDDVAHEIAGELQANPDLIIGNYSD

GNLVACLLAHKMGVTHCTIAHALEKTKCPNSDLYWKKFEDHYHFSCQFTTDLIAMNHADFIITSTFQEIA

GNKDTVGQYESHMAFTMPGLYRVVHGIDVFDPKFNIVSPGADLSIYFPYTESHKRLTSLHPEIEELLYSD

VDNHEHKFVLKGRNKPIIFSMARLDRVKNLTGLVELYGRNPRLQELVNLVVVCGDHGNPSKDKEEQAEFQ

KMFDLIEQYNLNGHIRWISAQMNRVRNGELYRYICDTKGAFVQPAFYEAFGLTVVESMTCGLPTFATAYG

GPAEIIVDGVSGFHIDPYQGDKASALLVEFFEKCQQDPSHWTKISQGGLQRIEEKYTWKLYSERLMTLTG

VYGFWKYVSNLERRETRRYLEMLYALKYRTMASTVPLAVDGEPSSK

>HV1558277G00010

MASKLSFKRMDSIAESMPDALQQSRYQMKRCFQRYVSKGRRLLKNQQLVEELEKSLDDKAEKEKLVEGFLGYIICSTQEAVVLPPFVAFAVRMNPGIWEYVKVHSDDLSVEGITPSEYLKFKETLYDEKWAKDDNSLEVDFGALDLSTPHLTLPSSIGNGMQFVSKFMSSKLNDKPESMKPLLDYLLTLNHRGEKLMVNDTIDTVDKLQTALLLAEVFVSGLPKFTPYLKFEQRFQEWGLEKGWGENAERCKETLNFLSEVLQAPDPINMEKFFGRVPSIFNIVVFSIHGYFGQEKVLGLPDTGGQVVYILDQVRSMEEELVQRIKQQGLHITPKILVLTRLIPDSKGTKCNVELEPVENTKYSHILRVPFKTEDGKDLRQWVSRFDIYPYLERYTQDASAKILDILEGKPDLIIGNYTDGNLVASLMSSKLGVTQGTIAHALEKTKYENSDAKWRELDQKYHFSCQFTADMIAMNTTDFIITSTYQEIAGSKEKPGQYEHHYAFTMPGLCRFSTGINVFDPKFNIAAPGADQTVYFPYTQKQKRLTGLHPQIEELLYSKVDTDEHIGYLADRSKPIIFSMARLDKVKNITGLVEWYGQNKKVRDLVNLVVVAGLLNAAQSKDREEIDEINKMHNLIDKYQLKGQIRWIKAQTDRVRNGELYRYIADTKGAFVQPALYEAFGLTVIEAMNCGLPTFATNQGGPAEIIVDGISGFHINPTNGREAGTKIADFFQKCKEDPSYWNKVSTAGLQRIYECYTWKIYATKVLNMGSMYSFWRTLNKEERAAKQRYLQMFYNLQYRNLVKTVPRIAEQPPRTTASTSTSTAGAAVVRDEIVVRPKERKPRNRIQRMMTSLLGPKRRANT

>HV1561797G00010

MAAKLTRLHSLRERLGATFSSHPNELIALFSRYVHQGKGMLQRHQLLAEFDALFESDKEKYAPFEDILRAAQEAIVLPPWVALAIRPRPGVWDYIRVNVSELAVEELTVSEYLAFKEQLVDEHASGKFVLELDFEPFNASFPRPSMSKSIGNGVQFLNRHLSSKLFQDKESLYPLLNFLKAHNYKGTTMMLNDRIQSLRGLQSALRKAEEYLVSIPEDTPSSEFNHRFQELGLEKGWGDTAKRVHDTIHLLLDLLEAPDPASLEKFLGTIPMMFNVVILSPHGYFAQSNVLGYPDTGGQVVYILDQVRALENEMLLRIKQQGLDITPKILIVTRLLPDAVGTTCGQRLEKVIGTEHTDILRVPFRTENGILRKWISRFDVWPYLETYTEDVANELMREMQTKPDLIIGNYSDGNLVATLLAHKLGVTQCTIAHALEKTKYPNSDIYLDKFDSQYHFSCQFTADLIAMNHTDFIITSTFQEIAGSKDSVGQYESHIAFTLPDLYRVVHGIDVFDPKFNIVSPGADMTVYFPYTETDKRLTAFHSEIEELLYSDVENDEHKFVLKDRNKPIIFSMARLDRVKNMTGLVEMYGKNAHLKDLANLVIVAGDHGKESKDREEQAEFKRMYSLIEEYKLKGHIRWISAQMNRVRNGELYRYICDTKGAFVQPAFYEAFGLTVIEAMTCGLPTIATCHGGPAEIIVDGVSGLHIDPYHSDKAADILVNFFEKSTADPSYWDKISQGGLKRIYEKYTWKLYSERLMTLTGVYGFWKYVSNLERRETRRYLEMFYALKYRSLAAAVPLAVDGESSGN

>HV2547343G00020

MCVPSPPVLDAGFVSRPLPINRCPSTPRQITTAQPRRSSLVSFPSLAMASAKLSFKRMDSVAESMPDALRQSRYQMKRCFQRYVSRGRRLLKNQQLMEELDRSLDDELEKEKLVEGFLGYIICSTQEAVVLPPFVAFAVRMNPGIWEYVKVHADDLSVEGITPSEYLKFKDTLYDEKWAKDDNSLEVDFGALDLSTPRLTLPSSIGNGMQFVSKFMSSKLNGKPESMKPLLDYLLALNYRGEKLMVNDTIDTVNKLQTALLLAEVFVSGLPKFTPYLKFEQRFQEWGLEKGWGENAERCKETLNFLSEVLQAPDPINMEKFFSRVPSIFNIVVFSIHGYFGQEKVLGLPDTGGQVVYILDQVRSMEEELLQRIKLQGLHITPKILVLTRLIPDSKGTKCNVELEPVENTKYSHILRVPFKTEDGKDLRQWVSRFDIYPYLERYAQDASTKILDMLEGKPDLIIGNYTDGNLVASLMSSKLGVTQGTIAHALEKTKYEDSDVKWRELDQKYHFSCQFTADMFAMNTTDFIITSTYQEIAGSKEKPGQYEHHYAFTMPGLCRFATGINVFDPKFNIAAPGADQSVYFPFTQKQKRLTNLHPQIEELLYSKEDTDEHIGYLADRSKPIIFSMARLDKVKNITGLVEWYGQNKKVRDLVNLVVVAGLLNAAQSKDREEIDEINKMHNLIDKYQLKGQIRWIKAQTDRVRNGELYRYIADSKGAFVQPALYEAFGLTVIEAMNCGLPTFATNQGGPAEIIVDGVSGFHINPMNGREAGTKIADFFQKCKEDPSYWNKMSTAGLQRIYECYTWKIYATKVLNMGSMYGFWRTLNKEERVAKQRYMQMFYNLQYRNLVKTVPRVGEQPPRPAASTGAVAERNQIVARPRERKPQGRVQRMMTSLLGPKPPTYEQNGYR

>HV355646G00010

MGEAAGDRVLSRLHSVRERIGDSLSAHPNELVAVFTRLVNLGNGMLQSHQIIAEYNAAIPEAEREKLKDGAFEDVLRAAQEAIVISPWVALAIRPRPGVWEYVRVNVSELAVEELTVPEYLQFKEQLVEGSNKDFVLELDFEPFNASFPRPSLSKSIGNGVQFLNRHLSSKLFHDKESMNPLLNFLRAHNYKGMTMMLNDRIRSLSALQGALRKAEEHLSGLPADTPYSDFHHSRFQELGLEKGWGDCAKRAQETLHLLLDLLEAPDPSTLEKFLGTIPMVFNVVILSPHGYFAQANVLGYPDTGGQVVYILDQVRAMENEMLLRIKQQGLDITPRILIVTRLLPDATGTTCGQRLEKVLGTEHTHILRVPFRTESGIVRKWISRFEVWPYLETFTEDVAHEISGELQANPDLIIGNYSDGNLVACLLAHKMGVTHCTIAHALEKTKYPNSDLYWKKFEDHYHFSCQFTTDLIAMNHADFIITSTFQEIAGNKDTVGQYESHMAFTMPGMYRVVHGIDVFDPKFNIVSPGADMSIYFPYSESQRRLTSLHPEIEELLYSNVDNNEHKYVLKDRNKPIIFSMARLDRVKNLTGLVELYGKNPRLQELVNLVVVCGDHGNPSKDKEEQAEFKKMFDLIEQYNLNGHVRWISAQMNRVRNAELYRYICDTKGAFVQPAFYEAFGLTVIEAMTCGLPTFATAYGGPAEIIVNGVSGYHIDPYQGDKASALLVEFFEKCEVDPSHWTKISQGGLQRIEEKYTWKLYSERLMTLTGVYGFWKYVSNLERRETRRYLEMLYALKYRTMASTVPLAVEGESSSK

>HV43511G00010

MGETAGERALSRVHSVRERIGDSLSAHTNELVAVFSRLVNQGKGMLQPHQITAEYNAAIPEAEREKLKNTPFEDLLRGAQEAIVIPPWVALAIRPRPGVWEYVRVNVSELGVEELSVPEYLQFKEQLANGSTDNNFVLELDFGPFNASFPRPSLSKSIGNGVQFLNRHLSSKLFHDKESMYPLLNFLRAHNYKGMTMMLNDRIRSLGTLQGALRKAETHLSGLPADTPYSEFHHRFQELGLEKGWGDCAQRASETIHLLLDLLEAPDPSSLEKFLGTIPMVFNVVILSPHGYFAQANVLGYPDTGGQVVYILDQVRAMENEMLLRIKQQGLDITPKILIVTRMLPDAHGTTCGQRLEKVLGTEHTHILRVPFKTEDGIVRKWISRFEVWPYLEAYTDDVAHEIAGELQANPDLIIGNYSDGNLVACLLAHKLGVTHCTIAHALEKTKYPNSDLYWKKFEDHYHFSCQFTADLIAMNHADFIITSTFQEIAGNKDTVGQYESHMAFTMPGLYRVVHGIDVFDPKFNIVSPGADMSIYFPYTEQQKRLTSLHTEIEELLFSDVENAEHKFVLKDKKKPIIFSMARLDRVKNMTGLVEMYGRNPRLQELVNLVVVCGDHGKVSKDKEEQAEFKKMFDLIEKYNLSGHIRWISAQMNRVRNGELYRYICDMKGAFVQPAFYEAFGLTVIEAMTCGLPTFATAYGGPAEIIVNGVSGYHIDPYQNDKASALLVDFFGKCQEDPSHWNKISQGGLQRIEEKYTWKLYSERLMTLSGVYGFWKYVSNLDRRETRRYLEMLYALKYRKMAATVPLAVEGETSGK

>HV49320G00010

MAAPKLDRTPSIRERVEDTLHAHRNELVALLSKYVSKGKGILQPHRILDTLDEVQVSGGSALAEGPFLDVLRSSQEAIVLPPFVAIAVRPRPGVWEYVRVNVHELNVEQLSVSEYLRFKEELVDGQHNNPYVLELDFEPFTALIPRPSRSSSIGNGVQFLNRHLSSILFRNRDCLEPLLDFLREHRHKGHVMMLNDRIQSVGRLQSVLTKAEENLSKLPAETPYSQFANQFQEWGLEKGWGDTAEHVLEMIHLLLDILQAPDPSTLETFLGRIPMIFNVVIVSPHGYFGQANVLGMPDTGGQIVYILDQVRALENEMVLRLKKQGLDVTPKILIVTRLIPDSKGTSCNQRLERISGTQHTYILRVPFRNENGILRKWISRFDMWPYLEKFAEDAAGEISAELQGTPDFIIGNYSDGNLVASLLSYKMGITQCNIAHALEKTKYPDSDIYWKKFDEKYHFSCQFTADIIAMNNADFIITSTYQEIAGSKNTVGQYESHTAFTLPGLYRVVHGIDVFDPKFNIVSPGADMSIYFPFTEKAKRLTALHGSIESLIYDPEQNDEHIGHLDDPSKPILFSMARLDRVKNMTGLVKAYSKNAKLRSLVNLVVVAGYNDVKKSKDREEIAEIEKMHELIKTYNLFGQFRWISAQTNRVRNGELYRYIADTHGAFVQPALYEAFGLTVVEAMTCGLPTFATLHGGPAEIIEHGISGFHIDPYHPDQAATLMADFFGQCKQDPNHWVKISDKGLQRIYEKYTWKIYSERLMTLAGVYGFWKYVSKLERRETRRYLEMFYILKLRELVKSVPLALDETH

>HV66958G00020

MASSSSMPLRRSDSVADMMPEALRQRRYQMKRCFQSYVSKGRRLMKNQQLMEELETSEGDDKVEKARLAEGFLGYVICSTQEAVVLPPLVAFAVRTNPGVWEFIRVHSGDLSVEQITPADYLKCKETLYDEKWARDDNSLEVDFGALDLSTPHLALPSSIGNGMQFISRFMSSKLSGKPESMKPLLDYLLALNYRGEKLMISDSLDTADKLQTALLLAEVFVASLEKSTPYQQFEQKFQEWGLEKGWGDTAETCRETLNFLSEVLQAPDPINMEKFFSRVPSVFNIVIFSIHGYFGQEKVLGLPDTGGQVVYILDQVRALEEELLQRIKRQGLNVTPKILVLTRLIPDAKGTKCNVELEPVEHTKHSSILRVPFKTDDGKDLRQWVSRFDIYPYLERYAKDSSVKILDILEGKPDMVIGNYTDGNLVASLLSSKLGVTQGTIAHALEKTKYEDSDVKWREMDHKYHFSCQFTADMIAMNTSDFIIASTYQEIAGSKDKPGQYESHYAFTMPGLCRYATGVNVFDPKFNIAAPGADQTVYFPFTQKQARLTDLHPQIEELLYSKEDNDEHLGYLGDRSKPIIFSMARLDKVKNITGLVEWYGENKKLRDLVNLVIVGGLLEPSQSNDREEIEEINKMHSLMDKYQLKGQIRWIKAQTERVRNGELYRCIADTRGAFVQPALYEAFGLTVIEAMNCGLPTFATNQGGPAEIIVNEVSGFHINPLNGKESSDKIAAFFQKCKEDPTYWNKMSTAGLQRIYECYTWQIYATKVLNMGSMYGFWRTLNKEERQAKQLYLQMFYNLLFRQLVKTVPKLGEQPAQPTTAPARIAPRPRERRPQTRIQRIATSLLGPVLPTSNFSQDAA

>MA02G22450

MPQRSLTRAHSVRERIGDSLSSHPNELVALFSRFIHQGKGMLQPHQLLAEYAAAFSEADKEKLKDGAFEDVIKAAQEAIVIPPWVALAIRPRPGVWEYVRVNISELAVEELTVPEYLHFKEELVDGSSQNNFVLELDFEPFNASFPRPSLSKSIGNGVQFLNRHLSSKLFQDKESLYPLLNFLRKHNYKGMSMMLNDRIQSLSALRAALRKAEQHLLSIPSDTPYSEFHHRFQELGLEKGWGDKSQRVYENIHLLLDLLEAPDPTTLETFLGTIPMMFNVVILSPHGYFAQANVLGYPDTGGQVVYILDQVRALENEMLLRIKRQGLDITPRILIVSRLLPDAVGTTCGQRLEKVLGTEHTHILRVPFRTDNGIVRKWISRFEVWPYLETYTEDVANELAAELQATPDLIIGNYSDGNLVSTLLAHKLGVTQCTIAHALEKTKYPNSDIYWKKFEDQYHFSCQFTADLIAMNHADFIITSTFQEIAGSKDTVGQYESHTAFTLPGLYRVVHGIDVFDPKFNIVSPGADLSIYFPYTEKHKRLTSLHPEIEELLFNPVDNTEHKGVLNDKKKPIIFSMARLDRVKNLTGLVEFYGRSDRLKELANLVVVCGDHGKESKDLEEQAEFKKMYSLIEKYNLHGHFRWISAQMNRVRNGELYRYIADTKGVFVQPAFYEAFGLTVVESMTCGLPTFATVHGGPGEIIVDGVSGYHIDPYQGDKAAEIVTNFFDKCKEDPSHWDKISLGGLQRIEEKYTWKLYSERLMTLTGVYGFWKYVSNLDRRETRRYLEMFYALKYRNLAVSVPLAVEGEAAVNGAK*

>MA03G01920

MSQRTLTRAHSVRERIGDSLSSHPNELVALFSRFVHQGKGMLQPHQLLAEYGAVFSEADREKLKDGAFEDVIQAAQEAIVIPPWVALAIRPRPGVWEYVRVNISELAVEELTVPEYLQFKEELADGSSQNSNFVLELDFEPFNASFPRPSLSKSIGNGVQFLNRHLSSKLFQDKESLYPLLNFLRKHNYKGMSMMLNDRIQSLSALRAALRKAEQHLLSIPSKTPYSEFNHRFQELGLEKGWGDTARRVYENIHLLLDLLEAPDPTTLENFLGIIPMMFNVVILSPHGYFAQANVLGYPDTGGQVVYILDQVRALENEMLLRIKRQGLHITPRILIVTRLLPDAVGTTCGQRLEKVLGTEHTHILRVPFRTENGIVRKWISRFEVWPYLETYTEDVANELAAELQATPDLIIGNYSDGNLVSTLLAHKLGVTQCTIAHALEKTKYPNSDIYWKKFENQYHFSCQFTADLIAMNHADFIITSTFQEIAGSKDTVGQYESHTAFTLPGLYRVVHGIDVFDPKFNIVSPGADMTIYFPYTEKQKRLTSLHPEIEELLFNPKDNTEHKGVLNDTKKPIIFSMARLDRVKNLTGLVEFYGKNDRLKELVNLVVVGGDHGKESKDREEQAEFKKMYSLIEKYNLHGHIRWISAQMNRVRNGELYRYIADSRGAFVQPAFYEAFGLTVIESMTCGLPTFATVHGGPGEIIVDGVSGFHIDPYQGDKAANIILNFFGKCKEDPTYWDKISQGGLRRIEEKYTWKLYSERLMTLSGVYGFWKYVSNLDRRETRRYLEMFYALKYRNLAESVPLAADGEAAFNGAK*

>MA03G14280

MAAVSLSFKRSDSIAEGMPEALKESRYQMKKCFARYVSKGKRVMKNPQLMEELEKSIDDEAEKAKVMEGFLGYIICSTQEAVVLPPFVAFAVRPHPGIWEYVKVHSVDLSVDGITPCEYLKNKETIYDEKWATDEHALEVDFGALEPSTPLLTLPSSIGKGAQFISRFISAKLNASSESMKPLLDYLLALNHGGQKLMINNTFDTVNKLQTALLLAEVFVSGLPKNTPFQKFEPRFEEWGLEKGWGDTAATVKETLNCLSEVLQAPDPVNLEKFFGRVPSIFNIVILSPHGYFGQADVLGLPDTGGQIVYILDQVKAFEEELLLRIKQQGLTIKPQILVVTRLIPEAKGTKCNQELEPILNTKHSHILRVPFKTETGVVQQWVSRFDVYPYLERYAQACLDILQGKPDLIIGNYTDGNLVASLMATKLGVTQGTIAHALEKTKYEDSDVKWKELEPKYHFSCQFTADMIAMNTTDFIITSTYQEIAGSKDRPGQYESHHAFTLPGLCRFVSGIDVFHPKFNIASPGADQSVYFPYTQKQKRLTSLHPAIEELLYSKTDNEEHTGYLEDRKKPIIFSMARLDTVKNITGLVEWYGKNKKLRGLVNLVVVAGFLDPSKSKDREEISEIKKMRSLIEKYQLKGQMRWIAAQTDRVRNGELYRCIADTKGAFVQPALYEAFGLTVIEAMNCGLPTFATNQGGPAEIIVDGVSGFHIDPTDGEEASGKMADFFERCKDASYWNKISTAGLQRIYECYTWKIYATKVLNMGSIYGLWRQLNKEEQLAKEKYLQLFYNLQFRNLAKTVPIATDQAQQEAKPKPVAIPASQPSQNPIRKLLAICTRKHKGGQ*

>MA06G11150

MPQRTLTRAHSVRERIGDSLSSHPNELVALFSRFINQGKGMLQPHQLLAEYAAAFSEADREKLKDGAFEDVIKAAQEAIVIPPWVALAIRPRPGVWEHVRVNISELAVEELTVPEYLHFKEELVDGSSQNNNFVLELDFEPFNASFPRPSLSKSIGNGVQFLNRHLSSKLFHDKESMYPLLNFLRQHNYKGMSMMLNDRIQSLSALQAALRKAEQHLLSIPSATPYSEFNHRFQELGLEKGWGDTAQRVYENIHLLLDLLEAPDPCTLENFLGTIPMMFNVVILSPHGYFAQANVLGYPDTGGQVVYILDQVRALENEMLLRIKRQGLDITPRILIVTRLLPDAVGTTCGQKLEKVIGTEHTHILRVPFRTENGIVRKWISRFEVWPYLETYTEDVANELAGELQTTPDLIIGNYSDGNLVSTLLAHKLGVTQCTIAHALEKTKYPNSDIYWKKFENQYHFSCQFTADLIAMNHADFIITSTFQEIAGSKDTVGQYESHTAFTLPGLYRVVHGIDVFDPKFNIVSPGADLSIYFPYTEKQKRLTSLHPEIEELLFNPEDNTEHKGVLNDTKKPIIFSMARLDRVKNLTGLVEFYGRNERLKELVNLVVVCGDHGKESKDLEEQAEFKKMYDLIEKYNLNGHIRWISAQMNRVRNGELYRYIADTKGAFIQPAFYEAFGLTVVESMTCGLPTFATVHGGPGEIIVDGVSGFHIDPYQGDKAAEIIVNFFEKCKEDPTHWDKISLGGLKRIEEKYTWKLYSERLMTLSGVYGFWKYVSNLDRRETRRYLEMFYALKYRNLAKSVPLAVDGEAINGSK*

>MA07G20260

MAGRTLTRVLSVKERLSGTLSASPNELLAVFSRYVNQGKGMLQRHQLLAEFEAAFSEDEKEKLKGGVFEDVLRAAQEAIVVPPLVALAIRPRPGVWEYVQVNVNELVVGELSASEYLQFKEKLVNGESESNFVLELDFEPFNASFPRPSLSKSIGNGVQFLNRHLSSKLFVDKESMYPLLEFLRTHSYKGTVMMLNDKLQSPRALQSALRKAEQYLLSIPADTPYSEFNNRFQELGFEKGWGDTVQRVLETMHLLLDLLEAPDPCTLEKFLGTIPMVFNVVILSPHGYFAQANVLGYPDTGGQVVYILDQVRALENEMLLRIKQQGLDITPRILIVTRLLPDAVGTTCGERLEQVDETQHTSILRVPFRNEKGILRKWISRFDVWPYLETYTEDVAKELAEELQATPDLIIGNYSDGNLVASLLAHKLGVTQCTIAHALEKTKYPNSDIYWKKFDDQYHFSCQFTADLFAMNHTDFIITSTFQEIAGSKDTVGQYESHTAFTLPGLYRVVHGINVFDPKFNIVSPGADMSVYFPHVEVDKRLTHFHPEIEELLFSSVENDEHKFVLNDRNKPIIFSMARLDRVKNLTGLVELYGRNARLRELANLVVVAGDHGKESKDIEELAERKKMFGLIEEYNLNGQIRWISAQMDRVRNGELYRYIADTKGAFVQPALYEAFGLTVVEAMTCGLPTFATAYGGPAEIIVHGVSGFHIDPYQKDKAAEILVGFFEKCKEDPTHWDKISQGGLQRIYEKYTWKLYSERLMTLAGVYGFWKHVSNLERRETRRYLEMFYALKYRKLAASVPLAVDAESTVDGQNV*

>MA08G23060

MTTKKLERIPSMRERVEDTLSAYRNDLVSLLSRFVSQGKGMLQPHHLVDALATLGDDGRTKLSEGPFSEVLRSAQEAIVLPPFVAIAIRPRPGVWEYVRVNVYELSVEQLSVSEYLQFKEELVDGRSDDRYTLELDFEPFNASFPRPNRSSSIGNGVLFLNRHLSSIMFRNKDCLEPLLDFLRAHKYKGHVMMLNDRVQSVSRLQSVLAKAEEYLSKLIPETPFSEFAYKLQEMGLEKGWGDTAQHVLEMIHLLLDILQAPDPSTLEMFLGRIPMVFNVVILSPHGYFGQANVLGLPDTGGQVVYILDQVRALENEMLLRIKKQGLDIDPKILIVTRLIPDAKGTTCNQRLERVSGTQHSHILRVPFRTEKGILKKWISRFDVWPYLETFTEDVASEIAAELHGTPDLVIGNYSDGNLVASLLAYKLGITQCNIAHALEKTKYPDSDIYWRKFEDKYHFSCQFTADLIAMNNADFIITSTYQEIAGSKNTVGQYESHTAFTLPGLYRVVHGIDVFDPKFNIVSPGADMSIYFTYSEKGKRLTSLHGSIEKLLYDPEQCDLHIGCLDDRSKPIIFSMARLDKVKNITGLVEWFGKSTKLRELVNLVVVAGYIDVKKSSDREEIQEIEKMHQLISSYNLSGQFRWISAQTNRARNGELYRYIADTGGAFVQPAFYEAFGLTVVEAMTCGLPTFATCHGGPAEIIENGLSGFHIDPYHPDQSAVVMVEFFERCKEDSGYWKKISDGGLRRIQERYTWKIYSERLMTLAGVYGFWKYVSKLERRETRRYLEMFYILKFRDLVKSVPRAVDDDH*

>MA09G21760

MAGAGRTVKRSDSIADALPEALKQSRYQMKRCFARYVSKGRRLMKNQQLMDELESTMDDKVEKSKLMEGFLGLVICWTQEAVVLPPFVAFAVRQHPGIWEYVKVNAEDLFVDEITASEFLKFKETIYDERWANDEDALEVDFGAFDLSTPHLSLPSSIGNGMQFISKFLSSKLSENPKNAKPLLDYLLALNHRGEKLMINGFLDTVSRLQSALILAEVFVSNLPKNMPFEKFEQRFQEWGLEKGWGDTAERVKETVNSLSEVLQCPDPVNIEKFLGRVPAIFNIVIFSPHGYFGQADVLGLPDTGGQVVYILDQVRAFEEELLLRIKRQGLTITPRILVVTRLIPEARGTKCNQELEAILNTKHSHILRVPFRTETGVLHQWVSRFDIYPYLERYARDAAAKVLDILEGKPDLIIGNYTDGNLVASLVASKLGVTQGTIAHALEKTKYEDSDVKWKELDPKYHFSCQFTADMISMNTSDFIITSTYQEIAGSKDRPGQYESHNAFTMPGLCRFASGINVFDPKFNIASPGADQSVYFPHTQKHRRLTSFHPAIEELLYSKQDNDEHIGFLADKRKPIIFSMARLDTVKNITGLVEWYGKNSRLRELVNLVVVAGFLDPSKSKDREEISEIKKMHSLIDKYQLKGQLRWIAAQNDRVRNGELYRCIADTKGAFVQPALYEAFGLTVIEAMNCGLPTFATNQGGPAEIIADGVSGFHIDPTKGDESSNKIADFFAKCREDSSYWNRVSTAGLQRINECYTWKIYATKVLNMGTFYGFWRQLNKEEKQAKQRYVKLFYNLQFRKLAKTVPAVDSTSEAVPVSSKPLTRPSSQITRRQALPLFPEIFA*

>MA10G19100

MSQRTLTRAHSFRERIGDSLSSHPNELVALFSRFIQQGKGMLQPHQLLAEYAAVFSEADKEKLKDGAFEDVIKAAQEAIVIPPRVALAIRPRPGVWEYVRVNISELAVEELTVPEYLQFKEELVDESTQNNNFVLELDFEPFNASFPRPSLSKSIGNGVQFLNRHLSSKLFHDKESMYPLLNFLRKHNYKGMSMMLNDRIQSLSALQAALRKAEQHLLSIASDTPYSEFNHRFQELGLEKGWGDTAQRVYENIHLLLDLLEAPDPCTLENFLGIIPMMFNVVILSPHGYFAQANVLGYPDTGGQVVYILDQVRALENEMLLRIKRQGLDITPRILIVSRLLPDAVGTTCGQRLEKVLGTEHTHILRVPFRTENGIIRKWISRFEVWPYLETYTEDVANELAGELQATPDLIIGNYSDGNLVSTLLAHKLGVTQCTIAHALEKTKYPNSDIYWKKFENQYHFSCQFTADLVAMNHADFIITSTFQEIAGSKDTVGQYESHTAFTLPGLYRVVHGIDVFDPKFNIVSPGADLSIYFPYTEKHKRLTSLHPEIEELLFNPEDNTEHKGVLNDTKKPIIFSMARLDRVKNLTGLVEFYGRNERLKELVNLVVVCGDHGKESKDLEEQAEFKKMYSFIEKYNLHGHIRWISAQMNRVRNGELYRYIADTKGAFVQPAFYEAFGLTVVESMTCGLPTFATVHGGPGEIIVDGVSGFHIDPYQGDKAAEIIVNFFEKCKEDPTCWDKISQGGLKRIEEKYTWKLYSERLMTLSGVYGFWKYVSNLDRRETRRYLEMFYALKYRNLAESVPLAVDGEAAVNGAK*

>SB01G033060

MGEAAGDRVLSRLHSVRERIGDSLSAHPNELVAVFTRLKNLGKGMLQPHQIIAEYNSAIPEAEREKLKDGAFEDVLRAAQEAIVIPPWVALAIRPRPGVWEYVRVNVSELAVEELRVPEYLQFKEQLVEEGPNNNFVLELDFEPFNASFPRPSLSKSIGNGVQFLNRHLSSKLFHDKESMYPLLNFLRAHNYKGMTMMLNDRIRSLSALQGALRKAEEHLSTLQADTPYSEFHHRFQELGLEKGWGDCAKRAQETIHLLLDLLEAPDPSTLEKFLGTIPMVFNVVILSPHGYFAQANVLGYPDTGGQVVYILDQVRAMENEMLLRIKQCGLDITPKILIVTRLLPDATGTTCGQRLEKVLGTEHCHILRVPFRTENGIVRKWISRFEVWPYLETYTDDVAHEIAGELQANPDLIIGNYSDGNLVACLLAHKMGVTHCTIAHALEKTKYPNSDLYWKKFEDHYHFSCQFTTDLIAMNHADFIITSTFQEIAGNKDTVGQYESHMAFTMPGLYRVVHGIDVFDPKFNIVSPGADLSIYFPYTESHKRLTSLHPEIEELLYSQTENTEHKFVLNDRNKPIIFSMARLDRVKNLTGLVELYGRNKRLQELVNLVVVCGDHGNPSKDKEEQAEFKKMFDLIEQYNLNGHIRWISAQMNRVRNGELYRYICDTKGAFVQPAFYEAFGLTVVEAMTCGLPTFATAYGGPAEIIVHGVSGFHIDPYQGDKASALLVDFFEKCQTDSSHWNKISQGGLQRIEEKYTWKLYSERLMTLTGVYGFWKYVSNLERRETRRYLEMLYALKYRTMASTVPLAVEGEPSSK*

>SB01G035890

MSAPKLDRNASIRDRVEDTLHAHRNELVALLSKYVNKGKGILQPHHILDALDEVQGSGVRALAEGPFLDVLRSAQEAIVLPPFVAIAVRPRPGVWEYVRVNVHELSVEQLTVSEYLRFKEDLVDGQHNDPYILELDFEPFNASVPRPNRSSSIGNGVQFLNRHLSSIMFRNRDCLEPLLDFLRGHRHKGHVMMLNDRVQSLGRLQSVLTKAEEYLSKLPAETPYAQFAYKFQEWGLEKGWGDTAEHVLEMVHLLLDIIQAPDPSTLEKFLGRIPMIFNVVVVSPHGYFGQANVLGLPDTGGQIVYILDQVRALENEMVLRLKKQGLDFSPKILIVTRLIPDAKGTSCNQRLERISGTQHTYILRVPFRNENGILKKWISRFDVWPYLETFAEDAAGEIAAELQGTPDFIIGNYSDGNLVASLLSYKMGITQCNIAHALEKTKYPDSDIYWKKFDEKYHFSCQFTADIIAMNNADFIITSTYQEIAGSKNTVGQYESHTAFTLPGLYRVVHGIDVFDPKFNIVSPGADMSIYFPHTEKAKRLTSLHGSIENLLYDPEQNDQHIGHLDDRSKPILFSMARLDRVKNITGLVEAFAKCTKLRELVNLVVVAGYNDVKKSKDREEIAEIEKMHELIKTYNLFGQFRWISAQTNRARNGELYRYIADTHGAFVQPAFYEAFGLTVVEAMTCGLPTFATLHGGPAEIIEHGISGFHIDPYHPEQAANLMADFFERCKQDPNHWVKISEAGLKRIYEKYTWKIYSERLMTLAGVYGFWKYVSKLERRETRRYLEMFYILKFRELAKTVPLAIDQPQ*

>SB04G038410

MMPEALRQSRYHMKRCFQRFVSQGSRLMKQQHLLEELHGGGSADNNKQLAADGFLGHVISCTHEAVVLPPYVALAVRRNPGVWEYITVHSGDLTVQQITPSDYLKRKEILFLYDNSSQLEVNLGALDLSTPRLTLPCSIGNGMHLVSRFLSSRLGGGGGRTKNKALLDYLLALRYYRRRPGDQQQINNKLLISDTLDTVGKLQAALLLAQAFVSEQHPDTPYQQMAHRFQEWGLEKGWGDTAEACGHTLACLAEVLQAPDPASIHRFFSRVPSVFDVVIFSVHGYFGQHKVLGMPDTGGQVVYILDQVRALEEELLQRIKGQGLTFTPNILVLTRLIPEAKGTTCNVELEPIENTRHSSILRVPFKTQDGQDLPHWVSRFDIYPYLERYAQILDILGRKPDLVIGNYTDGNLVAYLVSRKLGVTQGTIAHALEKTKYEDSDVKWREMDHKYHFSCQFTADMIAMNTSDFIIASTYQEIAGSKDKPGQYESHYAFTMPGLCRFATGINVFDPKFNIAAPGADQSVYFPFTLKHKRLTDLHPQIEALVYGKEENDEHIGYLENRRKPVIFSMARLDKVKNITGLVEWYGQDKRLRVLVNLVVVGGLLDPTQSKDREEIEEINKMHSLINKYQLKGQIRWIKAQTDRVRNGELYRCIADTRGAFVQPALYEAFGLTVIEAMNCGLPTFATNQGGPAEIIVDEVSGFHINPLDGKEASNKIANFFQKCNEDPMYWNRMSTAGLQRIYECYTWQIYATKVLNMGSMYGFWRTLDKEEKQAKQQYLQMFYNLHFRKLANAVPKVGEQPEQATAVPLPDRSAPRPKERQVCPLLRNLLKIKWGSN*

>SI001G39400

MDGLMRRSDSIADMMPEALRQSRYHMKRCFQRYVAGGSRLMKKTQLLEELHRSAEDGRIHKDRLAEGFLGYVISSTHEAVVLPPLVNFAVRTNPGIWEYIKVHSADLTVDQITPSQYLKCKEMLYDHQWAQDDNSLEVDFGALDDLSTPRLTLPSSIGNGMHFVSRFMSSKLAGTTMSMKPLLDYLLALTHRGHDLMVNATLDTVSKLQTALLHADVFLAGLHGDTPYQKFEHKFQEWGLERGWGHTAEACRETISCLSEVLQAPDPTNMDSFFSRVPSLFSIVIFSIHGYFGQEKVLGLPDTGGQVVYILDQVRALEDELLQRINQQGLHFTPRILVLTRLIPEAKGTKCNVELEPIHNTRHSTILRVPFKTEDGQDLPHWVSRFDIYPYLERYAEDSCAKILETLQGKPDLVIGNYTDGNLVASLVSRKLGVTQGTIAHALEKTKYEDSDVKWREMDRKYHFSCQFTADMIAMNTSDFIIASTYQEIAGSKEKPGQYESHYAFTMPGRCRFATGINVFDPKFNIAAPGADQSVYFPFTLKQKRLTDLHPQIEELVYSKEDNDEHIGYLEDRSKPVIFSMARLDKVKNITGLVEWYGQNKRLRDLVNLVVVGGLLDPSQSKDREEIEEINKMHSLINKYQLKGQIRWIRAQTDRVRNGELYRCIADTKGAFVQPAFYEAFGLTVIEAMNCGLPTFATNQGGPAEIIVDEVSGFHINPLDGKKASNKIADFFQKCKEDPMYWNKISTAGLQRIYECYTWQIYATKVLNMGSMYGFWRTMDKEERQAKQRYLQMFYNLQFRKLAKAVPKVGERPEQPTAATVPDRLVSRPKERQVCPLLRNLLKKEQGSC*

>SI004G04220

MASKLTRLHSLRERLGATFSSHPNELIALFSRYVNQGKGMLQRHQLLAEFDALFDSDKEKYAPFEDILRAAQEAIVLPPWVALAIRPRPGVWDYIRVNVSELAVEELSVSEYLAFKEQLVDGQNTSNFVLELDFEPFNASFPRPSMSKSIGNGVQFLNRHLSSKLFQDKESLYPLLNFLKAHNYKGTTMMLNDRIQSLRGLQSSLRKAEEYLLSIPQDTPYSEFNHRFQELGLEKGWGDTAKRVLDTLHLLLDLLEAPDPANLEKFLGTIPMMFNVVILSPHGYFAQSNVLGYPDTGGQVVYILDQVRALEDEMLLRIKQQGLDITPKILIVTRLLPDAVGTTCGQRLEKVIGTEHTDIIRVPFRNENGILRKWISRFDVWPYLETYTEVYRLIFLLDVVHNLDFSVNTEVMHSVLQDVASEIMKEMQAKPDLIIGNYSDGNLVATLLAHKLGVTQCTIAHALEKTKYPNSDIYLDKFDSQYHFSCQFTADLIAMNHTDFIITSTFQEIAGSKDTVGQYESHIAFTLPGLYRVVHGIDVFDPKFNIVSPGADMSVYYPYTETDKRLTAFHPEIEELIYSDVENSEHKFVLKDKNKPIIFSMARLDRVKNMTGLVEMYGKNARLRELANLVIVAGDHGKESKDREEQAEFKRMYSLIDQYNLKGHIRWISAQMNRVRNAELYRYICDTKGAFVQPAFYEAFGLTVIESMTCGLPTIATCHGGPAEIIVDGVSGLHIDPYHSDKAADILVNFFDKCKADPSYWDKISQGGLQRIYEKYTWKLYSERLMTLTGVYGFWKYVSNLERRETRRYLEMFYALKYRSLASAVPLSFD*

>SI004G29780

MASNLSFKRTDSIADSMPDALRQSRYQMKRCFQRYVSKGKRLLKNQQLLEELEKSLDDKVEKEKLVEGFLGYIICSTPEAVVLPPYVAFAVRMNPGIWEYVKVHSDDLSVEGITPSEYLKFKETLYDENWAKDDNSLEVDFGALDLSTPHLTLPSSIGNGLQFVSKFMSSKLGDKPETSMKPLLDYLLSLNYRGEKLMINDIIDTVNKLQTALLLAEVFVSGLPRYTPFAKFEQRFQEWGLEKGWGDTAERCKETLNCLSEVLQAPDPINMEKFFSRVPTIFNIVVFSIHGYFGQEKVLGLPDTGGQVVYILDQVRALEEELLQRIKQQGLKVTPKILVLTRLIPDAKGTKCNVELEPVENTKHCSILRVPFKTEDGKDLRQWVSRFDIYPYLERYAQDSCAKILDILEGKPDLIIGNYTDGNLVASLMSSKLGVTQGTIAHALEKTKYEDSDVKWRDLDQKYHFSCQFTADMIAMNTSDFIITSTYQEIAGSKEKPGQYEHHYAFTMPGLCRYATGINVFDPKFNIAAPGADQSIYFPFTQKQKRLTDLHPQIEELLYSKQDTDEHIGYLADRNKPIIFSMARLDKVKNITGLVEWYGQNRKLRDLVNLVVVAGLLEASQSKDREEIEEINKMHNLIDKYQLKGQIRWIKAQTDRVRNGELYRCIADTKGAFVQPALYEAFGLTVIEAMNCGLTTFATNQGGPAEIIVDGVSGFHINPMNGREASNKIADFFQKCKEDPSYWNKVSTAGLQRIYECYTWKIYATKVLNMGSTYTFWKTLNKEERAAKQRYLQMFYNLQFRNLAKTVPRVFEHPPQTPAGAGPSTVTVVRPKERKPQTRIQRIMTSLMGNKSSTSD*

>SI009G38390

MGEAAGDRVLSRLHSVRERIGDSLSAHPNELVAVFTRLKNLGKGMLQPHQIIAEYNSAIPEAEREKLKDGAFEDVLRAAQEAIVIPPWVALAIRPRPGVWEYVRVNVSELAVEELRVPEYLQFKEQLVEEGPNNNFVLELDFEPFNASFPRPSLSKSIGNGVQFLNRHLSSKLFHDKESMYPLLNFLRAHNYKGMTMMLNDRIRSLSALQGALRKAEEHLSSLPADTPYSDFHHRFQELGLEKGWGDCAKRAQETIHLLLDLLEAPDPSTLEKFLGTIPMVFNVVILSPHGYFAQANVLGYPDTGGQVVYILDQVRAMENEMLLRIKQCGLDITPKILIVTRLLPDATGTTCGQRLEKVLGTEHCHILRVPFRTENGIVRKWISRFEVWPYLETYTDDVAHEIAGELQANPDLIIGNYSDGNLVACLLAHKMGVTHCTIAHALEKTKYPNSDLYWKKFEDHYHFSCQFTTDLIAMNHADFIITSTFQEIAGNKDTVGQYESHMAFTMPGLYRVVHGIDVFDPKFNIVSPGADMSIYFPYTESHKRLTSLHPEIEELLYSQTENNEHKFVLNDRNKPIIFSMARLDRVKNLTGLVELYGRNKRLQELVNLVVVCGDHGNPSKDKEEQAEFKKMFDLIEQYNLNGHIRWISAQMNRVRNGELYRYICDTQGAFVQPAFYEAFGLTVVEAMTCGLPTFATAYGGPAEIIVHGVSGYHIDPYQGDKASALLVDFFEKCKEDSSHWSKISQGGLQRIEEKYTWKLYSERLMTLTGVYGFWKYVSNLERRETRRYLEMLYALKYRTMASTVPLAVEGEPSSK*

>SI009G42350

MSAPKLDRTPSIRDRVEDTLHAHRNELVALLSKYVNKGTCILQPHHILDALDEVQGSEGRALAEGSFLDVLRSAQEAIVVPPFVAIAVRPRPGVWEYVRVNVHELSVEQLTIPEYLCFKEALVDGQHNDPYLLELDFEPFNVSVPRPNRSSSIGNGVQFLNRHLSSIMFRNRDCLEPLLDFLRGHRHKGHVMMLNDRIQSLGRLQSVLTKAEEHLSKLPADTPYSQFAYQFQEWGLEKGWGDTAEHILEMIHLLLDILQAPDPSTLETFLGRIPMIFNVVVVSPHGYFGQANVLGLPDTGGQIVYILDQVRALENEMVLRLKKQGLDVTPKILIVTRLIPDAKGTSCNQRLERISGTQHTYILRVPFRNENGILKKWISRFDVWPYLERFAEDAAGEIAAELQGTPDFIIGNYSDGNLVASLLSYKMGITQCNIAHALEKTKYPDSDIYWKKFDEKYHFSCQFTADIISMNNADFIITSTYQEIAGSKNTVGQYESHTAFTLPGLYRVVHGIDVFDPKFNIVSPGADMSIYFPHTEKAKRLTSFHGSIESLIYDPEQNDEHIGYLDDRSKPILFSMARLDRVKNITGLVEAFAKCSKLRELVNLVVVAGYNDVKKSKDREEIAEIEKMHELIKTYNLFGQFRWISAQTNRARNGELYRYIADTHGAFVQPAFYEAFGLTVVEAMTCGLPTFATLHGGPAEIIEHGISGFHIDPYHPDQAANLMADFFERSKQEPNHWVKISEAGLQRIYEKYTWKIYSERLMTLAGVYGFWKYVSKLERRETRRYLEMFYILKFRELVKTVPLAIDQPQ*

>PAB00010305

MAAPVQRADSVADKLPEALRQNRYQIRKCFSRFVSQGKRILQTQELLNELATIIEDPVERNKIQEGMFGRMLQSTQEVVIVPPFIGLAIRTKPGIWEYASVNDNDLSIEQITVSEYLKLKECLVDEEWPKNDYALELDFEPFNASFPRMARPSSIGNGVHFLSQHLSCRLFHDAQSMQPLLHFLQTCNYCGEKRMVGDSINTVSQLQTALGKAEKILSDLRKDAPYEEFEHRFQDIGLEKGWGNNANNVLHTIHLLLEVLQEPDPIALEKFLGKIPNVFNVVIFSPHGYFGQADVLGLPDTGGQVVYILDQVKALEEELLSRIEQQGLDITPQILVVTRLIPEAQGTRCNQRIEKVLNTQYSQILRVPFKTEKGVLRRWVSRFDVWPYLEKFAEDAANEIVTALQGKPDLIIGNYSDGNLVASLVANKLGIIQCNIAHALEKTKYANSDLNWKKFDEKYHFSCQFTADILAMNNADFIITSTYQEIAGSEDTVGQYESHGAFTLPGQYRVVSGIDVFDPKFNIVSPGADMSIYFPYKEKQSRLTQFHEAIEELLFNPEDTLEHKGFLNDKKKPIIFSMARLDRVKNMTGLVEWFGKNRRLRKLVNLVVVAGFIDSSKSKDREEIAEIEKMHGLIRKYNLNGDFRWICAQKDRIRNGELYRYIADTKGAFIQPALYEAFGLTVIEAMTCGLPTFATCKGGPAEIIIDGVSGFHIDPHNGDEASEKIANFFGKCKRNTNYWNVVSDAGLQRIYDSYTWKIYAEKLINLTNIYGFWKYVSKNNRREIQEYMKLFYNLKFQNLVKNSPKQELYGSHARIKR*

>PAB00011567

MVAATLTRVLSSRERVQDTLFEHRNEIVSLLSRYVAKGKKILQPHDLLDGLAEVTGENDEGQKLRDGPFGDVLRSTQEAIILPPWVVLAVRPRPGVWDYVRVNVDELAVEQLSVAEYLEFKEHLVNGSDKDNYVLELXXXFNASFPRPTRPSSIGSGVQFLNRHLSSRLFRDKESMQPLLDFLRAHNYRGQKLMLNERIQSLPRLRSALVKAEEHLHKFPKDTPYTEFEHKLQEMGLEKGWGDNAEHVLGTIHLLLEILQAPDPSNLETFLGRIPMVFNVVILSPHGYFGQANVLGMPDTGGQVVYILDQVRALESEMLLRIKQQGLDITPEIIVVTRLIPEAHGTTCNQRIEKVSGTQHSRILRVPFRTEKGVLRDWVSRFDVWPYLERFSEDVSNEVTAELKGQPDLIIGNYSDGNLVASLIAHKQGITQCNIAHALEKTKYPDSDIYWKNFEEKYHFSCQFTADLIAMNHADFIITSTYQEIAGSKDTVGQYESHTAFTLPGLYRVVHGIDIYFPYTEKQHRLTALHGSIEELLFNPEQTAEHMCVLNDHKKPIIFSMARLDRVKNMTGLVEWFAKNKRLRGLVNLVVVAGDIDPSKSRDREEVAEIEKMHRLIKEYNLDGQFRWICAQKNRVRNGELYRYICDTKGAFVQPAIYEAFGLTVVEAMTCGLPTFATCNGGPAEIIVDGVSGFHIDPYHGVSASERIADFFEKCKTDPSYWIKISNGGLQRIYERYTWKIYAEKLMTLSGVYGFWKYVSKLERLETRRYLEMFYTLKYRDLVKTVPLAVEESANGIEEKTLSGTRSDQKRLFYYPTFRIYERGLAYFVLGCYVFLMYERGRHSIILMEHYAAAHPDELLMTPIVMY*

>PAB00021357

MCYVCYRYIADGKRILHPQQLFDELAAVIEGQDERKKIQQGAFGNILQCTQEAVIVPPFIGLAIRTKPGIWEYVRVNVENLSIDQLTVPEYLQLKECLVDEQWAKDEYALELDFECFNASLPHMGRSSSVGNGIHFLSKHLASRLFRDGESMQPLLDFMQGHNYQGQKFMVNESINSLSKLQSALSKAEEMLSSLPKDTLYEEFDHRFQDIGLEKGWGENAGHALDMIQSVLEILEAPDPALLEKLLGKIPTVFSVVIFSPHGYFGQADVLGLPDTGGQVVYILDQVKALEEEMLLRIKQQGLDITPQIIVVTRLIPEAQGTKCNQSIEKILNTQHSHILRVPFRTEKGVLRHWVSRFDVWPYLEKFAEDSSKEIIAKLQDKPDLIIGNYSDGNLVASLVAKKLEVTQCNIAHALEKTKYADSDINWKKFDEKYHFSCQFTADILAMNHADFIITSTYQEIAGSKDTVGQYESHAAFTLPGEYRVVSGIDVFNAKFNIVSPGADMTIYFPYTEKQSRLTAFHESIEELLFNPAETTEHMKLVNLAVVGGFIDSSKSKDREEIAEIEKMHGLIKKYSLKGDFRWICAQKDRVRNGELYRYIADTKGAFIQPALYEAFGLTVIEAMTCGLPTFATSKGGPAEIIIDGLSGFHIDPNNGDEASDKIAKFFERCKQEPSYWNKISDAGLQRIYESYTWKIYAEKLINLASVYGFWKYISKSGVHQTQRYMEMFYILKYRNLVKNMPIAKEEPETQIIEKGTRPAMEDNKANGRVASQSGIKRILSTWISTCGMPTCGEDSLVKEKNHT*

>PME00009680

MVAATLNNALSSRERVEDMLSEHRNEIVSLLSRYVAEGKKILQPHQLLDGLEEVIGQNVELQSLRHGLFGEVLRSTQEAIVLPPWIVLAVRPRPGVWEYVRVNVDELAAEQLSVAEYLEFKEHLVNGSIKDDYILELDLEPFNASFPRPTRPSSIGSGVQFLNRHLSSRLFRDKESMQPLLNFLRVHKYRGQKLMLNERVQNLPKLRSALVKAEEHLKKFPKNTPYTEFEHKLQEMGLEKGWGDNVEHVLDTIHLLLEILQAPDPSMLETFLGRIPMVFHVVILSPHGYFGQANVLGMPDTGGQVVYILDQVRALENEMLRRIKQQGLDITPEIIVVTRLIPEAHGTTCNQRIERISGTQHSRILRVPFRTEKGVLRQWVSRFDVWPYLERFSEDVSNEITVELKGQPDLIIGNYSDGNLVASLIAHKQGITQCNIAHALEKTKYPDSDIYWKNFEEKYHFSCQFTADLIAMNHADFIITSTYQEIAGSKDTVGQYESHTAFTLPGLYRVVHGIDVFDPKFNIVSPGADMQIYFPYTEKQHRLTALHGSIEELLFSPEQTTEHMCVLNDRKKPIIFSMARLDRVKNMTGLVEWFAKNKRLRELVNLVVVAGDIDPSKSRDREEVAEIEKMHELVKEYNLNGQFRWICAQKNRVRNGELYRYICDTRGAFVQPALYEAFGLTVVEAMTCGLPTFATCKGGPAEIIVDGVSGFHIDPYHGVSASERIADFFEKCKTDPGHWDKISNGGLQRIYERYTWQIYADRLMTLSGVYGFWKYVSKLERRETRRYLEMFYSLKYRNLVKTVPLAVEESVNGVEEKSAELPVGDALPNGASALVHNH*

>PME00012638

MVAAMLTRALSSRERVQDTLSEHRNEIVSLLSRYVAKGKKILQPHHVLDGLAEVTGGTDEGQKLRDGPFGDVLRSTQEAIILPPWVVLAVRPRPGVWEYVRVNVDELAVEQLSVAEYLEFKEHLVNGSVKDSYVLELDLEPFNASFPRPTRPSSIGSGVQFLNRHLSSRLFHDKENMQPLLDFLRVHNYRGQMLMLNERIQSLPKLRSALVKAEEHLNKFPKDTPYTEFEHKLQEMGLEKGWGDNAEHVLGMIHLLLDLLQAPDPSTLETFLGRIPMVFNVVILSPHGYFGQEKVLGMPDTGGQVVYILDQVRALEHEMLLRIKQQGLDITPEIIVVTRLIPEAHGTTCNQRIEKISGTQHSRILRVPFRTEKGVLRDWVSRFDVWPYLERFSEDVTNEVTAELKGQPDLIIGNYSDGNLVASLIAHKQGITQCNIAHALEKTKYPDSDIYWKNFEEKYHFSCQFTADLIAMNHADFIITSTYQEIAGSKDTVGQYESHTAFTLPGLYRVVHGIDVFDPKFNIVSPGADMQIYFPYTEKQHRLTALHGSIEELLFNPQQTDEHMCVLNDPKKPIIFSMARLDRVKNMTGLVEWFAKNKRLRELVNLVVVAGDIDPSKSKDREEVSEIEKMHQLIKEYNLNGQFRWICAQKNRVRNGELYRYICDTKGAFVQPALYEAFGLTVVEAMTCGLPTFATCKGGPAEIIVDGVSGFHIDPYHGDSVSDRIADFFERCKTDPSYWINISNAGLQRIYEKYTWKIYAEKLMTLSGVYGFWKYVSKLERRETRRYLEMFYTLKYRDLVKTVPLAEEETVDGIEGKSTE*

>PME00027252

MNTEISKTGYLEMSLRSTQEAIVFPPWVVLAVRPRPGVWEYVRVNVDERAVEQLSVAEYLEFKEQLVNESVKDNYVLELDLEPFNASFPRPTQPSSIGSGVQFLNRHLSSRLFHDKDKMQPLLDFLRAHKYQGQRLMLNERIQSLPKLRASLVKAEEHLNKFPEDTPYVEFEHKLQEMGLEKGWGDNAEHVLEMIHLLLEILQAPDPATLETFLGRIPMVFNVVILSPHGYFGQANVLGMPDTGGQVVYILDQVRALESEMLLKIKQQGLDITPEIIVVTRLIPEAHGTTCNQRIEKISGTQHSRILRVPFRTEKGVLRHWVSRFDVWPYLEKFAEDVASEIAAELKGQPDLIIGNYSDGNLVASLISHKQGITQCNIAHALEKTKYPDSDIYWKNFEEKYHFSCQFTADIIAMNTADFIITSTYQEIAGSKDTVGQYESHSAFTLPGLYRVVHGIDVFDPKFNIVSPGADMQIYFPYTEKQRRLTALHGSIEELLFSPEQTAEQMCVLNDHKKPIIFSMARLDRVKNITGLVEWFAKNKRLRELVNLVVVAGDFDPLKSNDREEVAEIEKMHGLIKEYNLNGQFRWICSQKNRVRNGELYRYICDTRGAFVQPALYEAFGLTVVEAMTCGLPTFATRHGGPAEIIVDGVSGFHIDPYHGDSTSELIADFFERCKTDPGHWDAISNAGLQRIYERYTWKIYAERLMTLAGVYGFWKYVSKLGRRETRRYLEMFYILKYRNLVKTVPFAVEENADGIEEKTV*

>PME00028776

MVAATLTRALSSRERVQDTLSEHRNEIVSLLSRYVAKGKKILQPHHLLDGLEEVMGENDELQKLRDGPFGDVIRSTQEAIIFPPWIALAVRPRPGVWDYVRVNVHELAVEQLSVAEYLEFKEHLVNGSIKDNYVLELDLEPFNASFPRPTRPSSIGSGVQFLNRHLSSRLFHGKESMQPLLDFLRAHNYRGQKLMLNERIQSLPRLRSALVKAEEHLKKFPDNTPYTEFEHKLQEIGLEKGWGDNAEHVLDMIHLLLEILQAPDPSTLETFLGKIPMVFNVVILSPHGYFGQANVLGMPDTGGQVVYILDQVRALENEMLLRIKQQGLDITPEIIVVTRLIPEAYGTTCNQRIERISGTQHSRILRVPFRTEKGILHNWVSRFDVWPYLEQFSEDVTNEVTAELKGQPDLIIGNYSDGNLVASLIAHKQGITQCNIAHALEKTKYPDSDIYWKNFEEKYHFSCQFTADLIAMNHADFIITSTYQEIAGSKDTVGQYESHTAFTLPGLYRVVHGIDVFDPKFNIVSPGADMQIYFPYTEKQHRLTALHGSIEELLFNPEQTADHMCVLNDRKKPIIFSMARLDRVKNMTGLVEWFAKNKRLRELVNLVVVAGDIDPSKSRDREEVAEIEKMHTLIKEYNLNGQFRWICAQKNRVRNGELYRYICDTKGAFVQPALYEAFGLTVVEAMTCGLPTFATCNGGPAEIIVDGVSGFHIDPYHGDSATERIADFFEKCKIDSSYWDKISNAGLQRIYERYTWKIYAERLMTLAGVYGFWKYVSKLDRRETRRYLEMFYTLKYRDLVKTVPLAIEESDGIEEKSTE*

>PPI00011864

MVLARLTRVQSSRELVQDTLFEHRNEIVSLLSSYVAQGRKILHPHHLLDGLAEILGEDDELQNLRDGSFGDVLRSTQEAIILPPWVVLAVRPRPGVWEYVRVNVDELAVEQLSVAEYLEFKENLVTGSVKDNYVLELDLEPFNASFPRPTQPSSIGSGVQFLNRHLSSRLFHDKDSMQPFLDFLRAHSYRGQKLMLNERIQSLPKLRSALVKAEEHLNKFPLNTPYMEFEHKLQEMGLEKGWGDNVEHVLDMIRLLLEILQAPDPSTLETFLGRIPMVFNVVILSPHGYFGQANVLGMPDTGGQVVYILDQVRALEKEMLLRIKRQGLDITPQIVVVTRLIPEAYGTTCNQRIERISGTQHSRILRVPFKTDKGVLRKWVSRFDVWPYLERFSEDVSNEINAELQGQADLIIGNYSDGNLVASLIANKQGITQCNIAHALEKTKYPDSDLYWKNFEEKYHFSCQFTADIMAMNHADFIITSTYQEIAGSKDTVGQYESHTAFTLPGHYRVVNGIDVFDAKFNIVSPGADMQIYFPYTEKQRRLTALHDSIEELLFNPEQTAEHMCALNDHKKPIIFSMARLDRVKNMTGLVEWFAKNKRLRELVNLVVVAGDFDPSKSKDREEVAEIEKMHTLIEEYNLNGQFRWICAQKNRVRNGELYRYICDTKGAFVQPALYEAFGLTVVEAMTCGLPTFATCNGGPAEIIVDGVSGFHIDPYHGDNASECIADFFERCKTDPGYWDRISNAGLQRIYERYTWQIYSERLMTLAGVYGFWKYVSKLERRETRRYLEMFYALKYRDLVKTVPLAAEEITDGIEEKSTK*

>PSI00012809

MVAATLTRVLSSRERVQDTLFEHRNEIVSLLSRYVAKGKKILQPHDLLDGLAEVTGENDEGQKLRDGPFGDVLRSTQEAIILPPWVVLAVRPRPGVWDYVRVNVDELAVEQLSVAEYLEFKEHLVNGSDKDNYVLELDLEPFNASFPRPTRPSSIGSGVQFLNRHLSSRLFRDKESMQPLLDFLRAHNYRGQKLMLNERIQSLPRLRSALVKAEEHLHKFPKDTPYTEFEHKLQEMGLEKGWGDNAEHVLGTIHLLLEILQAPDPSNLETFLGRIPMVFNVVILSPHGYFGQANVLGMPDTGGQVVYILDQVRALESEMLLRIKQQGLDITPEIIVVTRLIPEAYGTTCNQRIERVSGTQHSRILRVPFRTEKGVLRDWVSRFDVWPYLERFSEDVSNEVTAELKGQPDLIIGNYSDGNLVASLIAHKQGITQCNIAHALEKTKYPDSDIYWKNFEEKYHFSCQFTADLIAMNHADFIITSTYQEIAGSKDTVGQYESHTAFTLPGLYRVVHGIDVLNPKFNIVSPGADMQIYFPYTEKQHRLTALHGSLEELLFNPEQTAEHMCVLNDHKKPIIFSMARLDRVKNMTGLVEWFAKNKRLRGLVNLVVVAGDIDPSKSRDREEVAEIEKMHRLIKEYNLDGQFRWICAQKNRVRNGELYRYICDTKGAFVQPAIYEAFGLTVVEAMTCGLPTFATCNGGPAEIIVDGVSGFHIDPYHGDSASERIADFFEKCKTDPSYWIKISNGGLQRIYERYTWKIYAEKLMTLSGVYGFWKYVSKLERLETRRYLEMFYTLKYRDLVKTVPLAVEESANGIEEKSIE*

>PSY00009688

MVAATLTRALSSRERVQDTLSEHRNEIVSLLSRYVAKGKKVLQPHHLLDGLAEITGENDEGQKLRDGPFGDVLRSTQEAIILPPWVVLAVRPRPGVWDYVRVNVDELAVEQLSVAEYLEFKEHLVNGSDKDNYVLELDLEPFNASFPRPTRPSSIGSGVQFLNRHLSSRLFRDKESMQPLLDFLRAHNYRGQKLMLNERIQSLPKLRSALVKAEEHLHKFPKDAPYAEFEHKLQEMGLEKGWGDNAEHVLSTIHLLLEILQAPDPSNLETFLGRVPMVFNVVILSPHGYFGQANVLGMPDTGGQVVYILDQVRALENEMILRIKQQGLDITPQIIVVTRLIPEAHGTTCNQRIEKVSGTQHSLILRVPFRTEKGVLRNWVSRFDVWPYLEKFSEDVTNEVTAELKGQPDLIIGNYSDGNLVASLIAHKQGITQCNIAHALEKTKYPDSDIYWKNFEEKYHFSCQFTADLIAMNHADFIITSTYQEIAGSKDTVGQYESHTAFTLPGLYRVVHGIDVFDPKFNIVSPGADMQIYFPYTEKQHRLTALHSTIEELLFNPEQTAEHMCVLNDPKKPIIFSMARLDRVKNMTGLVEWFAKNKRLRELVNLVVVAGDIDPSKSMDREEVAEIEKMHELIKKYNLNGQFRWICAQKNRVRNGELYRYICDTKGAFIQPAIYEAFGLTVVEAMTCGLPTFATCNGGPAEIIVDGVSGFHIDPYHGDSASDRIADFFERCKTDPSYWVKISNGGLQRIYERYTWKIYAEKLMTLSGVYGFWKYVSKLERRETRRYLEMFYTLKYRNLVKTVPLAVEESADGESAE*

>PSY00015731

MVAATLTRVLSSRERVEDTLSEHRNEIVSLLSRYVAKGKKILQPHHLLDGLTEVLGENDELQTLKYGLFGDVLRSTQEAIILPPWIVLAVRPRPGVWDYVRVNVDELAVEQLSVAEYLEFKEHLVDESVKDKYALELDLEPFNESFPRPTRPSSIGSGVQFLNRHLSSRLFHDRESMQPLLDFLRAHSYRGQKLMLNERIQSLSKLRSVLVKAEEHLKKFPKNTPYTEFEYKLQEMGLEKGWGDNAEHVLDMIHLLLETLQAPDPSTLETFLGRIPMVFNVVILSPHGYFGQANVLGMPDTGGQVVYILDQVRALENEMLLRIKHQGLDIKPEIIVVTRLIPEAYGTTCNQRIERIGGTQHSRILRVPFRTEKGVLQKWVSRFDVWPYLERFSEDVLNEVTAELKGQPDLIIGNYSDGNLVASLISERQGITQCNIAHALEKTKYPDSDIYWKKYEEKYHFSCQFTADLIAMNHADFIITSTYQEIAGSKDTVGQYESHTAFTLPGLYRVVHGIDVFDPKFNIVSPGADMQIYFPYTEKQHRLTALHGSIEELLFNPEQTAEHMCILNDRKKPIIFSMARLDRVKNMTGLVEWFAKNKRLRELVNLVVVAGDIDPSKSRDREEVVEIEKMHTLIKEYNLNGQFRWICAQKNRVRNGELYRYICDTRGAFIQPALYEAFGLTVVEAMTCGLPTFATCNGGPAEIIVDGVSGFHIDPYHGASASEYIVEFFERCKSDPSCWDKISNAGLQRIYERYTWQIYAEKLMTLSGVYGFWKYVSKLERRETRRYLEMFYTLKYRDLVKTVPLAVEESADGLEEKSAE*

>PTA00028412

MASSMQRSESITDALPEIVRQNRFLLRKLFPRYAVEGKRILHPQQLIEELAAVIESQDERKKILQGAFGHILECAQEAVVVPPFIGFAIRTKPGIWEYVRVNVENLSTYQLTVAEYLQLKECLVDERWYLVSSVSANAEFEVRMPPWSNDEYALELDFECFNASFPRMRRPSSIGNGIHFLSKHLSSRLFRDGDSMQPLLDYMQEHKYQGQKFMVNESINSLPKLQSALSKAEDILSNLPKDTRYEEFDYGFQNIGLARGWGDNAGRALDMIQSVLKNLEAPDPAILEKFLGKIPTVFSVVIFSPHGYFGQADVLGLPDTGGQVVYILDQVKALEEEMLLRIKQQGLEITPQIIVVTRLIPEAQGTKCNQKIEKILNTQHSQILRVPFRTEKGVLRHWVSRFDVWPYLETFAEDSSKEIIAKLQDKPDLIIGNYSDGNLVASLVSKKLEVTQCNIAHALEKTKYADSDINWKKFDEKYHFSCQFTADILAMNHADFIITSTYQEIAGSKDTVGQYESHAAFTLPGEYRVVSGIDVFNAKFNIVSPGADMSIYFPYTEKQRRLTAFHESIQELLFNPTESTEHIGFFSDRKKPIIFSMARLDRVKNLSGLVEWFGKNERLRKLVNLAVVGGFIDSSKSKDREEIAEIEKMHGLIKKYSLRGNFRWICAQKDRVRNGELYRYIADTKGAFIQPALYEAFGLTVIEAMTCGLPTFATSKGGPAEIIVDGLSGFHIDPNNEDETSDKIANFFERCKREPSYWNKVSDGGLQRIYESYTWKIYAENLINLASVYRFWKYISNREMHQSQRYMEMFYILKYRNLVKNFPTAKEESEAQIIEKGTRPAAEDNKADRRAAGKSGIKRILSACVSTCGMPNCGEDSLLKEKEPT*

>PTA00049304

MVAATLTRALSSRERVQDTLSEHRNEIVSLLSRYVAQGKKILHPHHLLDGLAEIIGENNEPHKLRDGPFGDVLRSTQEAIILPPWVVLAVRPRPGVWDYVRVNVDELAVEQLSVAEYLEFKEHLVNGSVKDNYVLELDLEPFSASFPRPTRPSSIGSGVQFLNRHLSSRLFRDKESMQPLLDFLRAHNYRGQKLMLNERIQSMPKLRSALVKAEEHLNKFPLNTPYTKFEHKLQEMGLEKGWGDNAEHVLDMIHLLLEILQAPDPSTLETFLGKIPMVFNVVILSPHGYFGQANVLGMPDTGGQVVYILDQVRALENEMLLRIKQQGLDITPEIIVVTRLIPEAYDTTCNQRIEKISGTQHSRILRVPFRTEKGVLRNWVSRFDVWPYLERFSEDVSNEITAELKGQADLIIGNYSDGNLVASLIAHKQGITQCNIAHALEKTKYPDSDLYWKNFEEKYHFSCQFTADLIAMNTADFIITSTYQEIAGSKGTVGQYESHTAFTLPGLYRVVHGIDVFDPKFNIVSPGADMQIYYPYTEKQHRLTTLHRTIEELLFSPEQTAEHMCVLNDRKKPIIFSMARLDRVKNMTGLVEWFAKNKRLRELVNLVVVAGDIDPSNSKDREEVAEIEKMHRLIKEYNLNGQFRWICAQKNRVRNGELYRYICDTRGAFVQPALYEAFGLTVVEAMTCGLPTFATCNGGPAEIIVDGVSGFHIDPYHGDYASECIAEFFERCKTDPGYWDKISNAGLQRIYEKYTWKIYSEKLMTLAGVYGFWKYVSKLERRETHRYLEMFYTLKYRDLVKTVPLAVEEIADGTEEKTTA*

>PTA00057970

MVTAMLNRALSSLERVEDTLSERRNETVSLLSRYISRGKKILQPHQLLDGLAELIGQNDERQNLHFFGLFGDVLKSTQEAIILPPWVVLAVRPRPGVWEYVRVHVDELAVEQLSATKYLEFKEHLVDESFKNNYVLELDLEPFNASFPRPTRPSSIGSGVQFLNRHLSSRLFHGKESMQPLLNFLLAHKYRGQGVGRLQEMGFEKGWGDNVEHVLDMIHLLSEILQAPDPSTLEMFLEKIPMVFNVVILSPHGYFGQANVLGMPDTGGQVVYILDQVRALENEMLLRIKQQGLDITPQIIVVTRLIPEAHGTICNQRIERVIGTQHSRILRVPFRTEKGILRNWVSRFDIWPYLERFAEDVSSEVTAELNGQPDLIIGNYSDGNLVASLIANKQGITQCNIAHALEKTKYPNSDIYWKNFEEKYHFSCQFTADLIAMNHADFIITSTYQEIAGSKDTVGQYESHTAFTLPGLYRVIHGIDVFDPKFNIVSPGADMQIYFPYTEKQRRLTALHDSIEELLFSFEQTTEHMCALNDVKKPIIFSMARLDRVKNITGLVEWFAKNKRLRELVNLVVVAGDIDPLKSRDREEVAEIEKMHRLIKEYNLNGQFRWICAQKNRVQNGELYRYICDTRGAFIQPALYEAFGLTVVEAMTCGLPTFATCNGGPAEIIVDGVSGFHIDPYHGDSASEHIVKFFERCQTDPTHWDKISSAGLQRIYERYTWQIYAKRLMTLSGVYGFWKYVSKLERQETRRYLEMFYILKYRNLVSVVAILDWLKLCHYLLKKVSMGLKIKV*

>TBA00005121

MVTATLSRVVSMRERVEDTLSEHRNEIVALLSRYVAQGKSILQPHHLLDGLAEVKGESDEHEKLKDGLFGDVLRSTQEAIILPPMVALAVRPRPGVWEYVRVNVDELSVDQLSVSEYLEFKEHLVNGSVKDNYVLELDLEPFNSSFPRPTRPSSIGSGVQFLNRHLSSRLFRDKESMQPLLDFLRQHNFRGQRLMLNERIQNLPKLRAALVKAEEYLHKLPKDAPYAEFEHKFQEMGLEKGWGDNSERVLDMIHLLLENLQAPDPSTLEKFLGRIPMVFNVVILSPHGYFGQANVLGMPDTGGQVVYILDQVRALENEMLLRIKQQGLDITPEILVVTRLIPEAHGTTCDQRIERISGTQHSRILRVPFKTEKGILRQWVSRFDVWPYLETFAEDAYNEITAELQAPPDLIIGNYSDGNLVASLIAHKQGITQCNIAHALEKTKYPDSDIYWKNFEEKYHFSCQFTADLIAMNHADFIITSTYQEIAGSKDTVGQYESHTAFTLPGLYRVVHGIDVFDPKFNIVSPGADMRIYFPYTDKQHRLTALHSSIEKLLFSPEQTAEHIGFLKDPKKPIIFSMARLDRVKNMTGLVEWFAKNSRLRDLVNLVVVAGDIDPSKSRDREEIDEIEKMHRLMKEYNLSGQFRWIVAQKNRVRNGELYRYICDTRGAFIQPALYEAFGLTVVEAMTCGLPTFATCKGGPAEIIVDGVSGFHIDPYHGESASEKIADFFERCKTEPSYWDAISNAGLQRIYERYTWQIYADTLVNLAGVYGFWKYVSKLERRETRRYLEMFYILKYRNLVKTVPLAIHEEHVDGVEEKTASAVANNH*
